# Intramolecular Cyclization of Azido-Isocyanides Triggered by the Azide Anion: An Experimental and Computational Study

**DOI:** 10.1021/acs.joc.3c00558

**Published:** 2023-06-20

**Authors:** Mateo Alajarin, Guillermo Cutillas-Font, Carmen Lopez-Leonardo, Raul-Angel Orenes, Marta Marin-Luna, Aurelia Pastor

**Affiliations:** † Departamento de Química Orgánica, Facultad de Química, Regional Campus of International Excellence “Campus Mare Nostrum”, Universidad de Murcia, E-30100 Murcia, Spain; ‡ ACTI, Universidad de Murcia, E-30100 Murcia, Spain

## Abstract

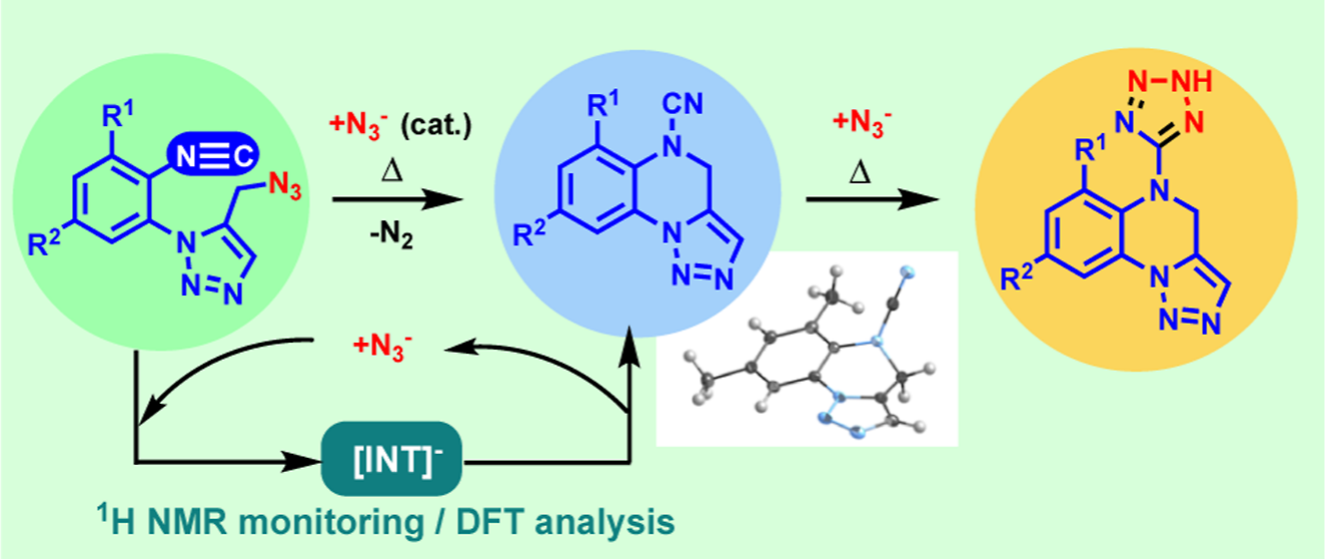

This work describes the unprecedented intramolecular cyclization occurring in a set of α-azido-ω-isocyanides in the presence of catalytic amounts of sodium azide. These species yield the tricyclic cyanamides [1,2,3]triazolo[1,5-*a*]quinoxaline-5(4*H*)-carbonitriles, whereas in the presence of an excess of the same reagent, the azido-isocyanides convert into the respective C-substituted tetrazoles through a [3 + 2] cycloaddition between the cyano group of the intermediate cyanamides and the azide anion. The formation of tricyclic cyanamides has been examined by experimental and computational means. The computational study discloses the intermediacy of a long-lived *N*-cyanoamide anion, detected by NMR monitoring of the experiments, subsequently converting into the final cyanamide in the rate-determining step. The chemical behavior of these azido-isocyanides endowed with an aryl-triazolyl linker has been compared with that of a structurally identical azido-*cyanide* isomer, experiencing a conventional intramolecular [3 + 2] cycloaddition between its azido and cyanide functionalities. The synthetic procedures described herein constitute metal-free approaches to novel complex heterocyclic systems, such as [1,2,3]triazolo[1,5-*a*]quinoxalines and 9*H*-benzo[*f*]tetrazolo[1,5-*d*][1,2,3]triazolo[1,5-*a*][1,4]diazepines.

## Introduction

Isocyanides are versatile building blocks in organic chemistry.^1^ The unique properties of isocyanides come from their formally divalent carbon, which endow them with the ability to react with nucleophiles and electrophiles yielding respective α-adducts.^2^ Isocyanides have been extensively used in multicomponent reactions^3−5^ and transition-metal-catalyzed insertion processes.^6−8^ Azides are also valuable intermediates in organic synthesis.^9^ They have found new applications in peptide chemistry, combinatorial chemistry, and heterocyclic synthesis.^10^ Recently, the Staudinger ligation protocol based on the reactivity of azides with phosphanes has found a relevant place in the field of bioorthogonal chemistry.^11^

The inclusion of both isocyanide and azide functionalities in the same molecule allows to combine the reactivity of these two groups in an orthogonal manner. A good example is the tandem Ugi–Click sequence, useful to generate routes to complex heterocycles.^12−14^ Within this type of bifunctional compounds, 1-azido-2-isocyanoarenes are the most studied. Tungsten, molybdenum, chromium, ruthenium, and platinum complexes with 1-azido-2-isocyanoarenes as ligands have been used to prepare benzimidazole-derived carbenes.^15−18^ The reaction of 1-azido-2-isocyanoarenes with phenylacetaldehyde/DBU and further treatment with the Togni reagent yields [1,2,3]triazolo[1,5-*a*]quinoxalines.^19^ Benzimidazoles can also be synthesized from 1-azido-2-isocyanoarenes and diphenyl phosphine in the presence of manganese(II) acetate^20^ or by reaction with diazonium salts.^20^

**Scheme 1:**
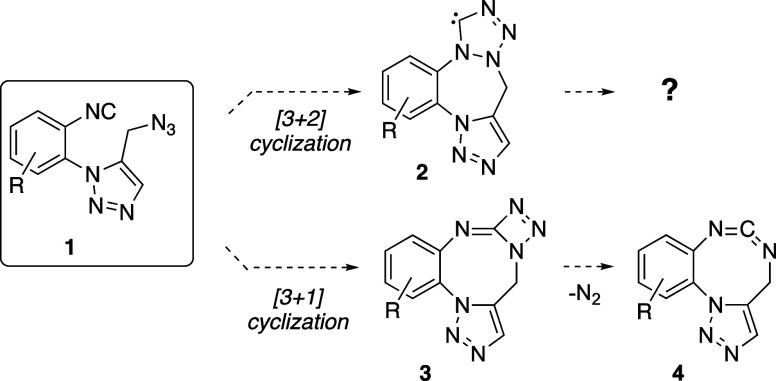
Putative Reaction Products Resulting from Alternative Intramolecular [3 + 2] or [3 + 1] Cyclizations of **1**

Following our report on an easy entry into heterocyclic building blocks for drug discovery,^21^ we envisioned that a similar synthetic methodology could give us access to new scaffolds endowed with isocyanide and azide functionalities adequately placed for reacting one another in an intramolecular way, thus yielding complex nitrogenated heterocycles. To our knowledge, there is only one reported example of azido-isocyanides in which both functional groups participate in metal-free intramolecular reactions, the intramolecular cyclization of 2-[1-azidobenzyl]-1-isocyanobenzene in the presence of sodium hydride to give 4-phenylquinazoline.^22^ In order to discover new intramolecular cyclizations involving both functional groups, we designed azido-isocyanides **1** with an aryl-triazolyl moiety as the linker of both functionalities (Scheme 1).

At the start of the present investigations, we envisaged two putative reaction paths between the azido and isocyano functions of compounds **1** (Scheme 1), either a [3 + 2] cyclization to give presumably unstable carbenes **2** or an alternative [3 + 1] cyclization by α-addition of the azido group to the carbon atom of the isocyanide to give fused triazetimines **3** or cyclic carbodiimides **4** by further N_2_ extrusion. These two reaction paths are unprecedented, as well as its reaction intermediates and products.

Before carrying out the synthetic experiments, we performed an exploratory computational study for testing the ability of compound **1a** (R = H, Scheme 1) to undergo those cyclizations. The results of the computations showed that carbene **2a** is highly unstable and we failed in all the attempts to locate the transition structure of the intramolecular [3 + 2] process. However, the alternative [3 + 1] cyclization to give **3a** and its subsequent transformation into the carbodiimide **4a** by loss of nitrogen seem to be feasible provided the reaction is conducted under strong thermal conditions (see the Supporting Information).

Herein, we disclose our results on the synthesis of azido-isocyanides **1** which proved to be not so simple as anticipated and that laterally led to novel complex heterocyclic systems.

## Results and Discussion

The route toward azido-isocyanides **1** started with the synthesis of intermediates **7a–d** by the reaction of 1-azido-2-isocyanoarenes **5a–d**(19) with the α-ketophosphorane **6** (Table 1). This methodology for preparing 1,2,3-triazoles, originally described by Harvey,^23^ and L’Abbé,^24^ is based on the 1,3-dipolar cycloaddition of the azido group to the double bond of the betainic form of **6** and further elimination of triphenylphosphine oxide. By using this procedure, the resulting 1,5-disubstituted 1,2,3-triazoles **7a–d** were obtained in medium to good yields (Table 1).

**Table 1:**
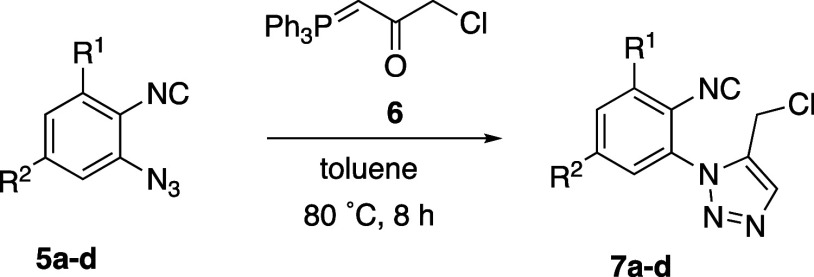
Synthesis of 5-Chloromethyltriazoles **7** from 1-Azido-2-isocyanoarenes **5**

entry	compound	R^1^	R^2^	yield (%)^a^
1	**7a**	H	H	55
2	**7b**	Me	Me	99
3	**7c**	Me	H	89
4	**7d**	Cl	Me	95

a After isolation by column chromatography.

**Figure 1:**
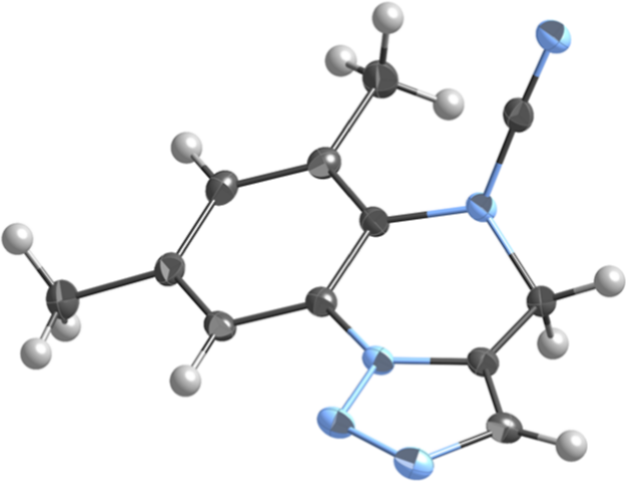
X-ray structure of the cyanamide **8b**.

**Scheme 2:**
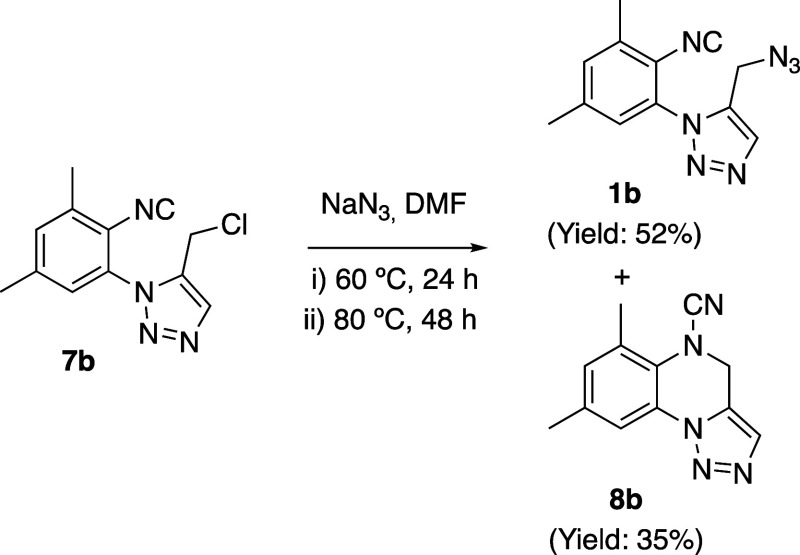
Thermal Treatment of **7b** in the Presence of NaN_3_ Gives a Mixture of **1b** and the Unexpected **8b**

Obviously we expected that the azido-isocyanide **1b** (Scheme 2) would be readily available from the 5-chloromethyl triazole **7b** by chloride substitution with the azido anion.^10^ However, when **7b** was treated with sodium azide in dimethylformamide (DMF) at 60 °C for 24 h, thin-layer chromatography (TLC) analysis (silica gel, 1:2 AcOEt/hexane) showed the formation of a new main product but also apparently a residual spot of starting material. Hence, the reaction mixture was further stirred at 80 °C for an additional period of 48 h. Even though the conversion seemed still uncomplete by TLC, we proceeded to isolate the two species (work-up consisting of water addition and further extraction with AcOEt). The less polar compound turned out to be not the starting material but the pursued azidomethyl triazole **1b**. Surprisingly, the ^13^C NMR spectrum of the second, more polar species showed the disappearance of the peak around 170 ppm attributable to the isocyanide carbon atom (172.2 ppm in **7b**) and the rise of a new quaternary peak at 112.9 ppm. In its infrared (IR) spectrum, the lack of the typical bands assignable to the isocyanide and azide functions close to 2100 cm^–1^ as well as the appearance of a band at 2220 cm^–1^ gave us a first indication of the formation of a cyanamide fragment at the expense of the azido and isocyano groups, a hint that was confirmed by the unequivocal structural determination of this second product as **8b** with the aid of X-ray crystallography (Scheme 2 and Figure 1).

Afterward, under smoother reaction conditions (treatment of **7b** with sodium azide in DMF at 25 °C), the 5-azidomethyl triazole **1b** was obtained in 93% yield (Table 2, entry 2). This reaction is general for the 5-chloromethyltriazoles **7a–d**, and thus, the azido-isocyanides **1a–d** were obtained in yields ranging 52–93% (Table 2).

**Table 2:**
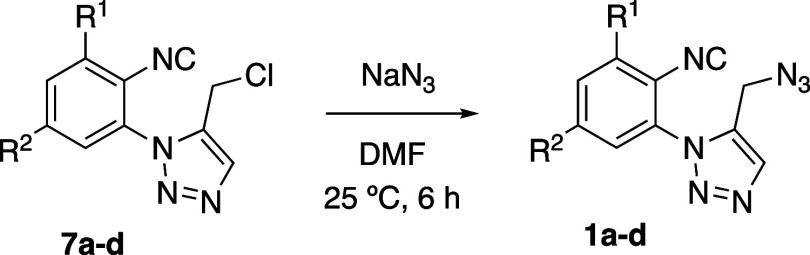
Conversion of 5-Chloromethyltriazoles **7a–d** into 5-Azidomethyltriazoles **1a–d**

entry	compound	R^1^	R^2^	yield (%)^a^
1	**1a**	H	H	60
2	**1b**	Me	Me	93
3	**1c**	Me	H	91
4	**1d**	Cl	Me	52

a After isolation by column chromatography.

Next, we studied the serendipitous formation of cyanamide **8b** and the role of sodium azide in that process (Table 3). First, we checked the thermal stability of azido-isocyanide **1b** by heating in DMF solution at 120 °C for 48 h. Under these conditions, the starting material was recovered unaltered (Table 3, entry 1), thus discarding our previous expectations summarized in Scheme 1. Afterward, we reacted **1b** with one equivalent of sodium chloride, a secondary product in the reaction of **7b** with sodium azide, in DMF at 60–80 °C for 48 h. Once again, no reaction took place (Table 3, entry 2). We conducted three more experiments in the presence of 0.5, 1.0, and 1.5 equiv of sodium azide, thus obtaining cyanamide **8b** in yields ranging 34–67% (Table 3, entries 3–5). These assays not only confirmed the key role of sodium azide in the formation of cyanamide **8b** but also the advantage of using sub-stoichiometric amounts of this reagent to maximize the yield of the conversion **1b** → **8b**.

**Table 3:**
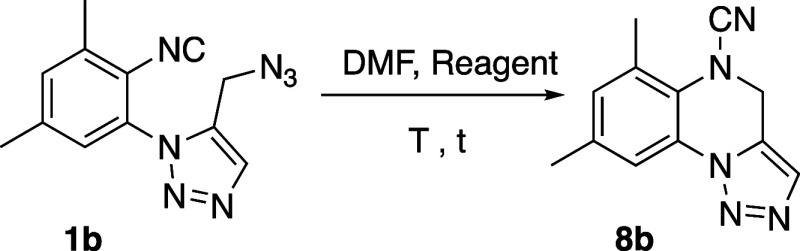
Conversion of 5-Azidomethyltriazole **1b** into Cyanamide **8b** under Different Reaction Conditions

entry	reagent	equiv	*T* (°C)	*t* (h)	yield (%)^a^
1			120	48	
2	NaCl	1	60/80	24/24	
3	NaN_3_	0.5	80	48	67
4	NaN_3_	1.0	80	48	46
5	NaN_3_	1.5	80	48	34

a After isolation by column chromatography.

**Figure 2:**
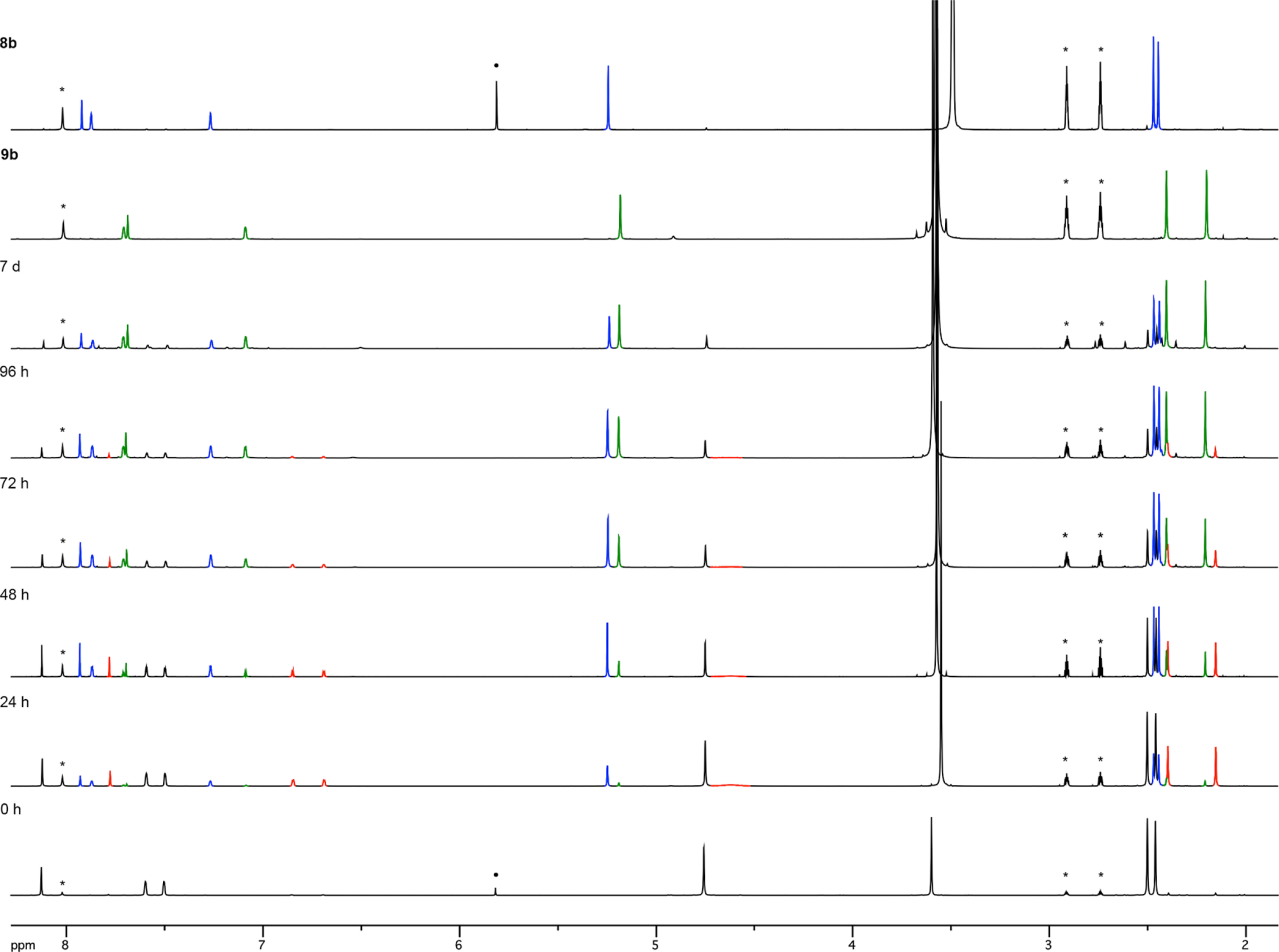
^1^H NMR (400 MHz) monitoring of the reaction of 5-azidomethyltriazole **1b** (black peaks) with NaN_3_ (0.5 equiv) in DMF-*d*_7_ at 80 °C. Color code: **1b** (black), **LLI** (red), **8b** (blue), and **9b** (green). (*) Solvent residual peaks and (●) dichloromethane.

**Scheme 3:**
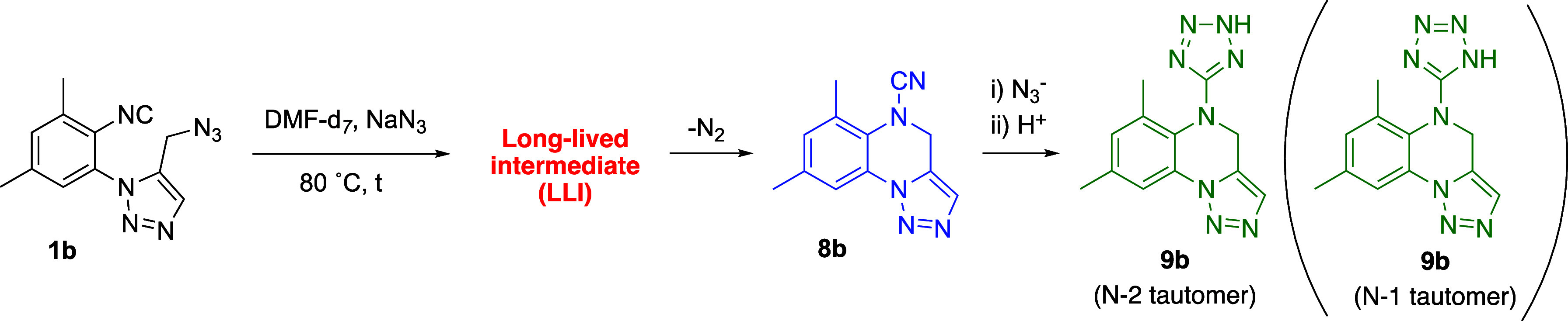
Transformation of 5-Azidomethyltriazole **1b** into **8b** and **9b** in the Presence of Sodium NaN_3_

To gain further insights into this process, we monitored the reaction of **1b** in the presence of 0.5, 1.0 and 1.5 equiv of sodium azide by ^1^H NMR spectroscopy (Figure 2, Scheme 3, and Table 4). In the presence of 0.5 equiv of NaN_3_ (DMF-*d*_7_, 80 °C) the cyanamide **8b** is neatly apparent in the reaction mixture (blue peaks) after 48 h (Figure 2). However, resonances for two new products also appear in this spectrum (red and green peaks). The red peaks weaken over time and totally disappeared after 7 days. These peaks were assigned to a long-lived intermediate (**LLI**) prior to the formation of **8b** (Scheme 3). Remarkably, the resonances of the aromatic protons of **LLI** are considerably shifted to lower chemical shifts compared to those of **1b** and **8b**. The green-colored signals increase over time and become the most intense ones after 7 days (Figure 2 and Table 4, entry 2).

**Table 4:** Conversion Rates of **1b** in the Presence of Different Amounts of NaN_3_ (DMF-*d*_7_, 80 °C) and Ratios of **8b** and **9b**^a^

entry	NaN_3_ (equiv)	*t*	conv. **1b** (%)^b^	**8b/9b**
1	0.5	24 h	52	86:14
2	0.5	7 d	87	41:59
3	1.0	24 h	65	72:27
4	1.0	7 d	95	12:88
5	1.5	24 h	73	66:33
6	1.5	7 d	>95	<5:>95

a Obtained from the ^1^H NMR spectra (400 MHz).

b ±5% of error.

These green-colored signals were assigned to the N-2 tautomer of the tetrazole **9b** (see below), which presumably would form through a 1,3-dipolar cycloaddition between the azide anion and the cyano group of **8b** followed by protonation.^25,26^ According to this hypothesis, the proportion of tetrazole **9b** in the mixture increases when the reaction is conducted with greater amounts of sodium azide (Table 4, entries 3–5). In fact, **9b** was the exclusive reaction product after 7 days in the presence of 1.5 equiv of this reagent (Table 4, entry 6) (for a complete picture of the three monitoring experiments see Figures S3–S5 in the Supporting Information).

Two additional experiments confirmed that tetrazole **9b** formed by the reaction of **8b** with NaN_3_ and not from a common precursor of both species **8b** and **9b**. Thus, **8b** was heated at 80 °C in DMF-*d*_7_ in either lack or presence of sodium azide (1.0 equiv). In the first experiment, **8b** was recovered unaltered, whereas in the second one tetrazole **9b** quantitatively formed. At this point, we wondered why we neither detect **9b** in the reaction media nor isolated it when conducting the reaction in a flask-scale (Scheme 2). We reasoned that due to the high acidity of tetrazole derivatives, with p*K*_a_ values around 5,^27,28^ and without an acidic work-up (see above), **9b** should remain in the aqueous phase in the form of its conjugated base, not being transferred into the organic phase and thus avoiding its detection in the TLC analyses of this phase.

The identity of **9b** was confirmed by 2D NMR (HMBC, HSQC and NOESY, see the Supporting Information). Moreover, computational methods, combined with the experimental NMR data, allowed us to determine the tautomeric form of the tetrazole ring, N-1 or N-2, present in its DMSO-*d*_6_ solution (Scheme 3).^29^ Thus, the ^1^H and ^13^C NMR data of **9b** were compared with those calculated from the corresponding geometry optimized structures of both tautomeric forms (Supporting Information).^30^ The best fit between theoretical and experimental data was found for the N-2 tautomer and the most revealing information is the chemical shift of the carbon atom at the tetrazole ring, 164.4 ppm (calculated) and 163.6 ppm (experimental), far enough from the calculated value for the N-1 tautomer, 155.3 ppm.^31^ Moreover, the observed chemical shift of this carbon atom agrees well with those found in the literature for 2*H*-tetrazole derivatives.^32^ Nevertheless, we cannot discard a fast equilibrium between the N-1 and N-2 tautomers with the N-2 one as the main component.^33^ In fact, evidence of fast proton exchange is revealed by the ^1^H NMR spectra, in which the tetrazole proton shows as a broad signal (**9c**) or was not observed (**9a–b** and **9d**).

With all these data in our hands, the synthesis of cyanamides **8a–d** and tetrazoles **9a–d** was optimized. Thus, the treatment of **1a–d** with a substoichiometric amount of sodium azide at 80 °C led to cyanamides **8a–d** in yields ranging 25–67% (Table 5). From these data, it should be noted that the presence of a methyl group (R^1^) next to the isocyanide group provided the better yields. Alternatively, the same reaction in the presence of an excess of sodium azide and longer reaction times gave tetrazoles **9a–d** (39–81%). Thus, either cyanamides **8a–d** or tetrazoles **9a–d** can be obtained from **1a–d** just by tuning the amount of sodium azide. Overall, these routes allow the access to functionalized triazoloquinoxalines,^34^ compounds with interesting pharmacological properties,^35−38^ without the involvement of a transition-metal^39−43^ some of which are further decorated with a tetrazole ring.^44^

**Table 5:**
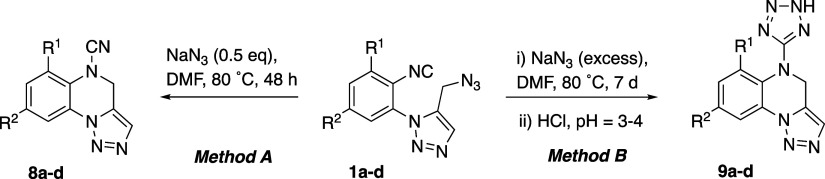
Alternative Methodologies (A or B) for the Conversion of Compounds **1a–d** into Cyanamides **8a–d** and Tetrazolyl Derivatives **9a–d**

entry	compound	R^1^	R^2^	yield **8** (%)	yield **9** (%)^a^
1	**a**	H	H	31	39
2	**b**	Me	Me	67	80
3	**c**	Me	H	41	81
4	**d**	Cl	Me	25^b^	57

a After isolation by column chromatography.

b The cyanamide **8d** was impurified with a 10% of starting material (**1d**).

**Scheme 4:**
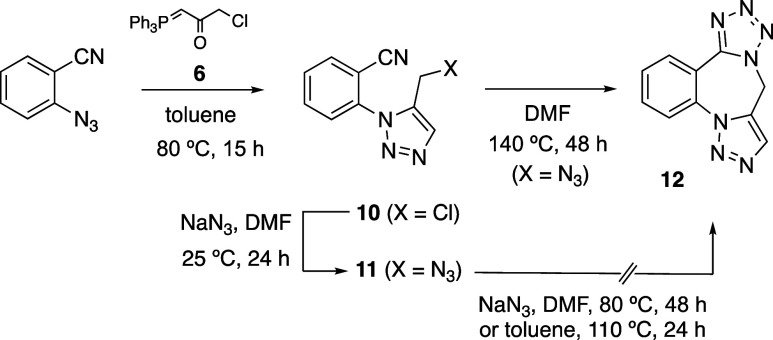
Three-Step Synthesis of 9*H*-Benzo[*f*]tetrazolo[1,5-*d*][1,2,3]triazolo[1,5-*a*][1,4]diazepine (**12**) from 2-Azidobenzonitrile

At this point, we were interested in comparing the behavior of isocyanide **1a** with its isomeric cyanide (Scheme 4). Thus, 2-azidobenzonitrile was reacted with the α-ketophosphorane **6** under the usual reaction conditions to obtain the 5-chloromethyltriazole **10** in excellent yield (91%). The corresponding 5-azidomethyltriazole **11** was prepared by the reaction with sodium azide in DMF at 25 °C (82%). By contrast to what we observed for **1a**, the reaction of **11** in the presence of sodium azide in DMF at 80 °C for 48 h, led to the recovery of the unchanged starting material. Heating at reflux a toluene solution of **11** for 24 h was also unsuccessful for converting this azido-cyanide into its [3 + 2] cycloadduct, the tetracyclic 1,4-diazepine **12**,^45−48^ which however was obtained in 66% yield after heating **11** at 140 °C in DMF solution for 48 h.

**Scheme 5:**
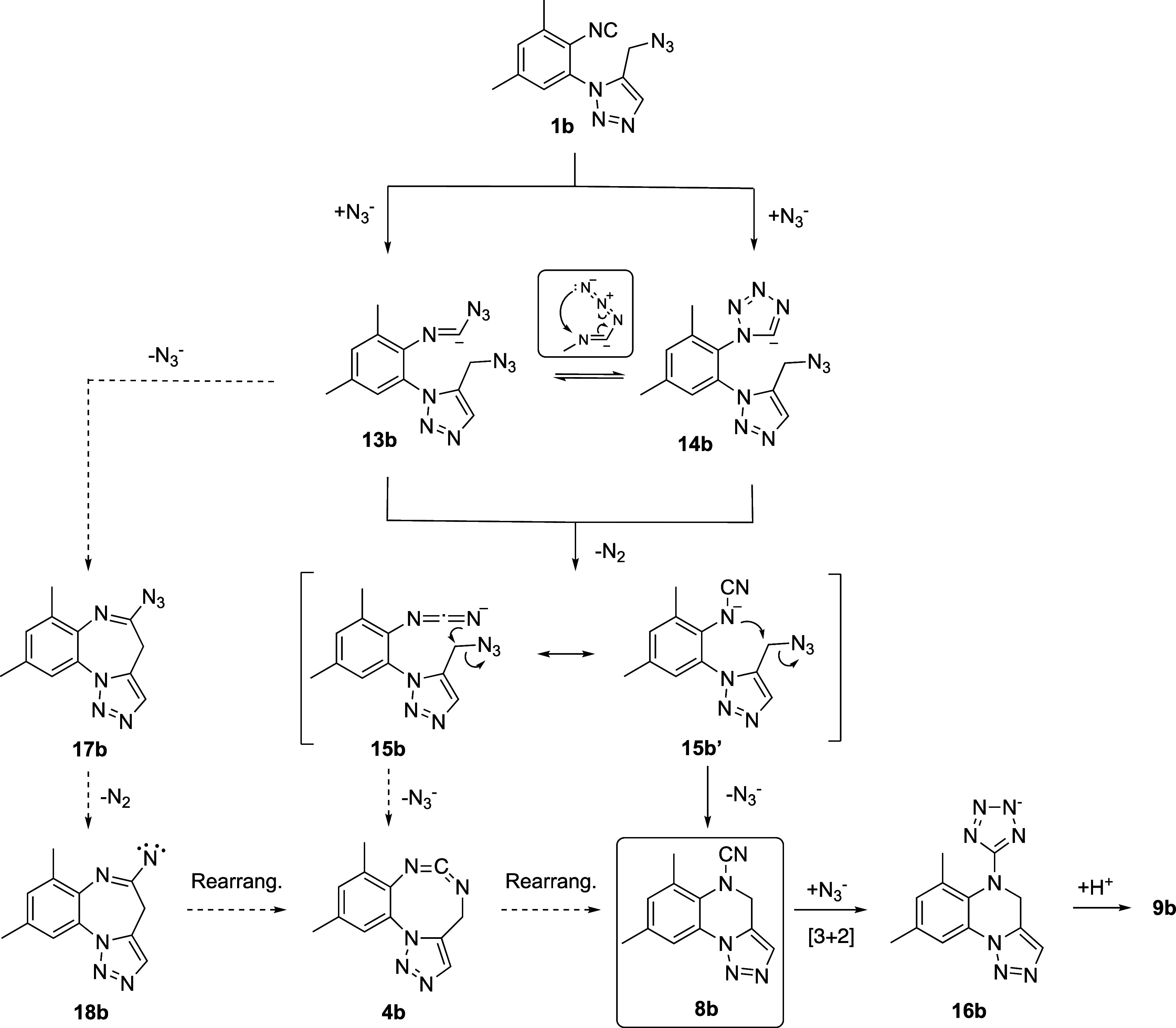
Putative Mechanism for the Conversion of 5-Azidomethyltriazole **1b** into the Tetrazolyl Derivative **9b**

Plausible mechanistic pathways for explaining the formation of cyanamides **8** from 5-azidomethyltriazoles **1** and sodium azide are outlined in Scheme 5, with **1b** as the model substrate. Initially, the addition of the azide anion to the isocyanide functionality of **1b** would lead to the azidoimidoyl anion **13b**.^49,50^ Alternatively, the [3 + 2] cycloaddition between the isocyanide moiety and the azide anion could afford the tetrazolide anion **14b**.^50^ Both anionic species, **13b** and **14b**, could equilibrate through an electrocyclic ring closure/opening.^51,52^ The thermal activation of the experimental process (80 °C) should favor the loss of molecular nitrogen from **13b** or **14b**, thus leading to the carbodiimide anion **15b**, which is more accurately represented as a resonance hybrid between structures **15b** and the cyanamide anion **15b′**. The intramolecular nucleophilic substitution of **15b** with loss of the azide anion would bring about the formation of the final tricyclic cyanamide **8b**. In fact, the ring fragmentation of 1-aryl-5-tetrazolyllithium derivatives at temperatures above −50 °C to give cyanamide anions has been previously reported.^53,54^ From this point, the formation of **9b** is easily explained through a 1,3-dipolar cycloaddition between the azide anion and the cyano group of **8b** with further protonation of the resulting tetrazolide anion **16b**.^25,26^ Two experimental facts support this mechanistic proposal, (i) only substoichiometric amounts of the azide anion are necessary to form **8b** from **1b**, and (ii) the quantitative formation of the tetrazole **9b** cannot take place unless one equivalent of azide anion is present in the reaction mixture.

Additional alternative reaction paths leading from **13b/14b** to the experimental products **8b** and **9b** are also summarized in Scheme 5. The intramolecular nucleophilic substitution of the azido group at the side-chain of **13b** by its azidoimidoyl anionic carbon atom would afford the cyclized imidoylazide **17b**, a plausible precursor of the imidoyl nitrene **18b** after thermally activated loss of nitrogen. Ring expansion of this latter species by nitrene insertion would provide the cyclic carbodiimide **4b**, which could also form by cyclization of **15b**. A skeletal reorganization of **4b** by 1,3-shift of the methylenic carbon atom could then lead to the final cyanamide **8b**. Nevertheless, these latter alternative reaction pathways seem to be less plausible than that via **15b′**. On the one hand, the reported formation of nitrenes from azides usually occur under FVT or photolytic conditions.^55^ Moreover, imidoyl nitrenes have been reported to rearrange into cyclic carbodiimides but also under photolytic conditions.^55^ On the other hand, the formation of cyanamides from the corresponding carbodiimides^56^ has exclusively been observed by photoisomerization or pyrolysis^57,58^

**Figure 3:**
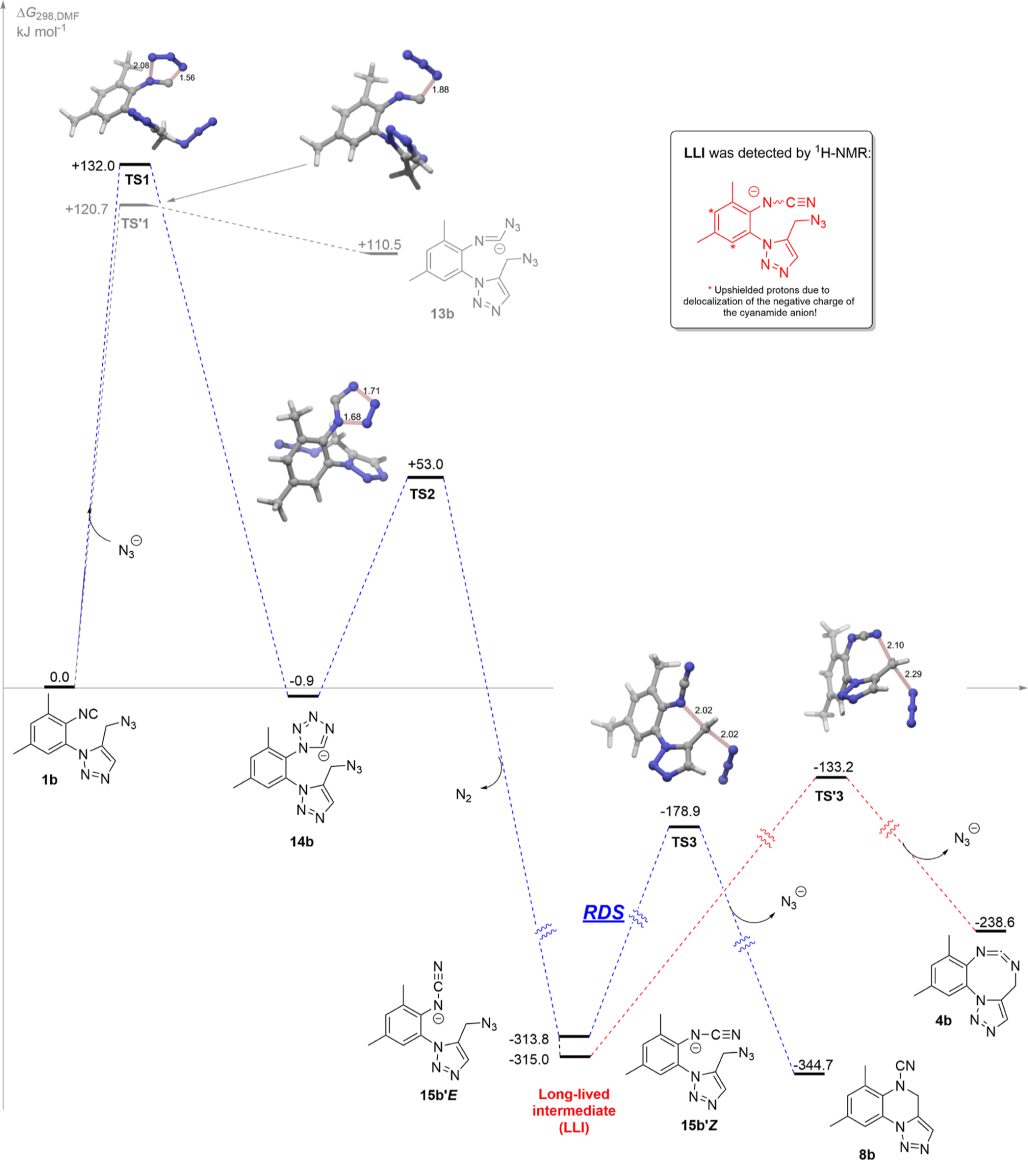
Computed mechanism for the conversion of 5-azidomethyltriazole **1b** into the cyanamide **8b**.

In order to get a deeper insight on the mechanism of the transformation of **1b** into **8b**, we conducted a computational study at the PCM(DMF)/WB97X-D/6-311++g(d,p)//PCM(DMF)/WB97X-D/6-31+g(d,p) theoretical level (Figure 3). Initially, the cycloaddition of the azide anion to **1b** would lead to the tetrazolide anion **14b** through the transition structure **TS1** (ΔΔ*G* = +132.0 kJ mol^–1^). Alternatively, **1b** could transform through the less energetic **TS′1** (ΔΔ*G* = +120.7 kJ mol^–1^), in which only one bond between the C atom of the isocyanide and the terminal N atom of the azide anion is forming, thus leading to the azidoimidoyl anion **13b** (+110.5 kJ mol^–1^). However, this latter scenario is reversible and thermodynamically disfavored with respect to the formation of **14b**. Subsequently, **14b** loses molecular nitrogen, via the easily surmountable **TS2** (ΔΔ*G* = +53.9 kJ mol^–1^), to the very stable cyanamide anion **15b**. Two almost isoenergetic conformers were located for **15b**, the rotamer **15b′*Z*** being only 1.2 kJ mol^–1^ more stable than **15b′*E*** (−313.8 kJ mol^–1^). Each one of these rotamers is pre-oriented to undergo an intramolecular S_N_2 reaction by linking either the internal N atom (**15b′*E***) or the terminal N atom (**15b′*Z***) of the anionic NCN fragment to the methylenic carbon atom at the side-chain, with displacement of the azide anion, respectively, leading to **8b** or **4b**. Thus, **15b′*E*** would lead to the cyanamide **8b** (blue dotted lines) via **TS3** (ΔΔ*G* = +134.9 kJ mol^–1^) in what is the rate-determining step of the whole reaction path going from **1b** to **8b**. We also located the higher energetic **TS′3** (ΔΔ*G* = +181.8 kJ mol^–1^) converting **15b′*Z*** into the cyclic carbodiimide **4b** (red dotted lines), consequently discarding the putative routes leading to **8b** via **4b**.

The current computational study agrees well with the experimental observation of a long-lived intermediate (**LLI**) when the reaction was monitored by ^1^H NMR spectroscopy. In fact, the computed structures for **15b′*E*** or **15b′*Z***, which possess a negative charge delocalized along the aromatic ring, are in accordance with the shifts to lower frequencies experienced by the aromatic protons of **LLI**, detected in an early stage of the reaction (Scheme 3 and Figure 2).

## Conclusions

Herein, we have shown a novel reaction between the azide and isocyanide functions, when both are linked to an aryl-triazolyl moiety, triggered by the action of substoichiometric amounts of sodium azide. The result is an intramolecular cyclization leading to cyclic cyanamides, which could subsequently evolve to tetrazoles by a [3 + 2] cycloaddition between its cyano group and the azide anion, providing an excess of this latter reagent is present.

These processes constitute a new instance of the metal-free reactions between the azido and isocyanide functions, in this case as an approach to complex nitrogenated heterocycles. The uniqueness of this methodology lies in the catalytic role of the azide anion to promote the formation of cyanamide derivatives. Computational studies have corroborated our mechanistic proposal initiated by the addition of the azide anion to the isocyanide functionality. Moreover, the monitoring of the reaction by means of ^1^H NMR spectroscopy helped to postulate the respective cyanamide anions as the key intermediates of these processes. The chemical behavior of our azido-isocyanides has been also compared with that of one isomeric azido-cyanide which, in contrast, only cyclized through the well-known intramolecular [3 + 2] cycloaddition between its two functionalities. In our opinion, the present work contributes to a better understanding of the reactivity between azides and isocyanides in the absence of metals.

## Experimental Section

### General Methods

All reagents were purchased from commercial sources and used without further purification. HPLC grade solvents were nitrogen saturated and were dried and deoxygenated using an Innovative Technology Inc. Pure-Solv 400 Solvent Purification System. Column chromatography was carried out using silica gel (60 Å, 70–200 μm, SDS) as the stationary phase and TLC was performed on precoated silica gel on aluminum cards (0.25 mm thick, with fluorescent indicator 254 nm) and observed under UV light. All melting points were determined on a Kofler hot-plate melting point apparatus and are uncorrected. Fourier-transform infrared (FT-IR) spectra were recorded on a PerkinElmer Spectrum 65 or JASCO FT/IR-4700 spectrometers, and data are quoted in wavenumbers (cm^–1^). The intensities of the absorption bands are indicated as vs (very strong), s (strong), m (middle), and w (weak). NMR spectra were recorded on Bruker AVANCE 600 MHz or Bruker AVANCE 400 MHz instruments (for the latter, the operation frequency for ^1^H and ^13^C are 400.917 MHz and 100.82 MHz, respectively). ^1^H NMR chemical shifts are reported relative to Me_4_Si and were referenced via residual proton resonances of the corresponding deuterated solvent, whereas ^13^C NMR spectra are reported relative to Me_4_Si using the carbon signals of the deuterated solvent. Signals in the ^1^H and ^13^C NMR spectra of the synthesized compounds were assigned with the aid of DEPT-135. Structural assignments were made with additional information from gCOSY, NOESY, gHSQC, and gHMBC experiments. High-resolution mass spectra (HRMS) were recorded on Agilent HPLC 1200/MS TOF 6220 or Agilent HPLC 1290 Infinity II/MS Q-TOF 6550 mass spectrometers with ESI sources. The preparation of α-ketophosphorane **6**(23) and 1-azido-2-isocyanoarenes **5a–d**(19) was carried out by experimental procedures previously described in the literature. Azides are potentially explosive, and all reactions should be carried out behind blast shields.

### General Procedure for the Synthesis of 5-(Chloromethyl)-1-(2-isocyanoaryl)-1*H*-1,2,3-triazoles **7**

To a solution of the corresponding 2-azidoisocyanobenzene **5** (1.00 mmol, 1.0 equiv) in toluene (12 mL), the α-ketophosphorane **6** (0.353 g; 1.00 mmol, 1.0 equiv) was added. The reaction mixture was stirred at 80° C in a block heater for 8 h. After complexion of the reaction, the solvent was removed under reduced pressure. The crude product was purified by column chromatography using 1:1 AcOEt/hexane as the eluent.

#### 5-(Chloromethyl)-1-(2-isocyanophenyl)-1*H*-1,2,3-triazole (**7a**)

Yellow oil (120 mg, 55%); IR (solid, ATR, cm^–1^) ν: 2125 (m, N≡C), 1500 (m), 1445 (m), 1190 (m), 1121 (m), 724 (s), 698 (vs); ^1^H NMR (400 MHz, CDCl_3_, 298 K): δ 7.90 (t, *J* = 0.5 Hz, 1H), 7.70–7.63 (m, 3H), 7.60–7.57 (m, 1H), 4.58 (d, *J* = 0.5 Hz, 2H); ^13^C{^1^H} (100 MHz, CDCl_3_, 298 K): δ 171.5 (N≡C), 135.2 (C), 134.2 (CH), 131.9 (CH), 131.8 (C), 130.6 (CH), 129.0 (CH), 128.2 (CH), 123.8 (C), 32.0 (CH_2_); HRMS (ESI): calcd for C_10_H_8_ClN_4_ [M + H]^+^, 219.0432; found, 219.0432.

#### 5-(Chloromethyl)-1-(2-isocyano-3,5-dimethylphenyl)-1*H*-1,2,3-triazole (**7b**)

Colorless prism (244 mg, 99%); mp 100–102 °C; IR (nujol, cm^–1^) ν: 2129 (s, N≡C), 1601 (w), 1493 (m), 1471 (m), 1442 (m), 1235 (m), 1101 (m), 986 (m), 884 (m), 855 (m), 710 (vs), 652 (m), 601 (w); ^1^H NMR (400 MHz, CDCl_3_, 298 K): δ 7.88 (m, 1H), 7.34–7.33 (m, 1H), 7.20–7.19 (m, 1H), 4.56 (s, 2H), 2.50 (s, 3H), 2.44 (s, 3H); ^13^C{^1^H} NMR (100 MHz, CDCl_3_, 298 K): δ 172.1 (N≡C), 140.8 (C), 137.0 (C), 135.1 (C), 134.1 (CH), 133.6 (CH), 131.5 (C), 126.7 (CH), 121.1 (C), 32.1 (CH_2_), 21.3 (CH_3_), 18.9 (CH_3_); HRMS (ESI): calcd for C_12_H_12_ClN_4_ [M + H]^+^, 247.0745; found, 247.0753.

#### 5-(Chloromethyl)-1-(2-isocyano-3-methylphenyl)-1*H*-1,2,3-triazole (**7c**)

Yellow prisms (207 mg, 89%); mp 91–92 °C; IR (solid, ATR, cm^–1^) ν: 2130 (m, N≡C), 1491 (m), 1469 (w), 1442 (w), 1235 (m), 1106 (m), 979 (m), 853 (m), 793 (s), 728 (m), 713 (vs); ^1^H NMR (400 MHz, CDCl_3_, 298 K): δ 7.89 (s, 1H), 7.57–7.50 (m, 2H), 7.41–7.39 (m, 1H), 4.56 (s, 2H), 2.55 (s, 3H); ^13^C{^1^H} NMR (150 MHz, CDCl_3_, 298 K): δ 173.1 (N≡C), 137.5 (C), 135.1 (C), 134.1 (CH), 133.0 (CH), 131.8 (C), 129.8 (CH), 126.2 (CH), 123.8 (C), 32.0 (CH_2_), 19.0 (CH_3_); HRMS (ESI): calcd for C_11_H_10_ClN_4_ [M + H]^+^, 233.0589; found, 233.0591.

#### 1-(3-Chloro-2-isocyano-5-methylphenyl)-5-(chloromethyl)-1*H*-1,2,3-triazole (**7d**)

Yellow oil (254 mg, 95%); ^1^H NMR (400 MHz, CDCl_3_, 298 K): δ 7.88 (s, 1H), 7.57–7.56 (m, 1H), 7.31–7.30 (m, 1H), 4.58 (s, 2H), 2.49 (m, 3H), ^13^C{^1^H} NMR (100 MHz, CDCl_3_, 298 K): δ 175.5 (N≡C), 142.1 (C), 135.2 (C), 134.2 (CH), 132.80 (CH), 132.78 (C), 132.3 (C), 127.7 (CH), 120.5 (C), 31.9 (CH_2_), 21.4 (CH_3_); HRMS (ESI): calcd for C_11_H_9_Cl_2_N_4_ [M + H]^+^, 267.0199; found, 267.0190.

### General Procedure for the Synthesis of 5-(Azidomethyl)-1-(2-isocyanoaryl)-1*H*-1,2,3-triazoles **1**

To a solution of the corresponding 5-(chloromethyl)-1-(2-isocyanoaryl)-1*H*-1,2,3-triazole **7** (1.00 mmol, 1.0 equiv) in DMF (16 mL) sodium azide (0.200 g; 3.00 mmol, 3.0 equiv) was added. The reaction mixture was stirred for 6 h at room temperature. After the complexion of the reaction, water (40 mL) was added and the reaction mixture extracted with AcOEt (3 × 25 mL). The organic layer was washed with brine (25 mL) and dried over anhydrous MgSO_4_. After removal of the solvent under reduced pressure, the residue was purified by column chromatography and eluted with 1:1 AcOEt/hexane to afford the desired product.

#### 5-(Azidomethyl)-1-(2-isocyanophenyl)-1*H*-1,2,3-triazole (**1a**)

Yellow oil (118 mg, 60%); ^1^H NMR (400 MHz, CDCl_3_, 298 K): δ 7.90 (s, 1H), 7.70–7.62 (m, 3H), 7.57–7.55 (m, 1H), 4.43 (s, 2H); ^13^C{^1^H} NMR (100 MHz, CDCl_3_, 298 K): δ 171.6 (N≡C), 133.9 (CH), 133.6 (C), 131.9 (CH + C), 130.7 (CH), 128.8 (CH), 128.2 (CH), 123.7 (C), 42.9 (CH_2_); HRMS (ESI): calcd for C_10_H_8_N_7_ [M + H]^+^, 226.0838; found, 226.0844.

#### 5-(Azidomethyl)-1-(2-isocyano-3,5-dimethylphenyl)-1*H*-1,2,3-triazole (**1b**)

Colorless prisms (236 mg, 93%); mp 75–77 °C; IR (nujol, cm^–1^) ν: 2126 (vs, N≡C), 2101 (m, N_3_), 2077 (m, N_3_), 1237 (vs), 1215 (m), 1108 (m), 977 (m), 880 (s), 833 (m), 800 (m); ^1^H NMR (400 MHz, CDCl_3_, 298 K): δ 7.86 (m, 1H), 7.34–7.33 (m, 1H), 7.16–7.15 (m, 1H), 4.40 (s, 2H), 2.49 (s, 3H), 2.43 (s, 3H); ^13^C{^1^H} NMR (150 MHz, CDCl_3_, 298 K): δ 172.2 (N≡C), 140.9 (C), 137.0 (C), 133.7 (CH), 133.53 (CH), 133.51 (C), 131.6 (C), 126.5 (CH), 121.0 (C), 42.9 (CH_2_), 21.3 (CH_3_), 18.8 (CH_3_); HRMS (ESI): calcd for C_12_H_12_N_7_ [M + H]^+^, 254.1149; found, 254.1145.

#### 5-(Azidomethyl)-1-(2-isocyano-3-methylphenyl)-1*H*-1,2,3-triazole (**1c**)

Yellow prisms (218 mg, 91%); mp 60–62 °C; IR (nujol, cm^–1^) ν: 2125 (m, N≡C), 2090 (s, N_3_), 1493 (m), 1350 (m), 1256 (m), 1233 (m), 1097 (m), 980 (m), 870 (m), 804 (vs), 733 (m), 687 (w); ^1^H NMR (400 MHz, CDCl_3_, 298 K): δ 7.88 (s, 1H), 7.56–7.50 (m, 2H), 7.36 (dd, *J* = 7.3, 1.7 Hz, 1H), 4.40 (s, 2H), 2.54 (s, 3H); ^13^C{^1^H} NMR (150 MHz, CDCl_3_, 298 K): δ 173.0 (N≡C), 137.5 (C), 133.8 (CH), 133.5 (C), 133.0 (CH), 131.9 (C), 129.8 (CH), 126.0 (CH), 123.7 (C), 42.8 (CH_2_), 19.0 (CH_3_); HRMS (ESI): calcd for C_11_H_10_N_7_ [M + H]^+^, 240.0992; found, 240.0986.

#### 5-(Azidomethyl)-1-(3-chloro-2-isocyano-5-methylphenyl)-1*H*-1,2,3-triazole (**1d**)

Colorless prisms (142 mg, 52%); mp 87–89 °C; IR (solid, ATR, cm^–1^) ν: 2125 (vs, N≡C), 2102 (m, N_3_), 2076 (m, N_3_), 1488 (m), 1463 (w), 1446 (m), 1238 (vs), 1206 (m), 1115 (m), 1089 (m), 1069 (w), 964 (m), 880 (s), 831 (m), 801 (m), 758 (m), 724 (m) 666 (m), 648 (m), 605 (w); ^1^H NMR (400 MHz, CDCl_3_, 298 K): δ 7.88 (s, 1H), 7.56 (m, 1H), 7.28 (m, 1H), 4.44 (s, 2H), 2.49 (s, 3H); ^13^C{^1^H} NMR (100 MHz, CDCl_3_, 298 K): δ 175.5 (N≡C), 142.2 (C), 134.0 (CH), 133.6 (C), 132.9 (C), 132.8 (CH), 132.4 (C), 127.6 (CH), 120.4 (C), 42.9 (CH_2_), 21.4 (CH_3_); HRMS (ESI): calcd for C_11_H_9_ClN_7_ [M + H]^+^, 274.0602; found, 274.0599.

### General Procedure for the Synthesis of [1,2,3]triazolo[1,5-*a*]quinoxaline-5(4*H*)-carbonitriles **8**

Sodium azide (0.033 g; 0.50 mmol, 0.5 equiv) was added to a solution of the corresponding 5-azidomethyl-1-(2-isocyanophenyl)-1*H*-1,2,3-triazole **1** (1.00 mmol, 1.0 equiv) in DMF (11 mL). The reaction mixture was stirred at 80 °C in a block heater for 48 h. After the complexion of the reaction, water (20 mL) was added, and the mixture was extracted with AcOEt (3 × 15 mL). The organic layer was then washed with water (15 mL) and dried over MgSO_4_. After removal of the solvent under reduced pressure, the residue was purified by column chromatography eluting with 1:1 AcOEt/hexane.

#### [1,2,3]Triazolo[1,5-*a*]quinoxaline-5(4*H*)-carbonitrile (**8a**)

Colorless prisms (61 mg, 31%); mp 175–176 °C; ^1^H NMR (400 MHz, CDCl_3_, 298 K): δ 8.19 (dd, *J* = 8.4, 2.0 Hz, 1H), 7.70 (s, 1H), 7.44–7.38 (m, 2H), 7.31 (ddd, *J* = 8.2, 6.7, 1.7 Hz, 1H), 5.11 (d, *J* = 0.5 Hz, 2H); ^13^C{^1^H} NMR (100 MHz, CDCl_3_, 298 K): δ 130.1 (CH), 129.2 (CH), 126.7 (C), 125.6 (CH), 125.5 (C), 123.9 (C), 117.7 (CH), 117.0 (CH), 111.0 (C), 44.5 (CH_2_); HRMS (ESI): calcd for C_10_H_8_N_5_ [M + H]^+^, 198.0780; found, 198.0779.

#### 6,8-Dimethyl-[1,2,3]triazolo[1,5-*a*]quinoxaline-5(4*H*)-carbonitrile (**8b**)

Colorless prisms (151 mg, 67%); mp 190–192 °C; IR (solid, ATR, cm^–1^) ν: 2920 (m), 2852 (w), 2220 (m, CN), 1482 (vs), 1446 (m), 1369 (m), 1226 (m), 1108 (m), 985 (vs), 859 (vs), 695 (m); ^1^H NMR (400 MHz, CDCl_3_, 298 K): δ 7.89 (s, 1H), 7.71 (s, 1H), 7.09–7.08 (m, 1H), 4.86 (d, *J* = 0.7 Hz, 2H), 2.49 (s, 3H), 2.42 (s, 3H); ^13^C{^1^H} NMR (100 MHz, CDCl_3_, 298 K): δ 138.4 (C), 131.9 (C), 131.8 (CH), 129.8 (CH), 127.6 (C), 127.4 (C), 122.6 (C), 116.2 (CH), 112.9 (C), 45.7 (CH_2_), 21.2 (CH_3_), 17.8 (CH_3_); HRMS (ESI): calcd for C_12_H_12_N_5_ [M + H]^+^, 226.1087; found, 226.1080.

#### 6-Methyl-[1,2,3]triazolo[1,5-*a*]quinoxaline-5(4*H*)-carbonitrile (**8c**)

Colorless prisms (87 mg, 41%); mp 149–150 °C; ^1^H NMR (400 MHz, CDCl_3_, 298 K): δ 8.08–8.06 (m, 1H), 7.72 (t, *J* = 1.5 Hz, 1H), 7.37 (t, *J* = 7.8 Hz, 1H), 7.28 (dm, *J* = 7.8 Hz, 1H), 4.90 (d, *J* = 0.8 Hz, 2H), 2.54 (s, 3H); ^13^C{^1^H} NMR (100 MHz, CDCl_3_, 298 K): δ 132.2 (C), 131.2 (CH), 129.8 (CH), 127.84 (C), 127.82 (CH), 127.4 (C), 125.0 (C), 115.8 (CH), 112.7 (C), 45.7 (CH_2_), 18.0 (CH_3_); HRMS (ESI): calcd for C_11_H_10_N_5_ [M + H]^+^, 212.0931; found, 212.0937.

#### 6-Chloro-8-methyl-[1,2,3]triazolo[1,5-*a*]quinoxaline-5(4*H*)-carbonitrile (**8d**)

Colorless prisms (61 mg, 25%); mp 145–147 °C; ^1^H NMR (400 MHz, CDCl_3_, 298 K): δ 7.98–7.97 (m, 1H), 7.73 (t, *J* = 0.7 Hz, 1H), 7.31–7.30 (m, 1H), 4.93 (d, *J* = 0.8 Hz, 2H), 2.46 (t, *J* = 0.7 Hz, 3H); ^13^C{^1^H} NMR (100 MHz, CDCl_3_, 298 K): δ 139.5 (C), 130.6 (CH), 130.0 (CH), 128.5 (C), 127.6 (C), 127.5 (C), 121.5 (C), 117.0 (CH), 111.7 (C), 46.1 (CH_2_), 21.2 (CH_3_); HRMS (ESI): calcd for C_11_H_9_ClN_5_ [M + H]^+^, 246.0541; found, 246.0541.

### General Procedure for the Synthesis of 5-(2*H*-tetrazol-5-yl)-4,5-dihydro-[1,2,3]triazolo[1,5-*a*]quinoxalines **9**

To a solution of the corresponding 5-azidomethyl-1-(2-isocyanophenyl)-1*H*-1,2,3-triazole **1** (1.00 mmol, 1.0 equiv) in DMF (8 mL) sodium azide (0.130 g; 2 mmol, 2.0 equiv) was added. The reaction mixture was stirred at 80 °C in a block heater for 7 days. After complexion of the reaction, the mixture was acidified to pH = 3–4 with an aqueous solution of 1 M HCl. Then, water (15 mL) was added and the aqueous phase extracted with AcOEt (3 × 20 mL). The organic extracts were dried with MgSO_4_. After removal of the solvent under reduced pressure, the residue was purified by column chromatography sequentially eluting with 9:1 chloroform/acetone, 7:3 chloroform/acetone and, finally, 4:1 chloroform/methanol.

#### 5-(2*H*-Tetrazol-5-yl)-4,5-dihydro-[1,2,3]triazolo[1,5-*a*]quinoxaline (**9a**)

Colorless prisms (94 mg, 39%); mp > 300 °C; ^1^H NMR (400 MHz, DMSO-*d*_6_, 298 K): δ 8.04 (dd, *J* = 8.0,1.4 Hz, 1H), 7.93 (dd, *J* = 8.4, 1.0 Hz, 1H), 7.83 (s, 1H), 7.34 (ddd, *J* = 8.6, 7.3, 1.4 Hz, 1H), 7.15 (td, *J* = 7.7, 1.2 Hz, 1H), 5.25 (s, 2H); ^13^C{^1^H} NMR (100 MHz, DMSO-*d*_6_, 298 K): δ 161.4 (C), 132.0 (C), 129.7 (CH), 129.6 (C), 128.3 (CH), 123.6 (C), 121.8 (CH), 118.8 (CH), 116.4 (CH), 43.1 (CH_2_); HRMS (ESI): calcd for C_10_H_7_N_8_ [M – H]^−^, 239.0799; found, 239.0805.

#### 6,8-Dimethyl-5-(2*H*-tetrazol-5-yl)-4,5-dihydro-[1,2,3]triazolo[1,5-*a*]quinoxaline (**9b**)

Colorless prisms (215 mg, 80%); mp 198–200 °C; IR (solid, ATR, cm^–1^) ν: 3208 (m, NH), 2922 (m), 1574 (m), 1478 (vs), 1437 (m), 1407 (m), 1223 (m), 1093 (m), 988 (m), 853 (m), 739 (m), 691 (m), 661 (s), 634 (s); ^1^H NMR (400 MHz, DMSO-*d*_6_, 298 K): δ 7.75 (s, 1H), 7.71 (m, 1H), 7.13 (m, 1H), 5.11 (s, 2H), 2.39 (s, 3H), 2.12 (s, 3H); ^13^C{^1^H} NMR (100 MHz, DMSO-*d*_6_, 298 K): δ 163.6 (C), 135.1 (2×C), 131.2 (C), 130.6 (CH), 129.5 (CH), 129.4 (C), 128.5 (C), 114.5 (CH), 44.3 (CH_2_), 20.7 (CH_3_), 18.1 (CH_3_); HRMS (ESI): calcd for C_12_H_13_N_8_ [M + H]^+^, 269.1258; found, 269.1268.

#### 6-Methyl-5-(2*H*-tetrazol-5-yl)-4,5-dihydro-[1,2,3]triazolo[1,5-*a*]quinoxaline (**9c**)

Colorless prisms (206 mg, 81%); mp 252–254 °C; ^1^H NMR (400 MHz, DMSO-*d*_6_, 298 K): δ 16.0 (br s, 1H), 7.97 (d, *J* = 7.7 Hz, 1H), 7.86 (s, 1H), 7.49–7.41 (m, 2H), 5.27 (s, 2H), 2.18 (s, 3H); ^13^C{^1^H} NMR (100 MHz, DMSO-*d*_6_, 298 K): δ 160.5 (C), 135.0 (C), 130.8 (C), 130.5 (CH), 130.0 (CH), 129.2 (C), 129.0 (C), 127.3 (CH), 114.8 (CH), 44.1 (CH_2_), 17.7 (CH_3_); HRMS (ESI): calcd for C_11_H_11_N_8_ [M + H]^+^, 255.1107; found, 255.1108.

#### 6-Chloro-8-methyl-5-(2*H*-tetrazol-5-yl)-4,5-dihydro-[1,2,3]triazolo[1,5-a]quinoxaline (**9d**)

Colorless prisms (165 mg, 57%); mp > 300 °C; ^1^H NMR (400 MHz, DMSO-*d*_6_, 298 K): δ 7.92 (m, 1H), 7.81 (s, 1H), 7.46–7.45 (m, 1H), 5.23 (s, 2H), 2.45 (s, 3H); ^13^C{^1^H} NMR (100 MHz, DMSO-*d*_6_, 298 K): δ 161.8 (C), 137.7 (C), 130.9 (C), 129.9 (CH), 129.8 (C), 129.7 (C), 129.6 (CH), 127.0 (C), 116.1 (CH), 44.7 (CH_2_), 20.4 (CH_3_); HRMS (ESI): calcd for C_11_H_10_ClN_8_ [M + H]^+^, 289.0711; found, 289.0709.

### Synthesis of 2-[5-(chloromethyl)-1*H*-1,2,3-triazol-1-yl]benzonitrile (**10**)

To a solution of 2-azidobenzonitrile (1.0 g, 7.7 mmol, 1.0 equiv) in toluene (40 mL) under a nitrogen-gas atmosphere was added α-ketophosphorane (**6**) (2.71 g, 7.7 mmol, 1.0 equiv) and the mixture was heated at 80 °C with stirring in a block heater for 15 h. After this time, the solvent was removed under reduced pressure to give a residue which was purified by column chromatography eluting with 7:3 AcOEt/hexane to give the title compound as a white solid after treatment with CH_2_Cl_2_/Et_2_O (1.53 g, 91%); mp 96–98 °C; FT-IR (solid, ATR, cm^–1^) ν: 2235 (w, CN), 1598 (w), 1504 (m), 1451 (m), 1258 (w), 1234 (m), 1168 (w), 1091 (m), 974 (m), 854 (m), 774 (vs), 721 (s), 698 (m), 670 (m), 649 (m); ^1^H NMR (600 MHz, CDCl_3_, 298 K): δ 7.89 (dd, *J* = 7.8, 1.3 Hz, 1H), 7.87 (s, 1H), 7.84 (td, *J* = 7.9, 1.5 Hz, 1H), 7.73 (td, *J* = 7.8, 1.1 Hz, 1H), 7.64 (dd, *J* = 8.0, 0.8 Hz, 1H), 4.60 (s, 2H, CH_2_); ^13^C{^1^H} NMR (150 MHz, CDCl_3_, 298 K): δ 137.2 (C), 135.1 (C), 134.3 (CH), 134.2 (CH), 134.1 (CH), 131.2 (CH), 128.2 (CH), 114.7 (CN), 111.3 (C), 32.0 (CH_2_); HRMS (ESI): calcd for C_10_H_8_ClN_4_ [M + H]^+^, 219.0432; found, 219.0436.

### Synthesis of 2-[5-(Azidomethyl)-1*H*-1,2,3-triazol-1-yl]benzonitrile (**11**)

A mixture of 2-[5-(chloromethyl)-1*H*-1,2,3-triazol-1-yl]benzonitrile (**10**) (1.0 g, 4.6 mmol, 1.0 equiv) and sodium azide (0.89 g, 13.7 mmol, 3.0 equiv) in DMF (50 mL) was stirred at room temperature for 24 h. Water (100 mL) was added and the reaction mixture was extracted with AcOEt (3 × 50 mL). The organic layer was washed with brine (3 × 50 mL), dried over anhydrous MgSO_4_, and the solvent evaporated under reduced pressure. The obtained residue was purified by column chromatography eluting with 7:3 AcOEt/hexane to give the title compound as a yellow oil (0.85 g, 82%); FT-IR (solid, ATR, cm^–1^) ν: 2234 (w, CN), 2098 (s, N_3_), 1597 (w), 1502 (m), 1457 (m), 1345 (w), 1253 (m), 1234 (m), 1202 (w), 1114 (w), 1082 (m), 976 (m), 960 (m), 880 (w), 840 (w), 768 (vs), 700 (w), 650 (w); ^1^H NMR (400 MHz, CDCl_3_, 298 K): δ 7.90–7.82 (m, 3H), 7.72 (td, *J* = 7.7, 1.1 Hz, 1H), 7.60 (d, *J* = 8.0 Hz, 1H), 4.45 (s, 2H); ^13^C{^1^H} NMR (100 MHz, CDCl_3_, 298 K): δ 137.3 (C), 134.2 (CH), 134.10 (CH), 134.07 (CH), 133.5 (C), 131.2 (CH), 128.1 (CH), 114.7 (CN), 111.1 (C), 42.7 (CH_2_); HRMS (ESI): calcd for C_10_H_8_N_7_ [M + H]^+^, 226.0836; found, 226.0838.

### Synthesis of 9*H*-Benzo[*f*]tetrazolo[1,5-*d*][1,2,3]triazolo[1,5-*a*][1,4]diazepine (**12**)

A solution of 2-[5-(azidomethyl)-1*H*-1,2,3-triazol-1-yl]benzonitrile (**11**) (1.0 g, 4.4 mmol) in DMF (100 mL) was heated at 140 °C in a block heater for 48 h. After warming to room temperature, the mixture was poured into iced water (150 mL). The resultant solid was filtered, dried under reduced pressure, and crystallized from ethanol to give the title compound as white prisms (0.66 g, 66%); mp 262–264 °C; IR (solid, ATR, cm^–1^) ν: 1614 (w), 1485 (m), 1446 (m), 1409 (w), 1264 (m), 1237 (w), 1146 (m), 1163 (w), 1096 (m), 1052 (w), 974 (m), 854 (m), 795 (m), 764 (vs), 742 (s), 695 (w), 641 (m); ^1^H NMR (400 MHz, DMSO-*d*_6_, 298 K): δ 8.27–8.23 (m, 2H), 8.05 (s, 1H), 7.93 (t, *J* = 7.8 Hz, 1H), 7.80 (t, *J* = 7.6 Hz, 1H), 6.17 (s, 2H, CH_2_); ^13^C{^1^H} NMR (100 MHz, DMSO-*d*_6_, 298 K): δ 151.9 (C), 133.6 (C), 133.5 (CH), 133.1 (CH), 132.5 (C), 130.4 (CH), 129.7 (CH), 123.9 (CH), 114.9 (C), 39.5 (CH_2_); HRMS (ESI): calcd for C_10_H_8_N_7_ [M + H]^+^, 226.0836; found, 226.0837.

## Data Availability

The data underlying this study are available in the published article and its Supporting Information.

## Supporting Information Available

Crystal data, data collection, and structure refinement for **8b**; X-ray ORTEP figures; ^1^H NMR monitoring of the reaction of 5-azidomethyltriazole **1b** with NaN_3_ in DMF-*d*_7_; computed structures and theoretical ^13^C NMR chemical shifts of both N-1 and N-2 tautomeric forms of **9b**; NMR spectra of all new synthesized compounds; and computational methods and alternative mechanism initiated by an intramolecular [3 + 1] cycloaddition of the azido-isocyanide **1b** (PDF)
