# Supplementary material for: Intramolecular Cyclization of Azido-Isocyanides Triggered by the Azide Anion: An Experimental and Computational Study
Source: J Org Chem. 2023 Jun 20;88(13):8658–68. doi: 10.1021/acs.joc.3c00558 (PMC10861138; doi:10.1021/acs.joc.3c00558)
Supplement: Supplementary file 1 — jo3c00558_si_001.pdf [file jo3c00558_si_001.pdf]

Supporting Information

Intramolecular Cyclization of Azido-Isocyanides Triggered by the Azide Anion: An Experimental and Computational Study

Mateo Alajarin,[a] Guillermo Cutillas-Font,[a] Carmen Lopez-Leonardo,[a] Raul-Angel Orenes,[b] Marta Marin-Luna,[a] Aurelia Pastor[a]*

[a] Departamento de Química Orgánica, Facultad de Química, Regional Campus of International Excellence “Campus Mare Nostrum”, Universidad de Murcia, E-30100, Murcia, Spain; [b] ACTI, Universidad de Murcia, E-30100, Murcia, Spain

*E-mail: aureliap@um.es

**Table of Contents:**	
1. Crystal data, data collection and structure refinement for **8b**	S1-S2
2. ^{1}H-NMR monitoring of the reaction of 5-azidomethyltriazole **1b** with NaN_3 in DMF-d_7 at 80 ºC	S3-S8
3. Computed structures of N-1 and N-2 tautomeric forms of **9b**. Theoretical ^{1}H and ^{13}C NMR chemical shifts	S9-S10
4. ^{1}H and ^{13}C{^{1}H} NMR spectra of all new synthesized compounds (HMBC, HSQC, COSY and NOESY of selected compounds are also included)	S11-S50
5. Computational Study	S51-S58
6. References	S59

1. Crystal data, data collection and structure refinement for 8b

Single crystals of **8b** suitable for X-ray diffraction were obtained by liquid diffusion ($CHCl_3$/pentane). X-ray data were collected on a Bruker D8 Venture diffractometer with a Photon 100 detector (Cu-Kα radiation).

Table S1. Crystal data, data collection and structure refinement for **8b**.

Identification code	GCF_34F2r	
Empirical formula	C12 H11 N5	
Formula weight	225.26	
Temperature	100(2) K	
Wavelength	1.54178 Å	
Crystal system	Orthorhombic	
Space group	$P2_12_12_1$	
Unit cell dimensions	a = 5.5941(5) Å	α= 90°.
	b = 9.1862(8) Å	β= 90°.
	c = 21.1603(18) Å	γ = 90°.
Volume	1087.40(16) $Å^3$	
Z	4	
Density (calculated)	1.376 Mg/m^3	
Absorption coefficient	0.718 mm^{-1}	
F(000)	472	
Crystal size	0.330 x 0.120 x 0.060 mm^3	
Theta range for data collection	4.178 to 72.094°.	
Index ranges	-6<=h<=6, -11<=k<=11, -26<=l<=26	
Reflections collected	18032	
Independent reflections	2140 [R(int) = 0.0254]	
Completeness to theta = 67.679°	99.9 %	
Absorption correction	Semi-empirical from equivalents	
Max. and min. transmission	0.7536 and 0.6891	
Refinement method	Full-matrix least-squares on F^2	
Data / restraints / parameters	2140 / 0 / 156	
Goodness-of-fit on F^2	1.040	
Final R indices [I>2sigma(I)]	R1 = 0.0280, wR2 = 0.0751	
R indices (all data)	R1 = 0.0285, wR2 = 0.0758	
Absolute structure parameter	0.11(8)	
Extinction coefficient	n/a	
Largest diff. peak and hole	0.154 and -0.241 e.$Å^{-3}$	

Table S2. Hydrogen bonds for **8b**: [Å and °].

D-H...A	d(D-H)	d(H...A)	d(D...A)	<(DHA)
C7-H7A…N4#1	0.990	2.359	3.184	140.33

Symmetry transformations used to generate equivalent atoms:

#1 -x+3, y+1/2, -z+1/2

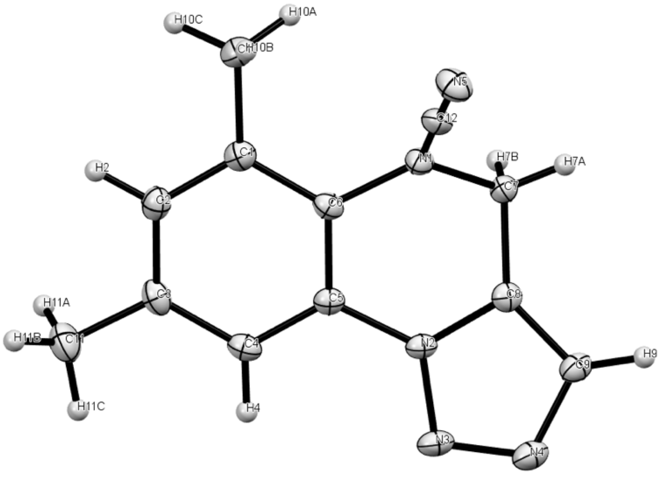

Figure S1. Molecular structure of **8b** with thermal ellipsoids drawn at 50% probability.

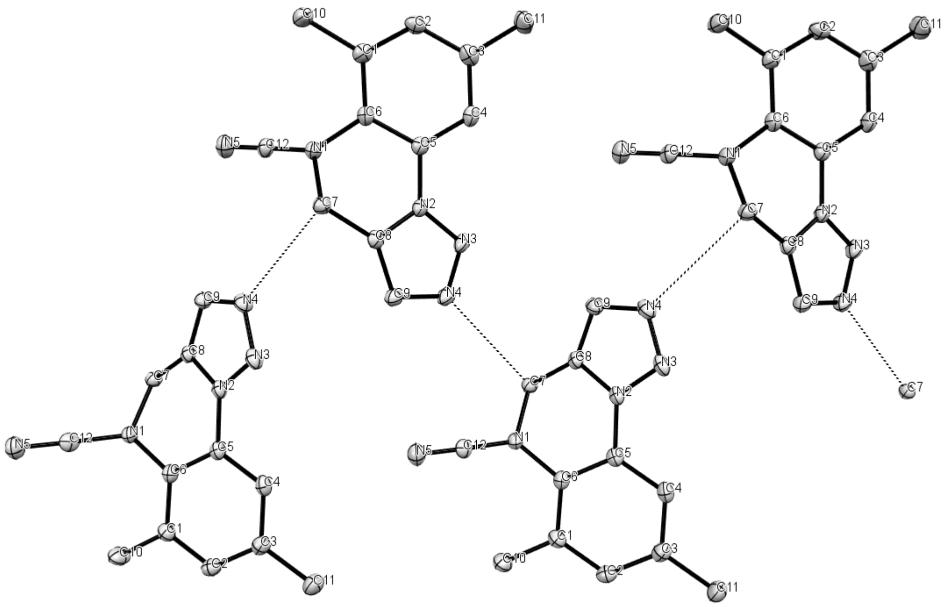

Figure S2. Hydrogen-bond network for the crystal structure of **8b**.

2. ^{1}H-NMR monitoring of the reaction of 5-azidomethyltriazole 1b with NaN_3 in DMF-d_7 at 80 ºC

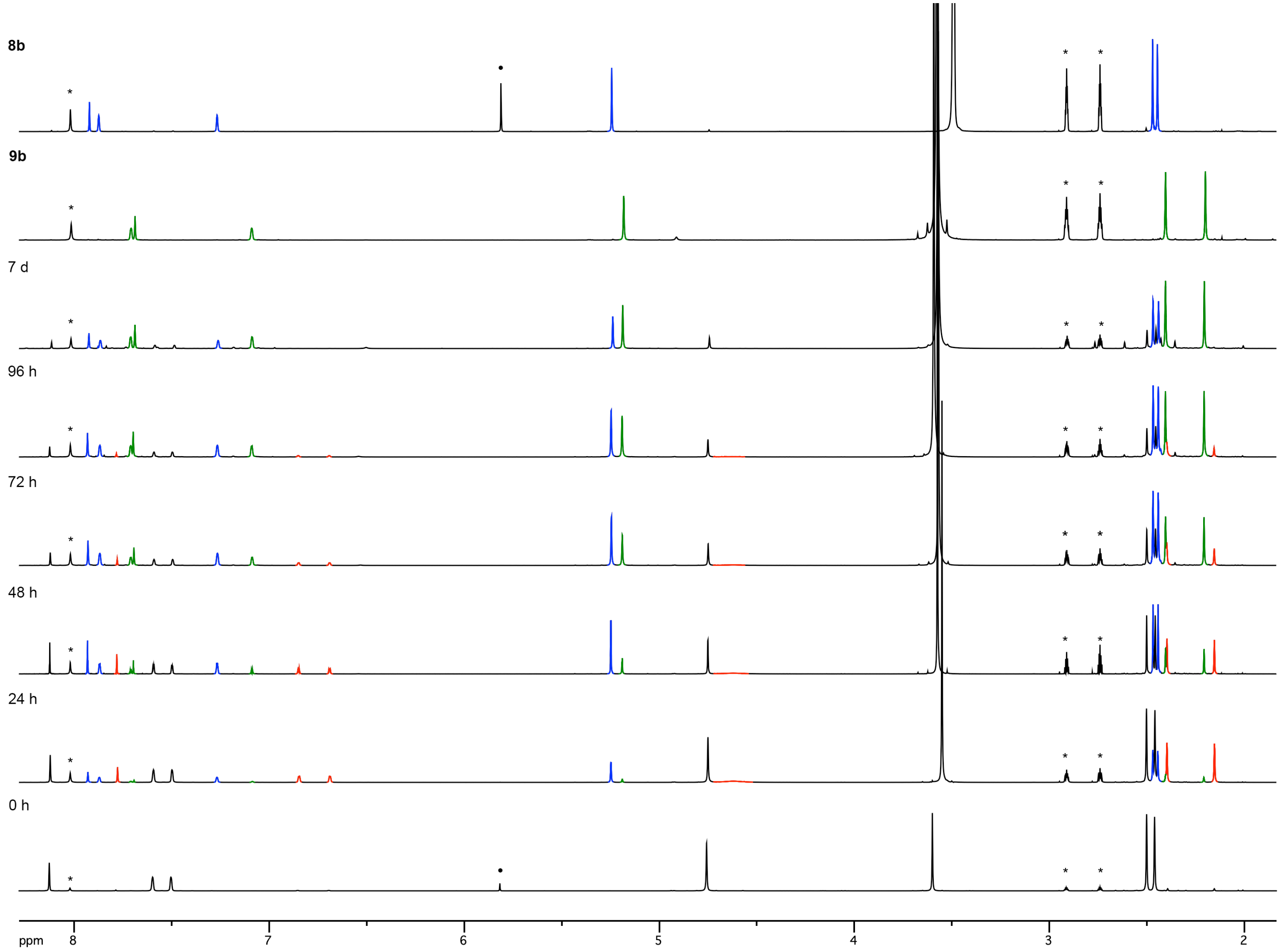

Figure S3a. ^{1}H-NMR (400 MHz) monitoring of the reaction of 5-azidomethyltriazole **1b** (black resonances) with **NaN_3 (0.5 equiv)** in DMF-d_7 at 80 ºC. Color code: **LLI** (red), **8b** (blue) and **9b** (green). (*) Residual solvent peaks and (•) dichloromethane.

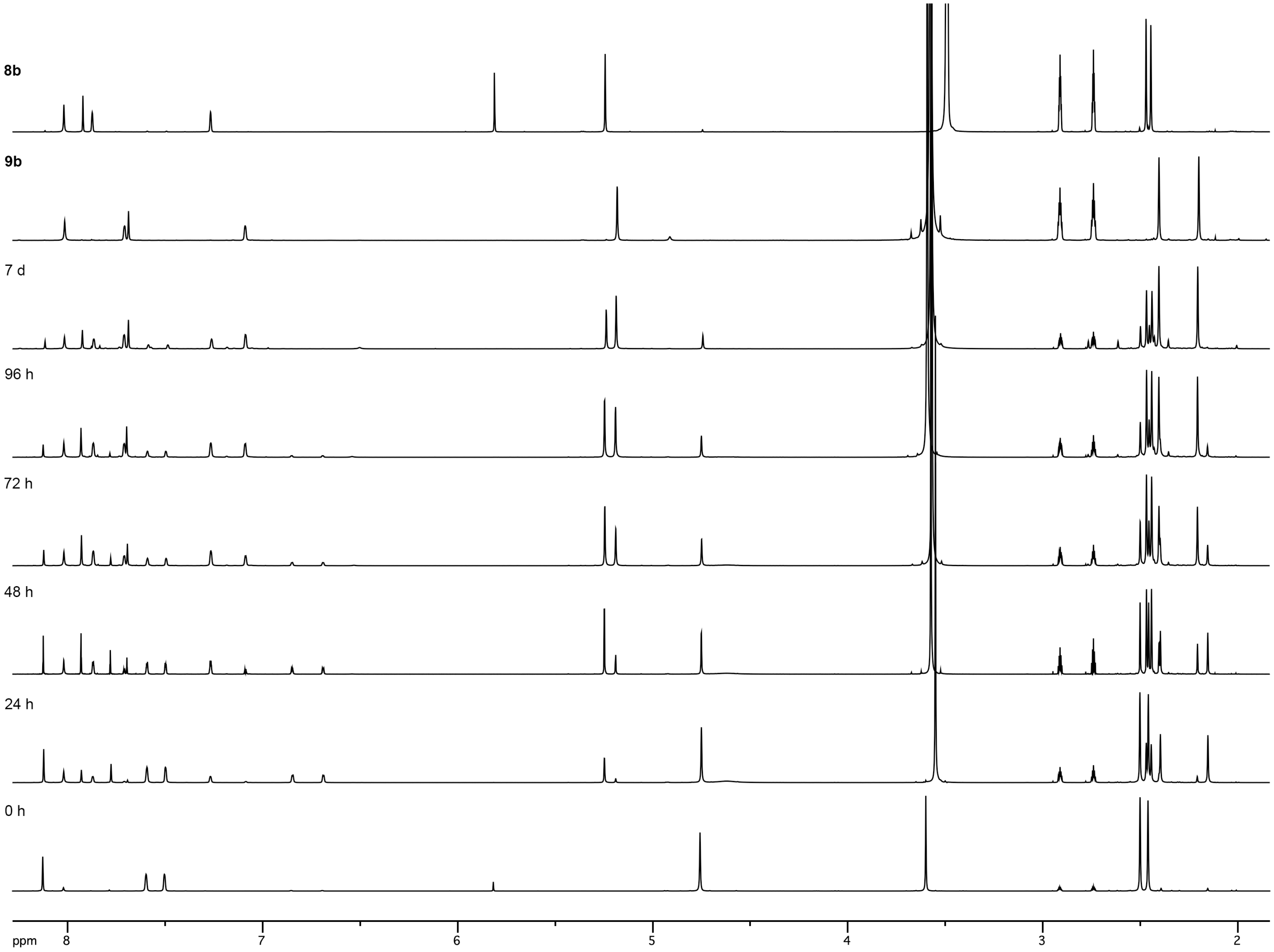

Figure S3b. ^{1}H-NMR (400 MHz) monitoring of the reaction of 5-azidomethyltriazole **1b** with **NaN_3 (0.5 equiv)** in DMF-d_7 at 80 ºC.

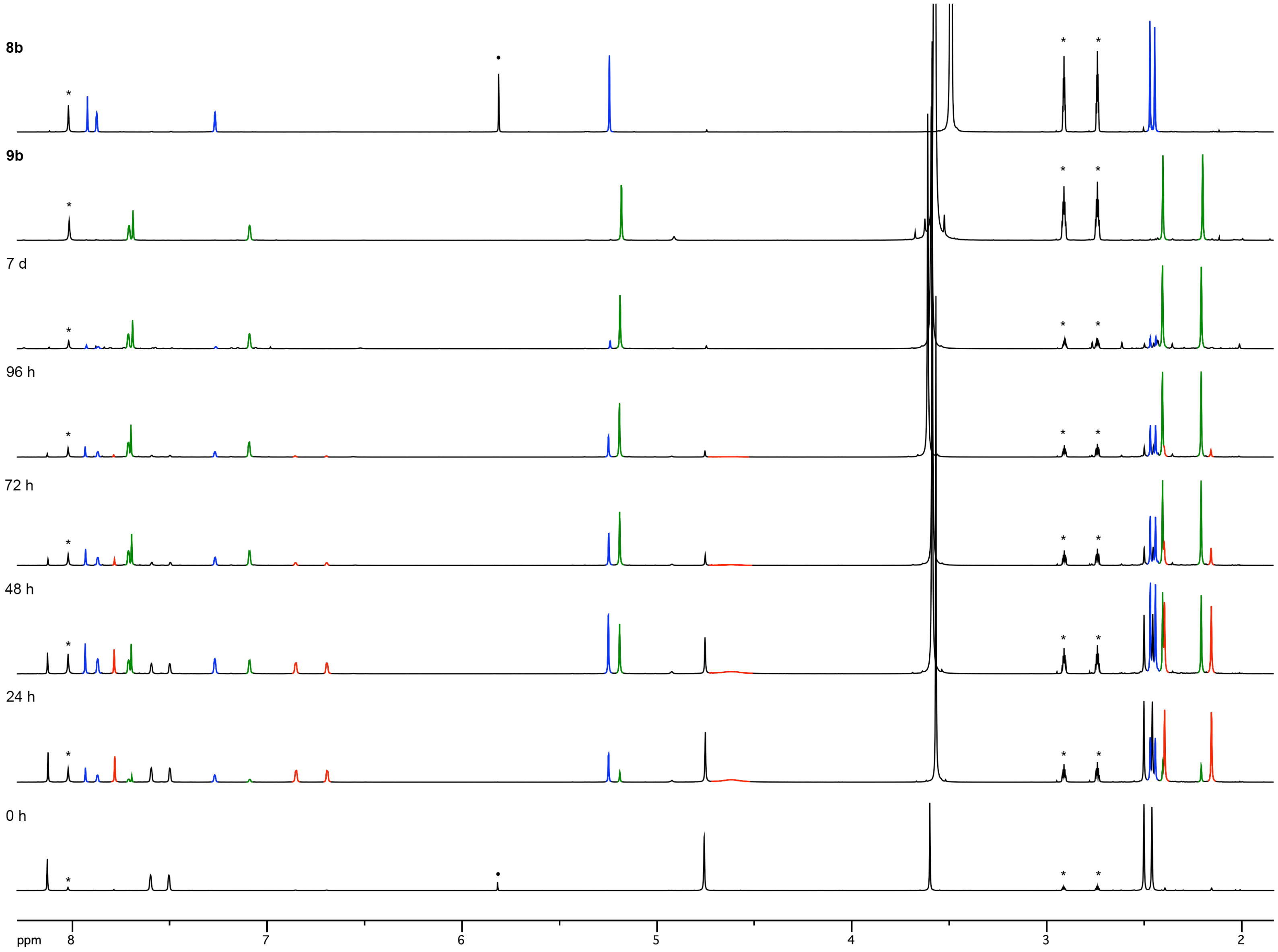

Figure S4a. ^{1}H-NMR (400 MHz) monitoring of the reaction of 5-azidomethyltriazole **1b** (black resonances) with **NaN_3 (1.0 equiv)** in DMF-d_7 at 80 ºC. Color code: **LLI** (red), **8b** (blue) and **9b** (green). (*) Residual solvent peaks and (•) dichloromethane.

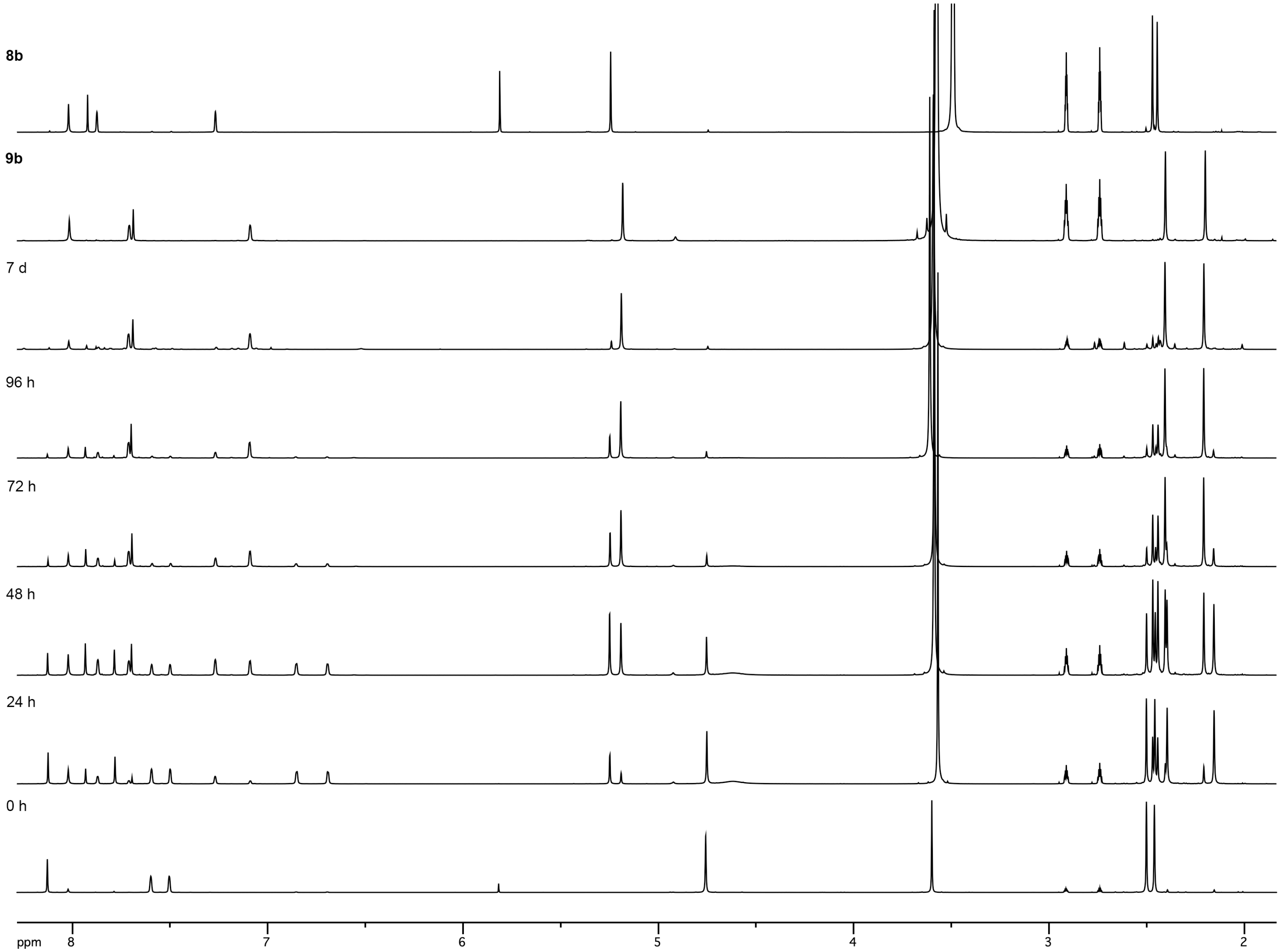

Figure S4b. ^{1}H-NMR (400 MHz) monitoring of the reaction of 5-azidomethyltriazole **1b** with **NaN$_3$ (1.0 equiv)** in DMF-d_7 at 80 ºC.

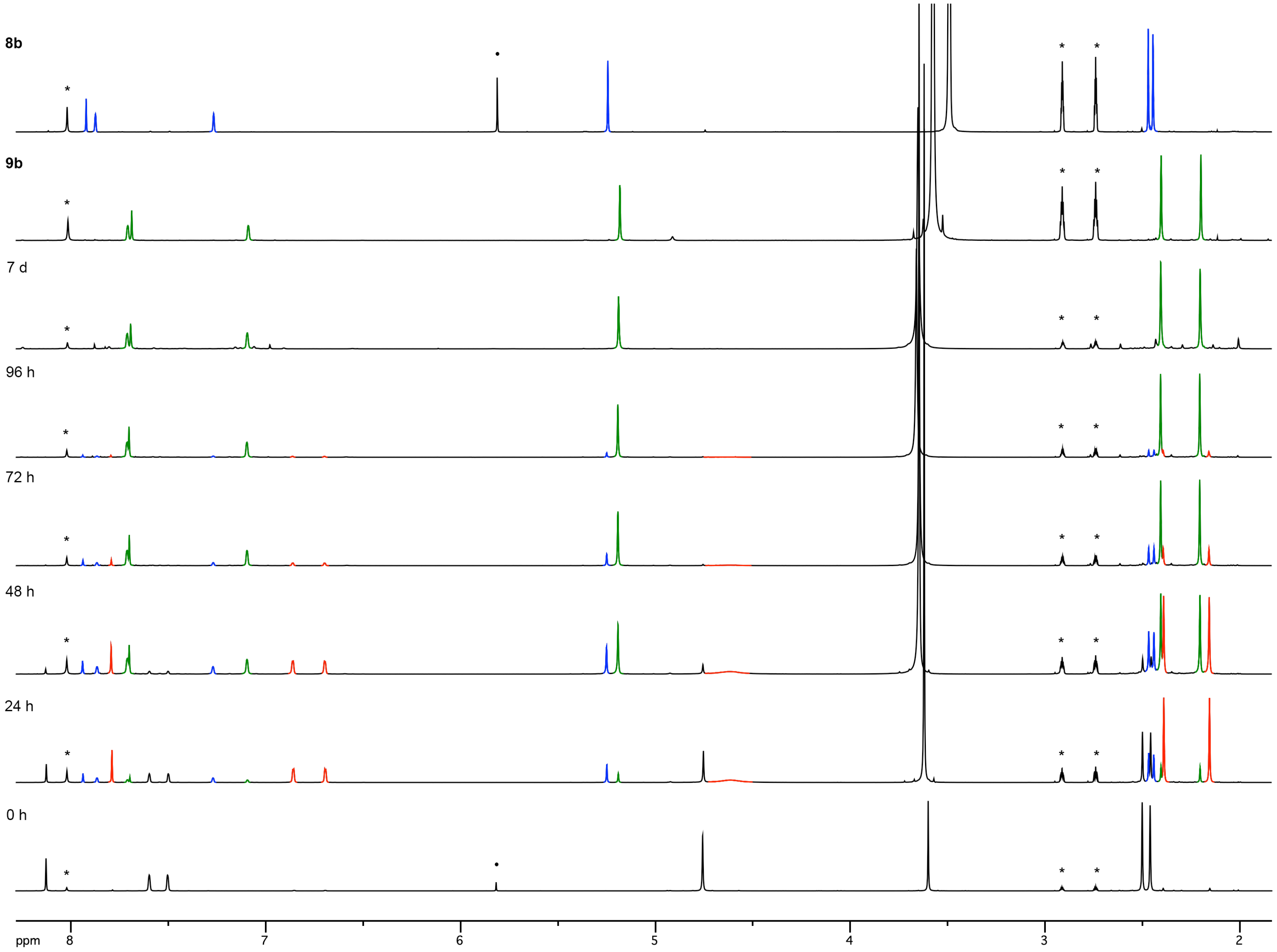

Figure S5a. ^{1}H-NMR (400 MHz) monitoring of the reaction of 5-azidomethyltriazole **1b** (black resonances) with **NaN_3 (1.5 equiv)** in DMF-d_7 at 80 ºC. Color code: **LLI** (red), **8b** (blue) and **9b** (green). (*) Residual solvent peaks and (•) dichloromethane.

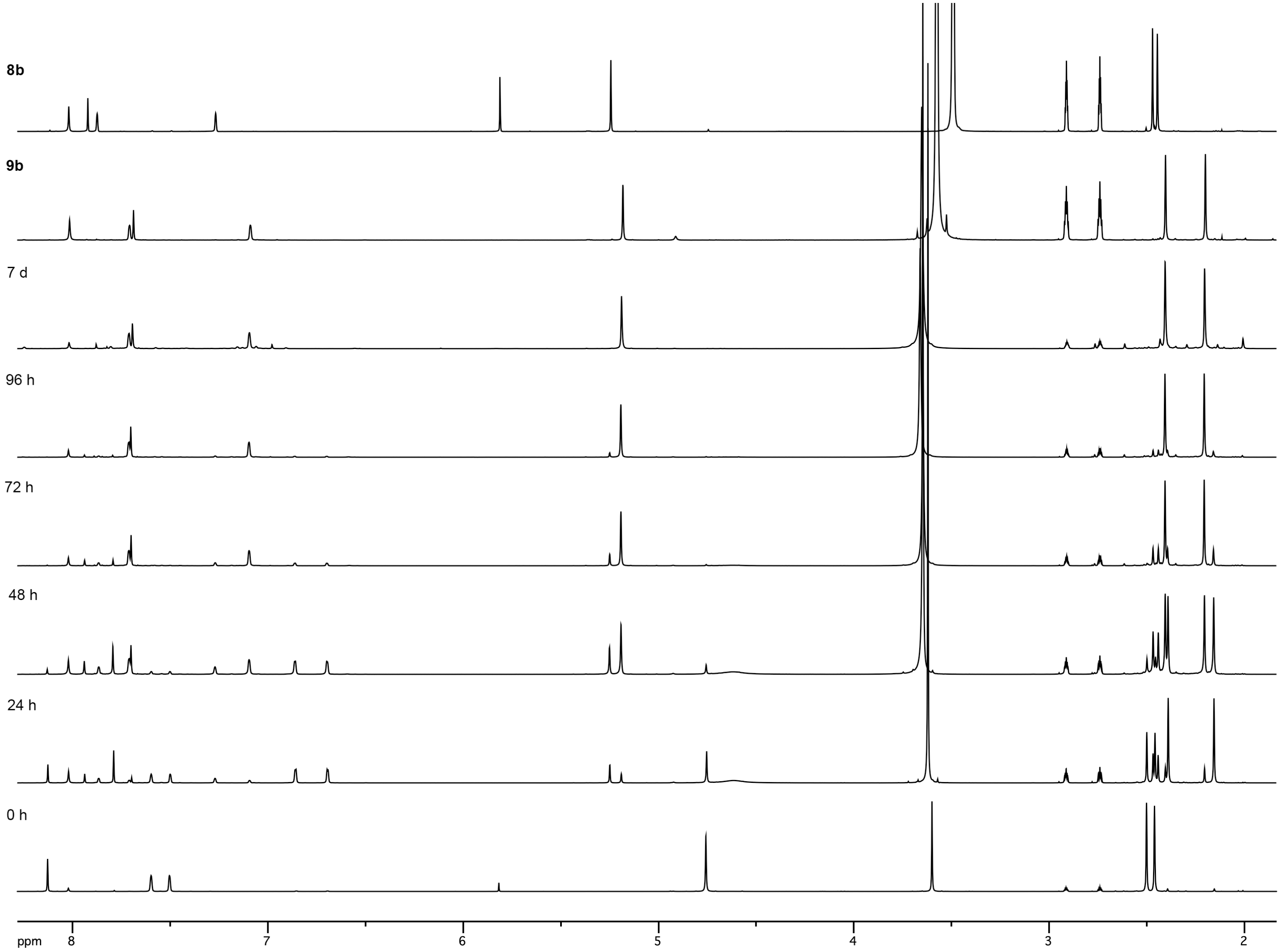

Figure S5b. ^{1}H-NMR (400 MHz) monitoring of the reaction of 5-azidomethyltriazole **1b** with **NaN_3 (1.5 equiv)** in DMF-d_7 at 80 ºC.

3. Computed structures of 1*H*- and 2*H* tautomeric forms of 9b. Theoretical ^{13}C NMR chemical shifts

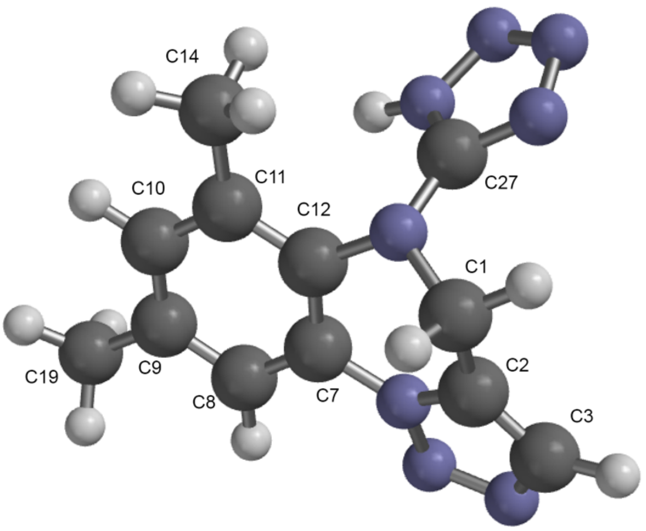

Figure S6. Geometry optimized structure of the N-1 tautomer **9b** at the (PCM, DMF)/wb97XD/6-31+g(d,p) computational level.

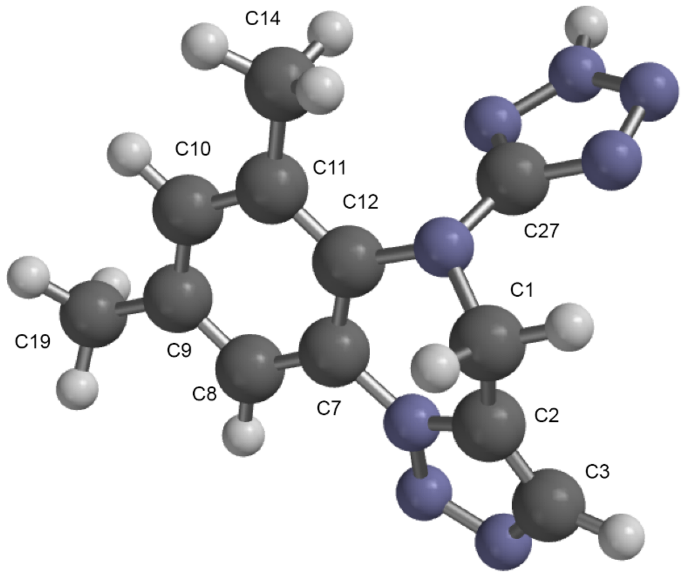

Figure S7. Geometry optimized structure of the N-2 tautomer **9b** at the (PCM, DMF)/wb97XD/6-31+g(d,p) computational level.

Table S3. Computed ^{13}C NMR chemical shifts for the geometry optimized structures of N-1 and N-2 tautomers of **9b** at the (GIAO,DMSO)B3LYP/6-311++G(d,p)//(PCM, DMF)/wb97XD/6-31+g(d,p) theoretical level.

**Carbon atom number**[a]	**N-1 Tautomer (Computed)**[b]	**N-2 Tautomer (Computed)**[b]	**^{13}C NMR Data of 9b**[c]
C1	44.2	43.2	44.3
C2	132.1	132.3	131.2
C3	130.0	129.5	129.4
C7	130.4	129.8	129.5
C8	115.8	114.7	114.5
C9	142.1	140.1	135.1
C10	131.5	130.5	130.6
C11	136.6	138.1	135.1
C12	125.0	127.4	128.5
C14	17.0	18.0	18.1
C19	21.4	21.2	20.7
C27	155.3	164.4	163.6

[a] See figures S6 and S7. [b] The equation reported in ref 1 was used to transform absolute shieldings into chemical shifts. [c] Measured at 100 MHz, DMSO-d_6, 298 K.

4. ^{1}H and ^{13}C{^{1}H} NMR spectra of all new-synthesized compounds (HMBC, HSQC, COSY and NOESY of selected compounds are also included)

^{1}H NMR (400 MHz, $CDCl_3$, 298 K):

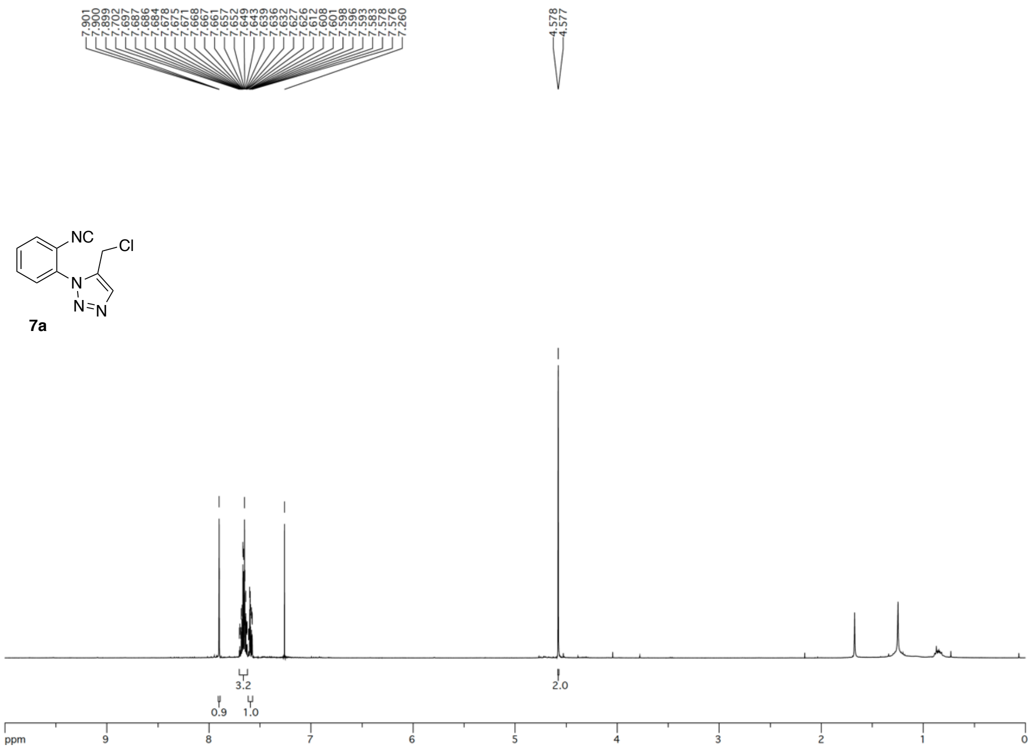

^{13}C{^{1}H} NMR (100 MHz, $CDCl_3$, 298 K):

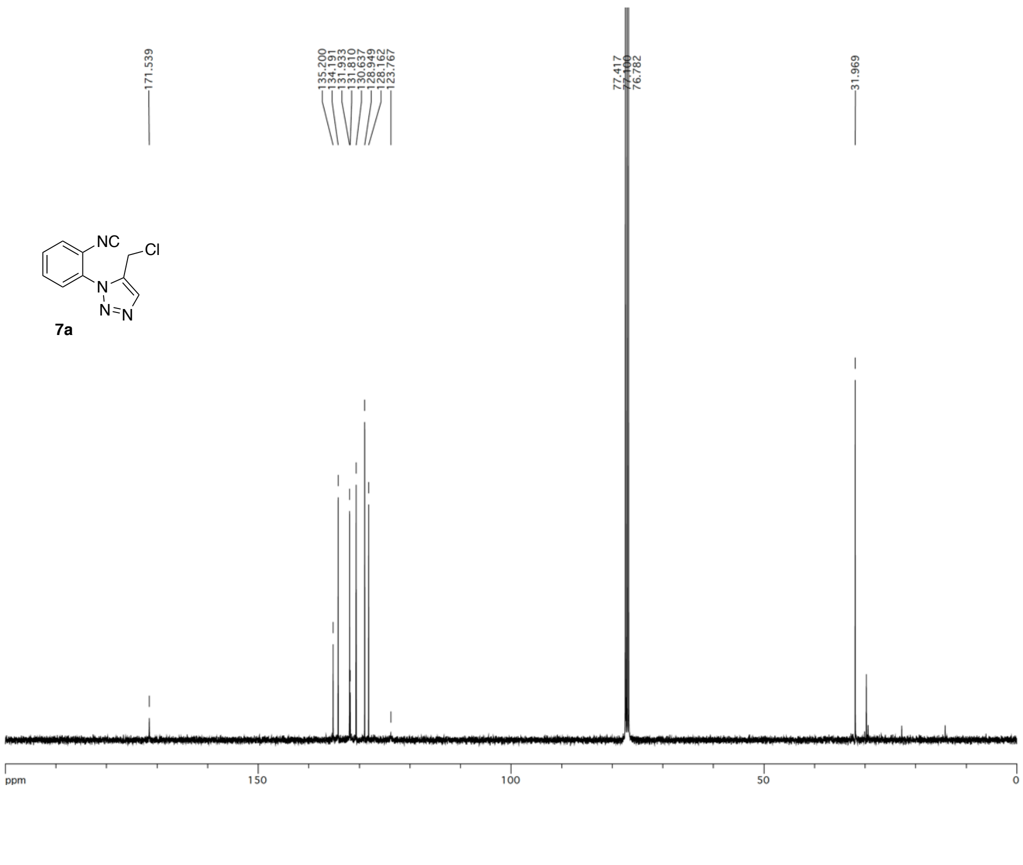

Dept-135 (100 MHz, $CDCl_3$, 298 K):

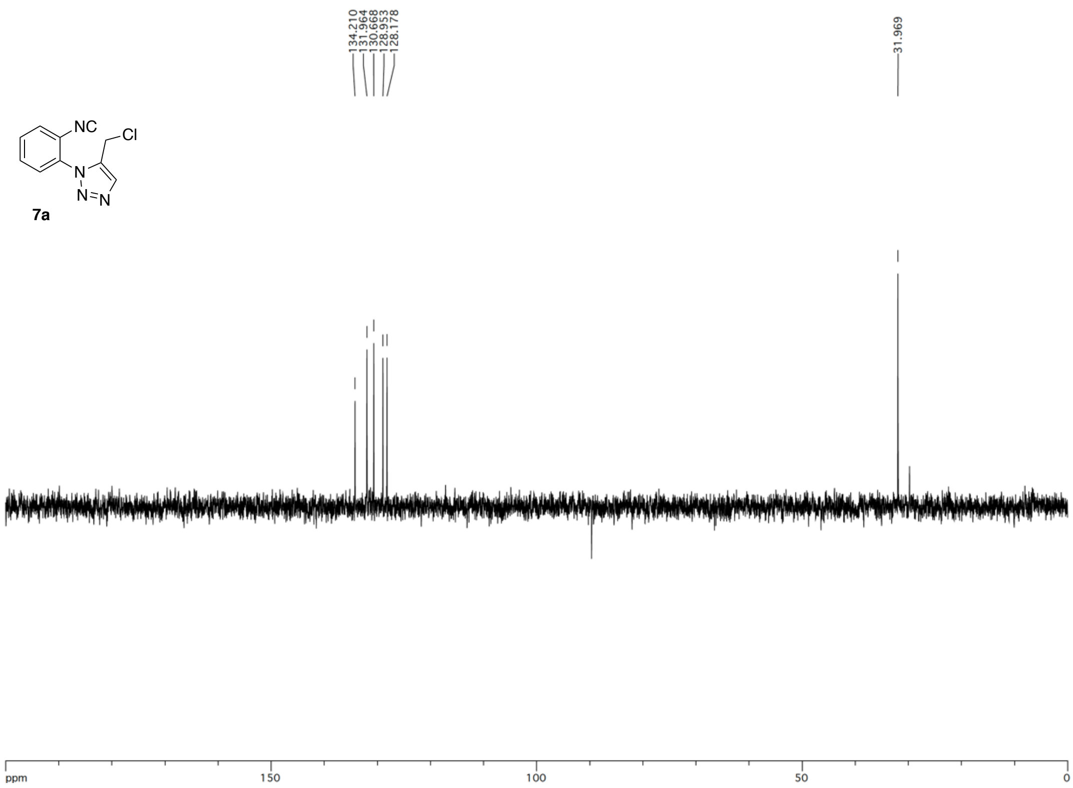

^{1}H NMR (400 MHz, $CDCl_3$, 298 K):

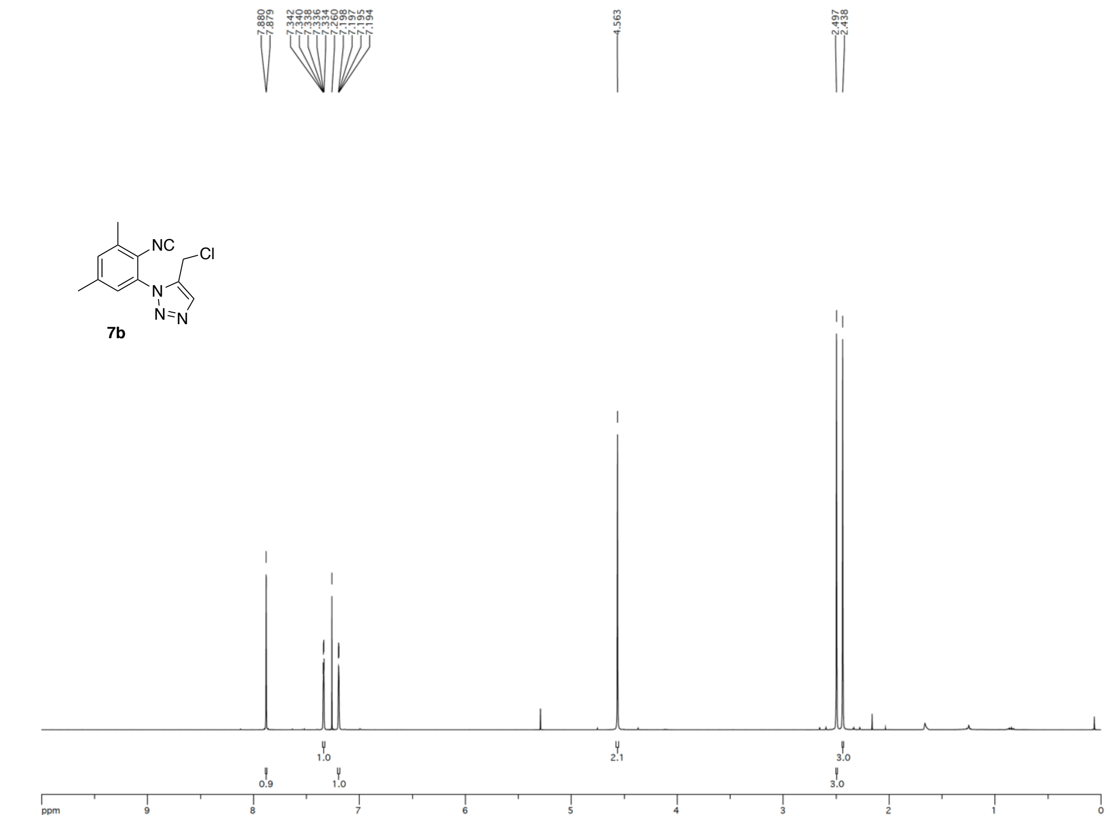

^{13}C{^{1}H} NMR (100 MHz, $CDCl_3$, 298 K):

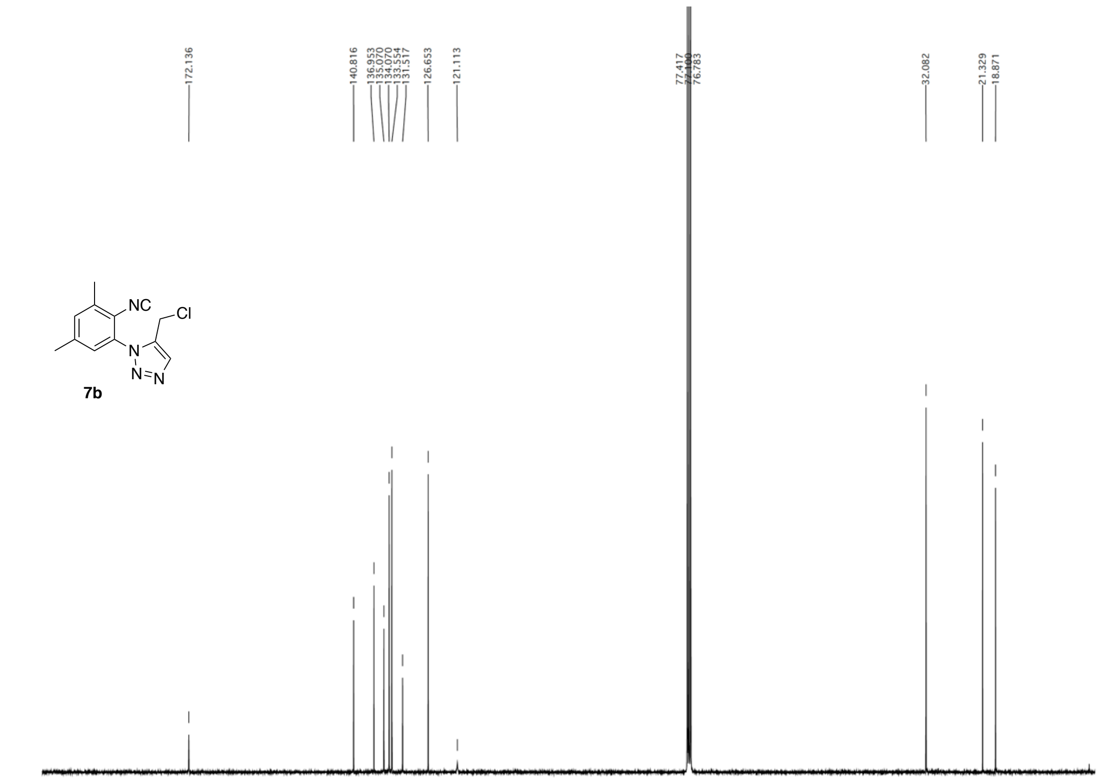

Dept-135 (100 MHz, $CDCl_3$, 298 K):

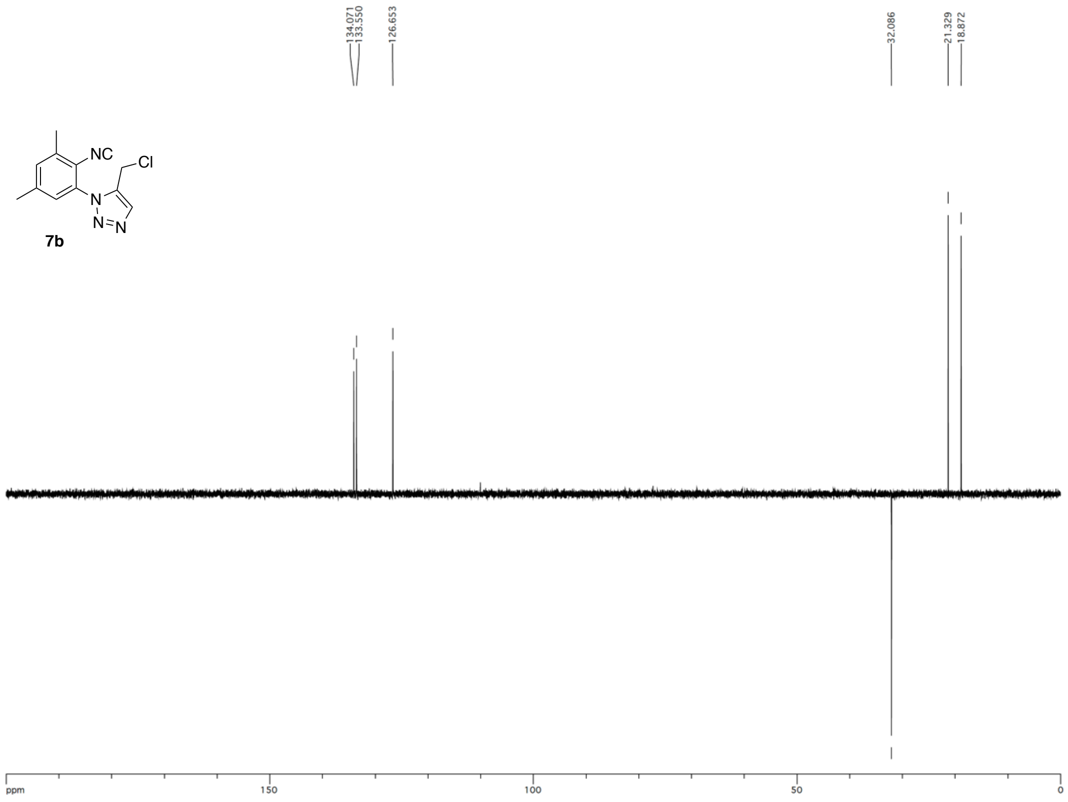

^{1}H NMR (400 MHz, $CDCl_3$, 298 K):

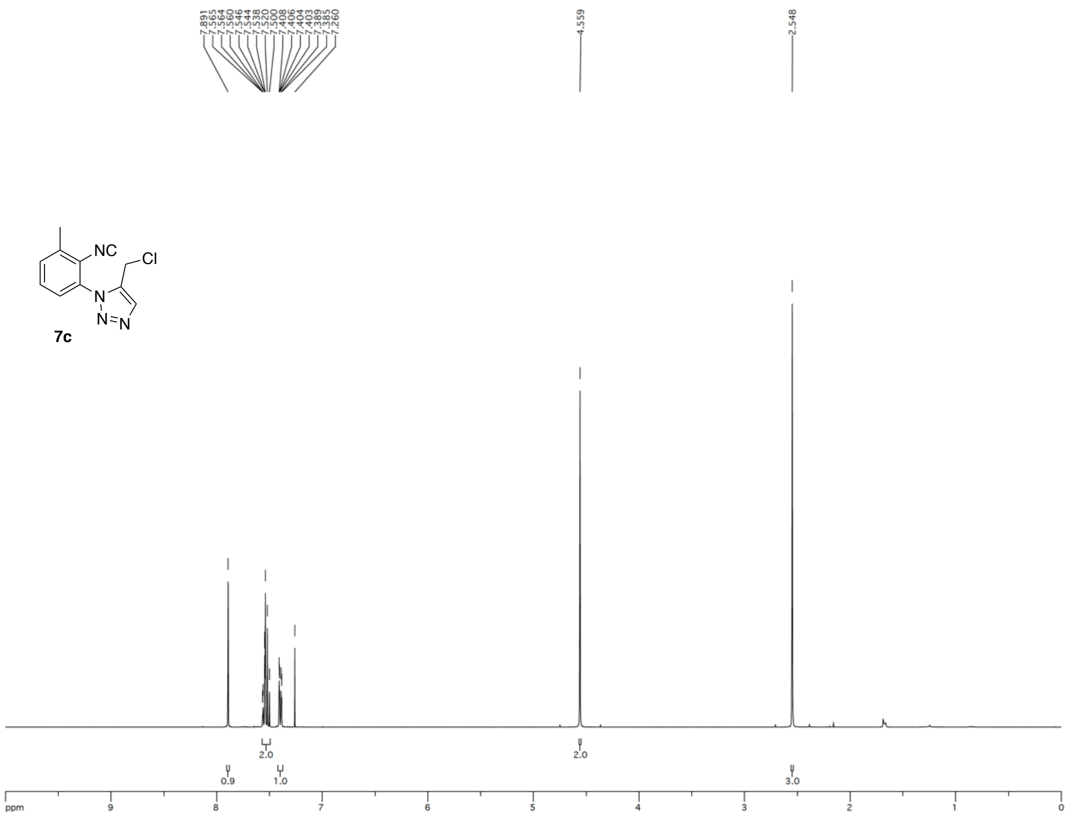

^{13}C{^{1}H} NMR (100 MHz, $CDCl_3$, 298 K):

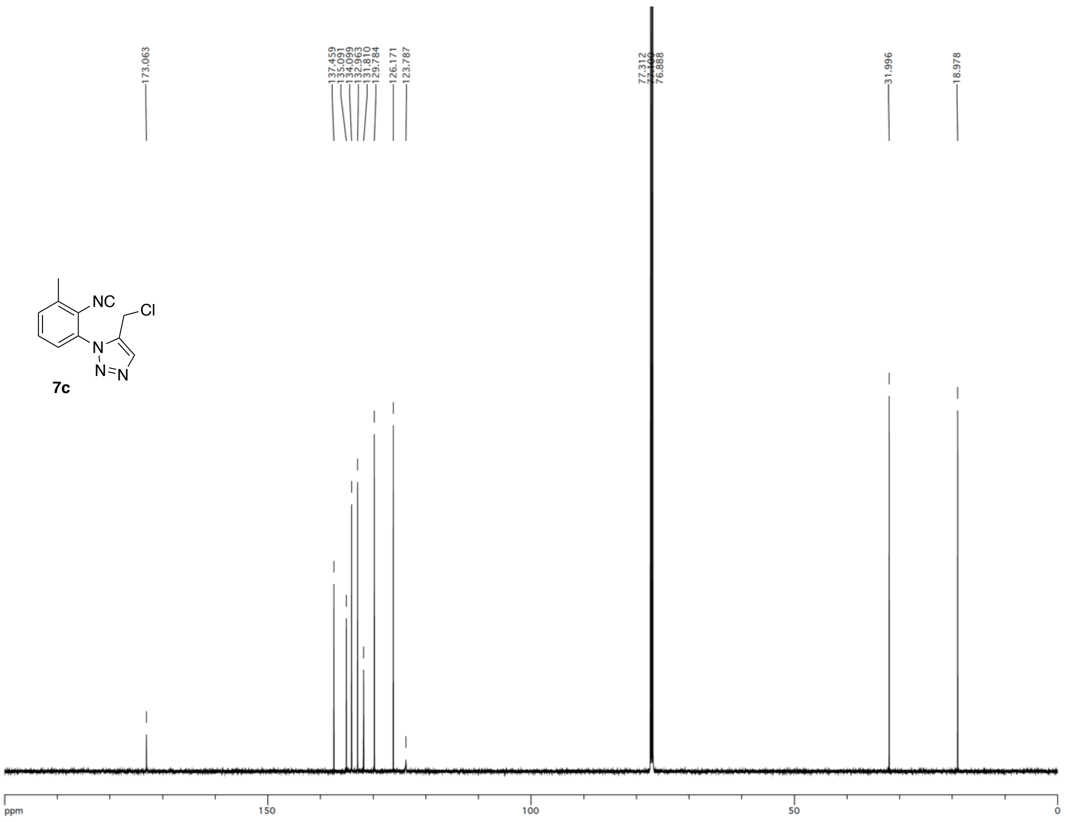

Dept-135 (100 MHz, CDCl3, 298 K):

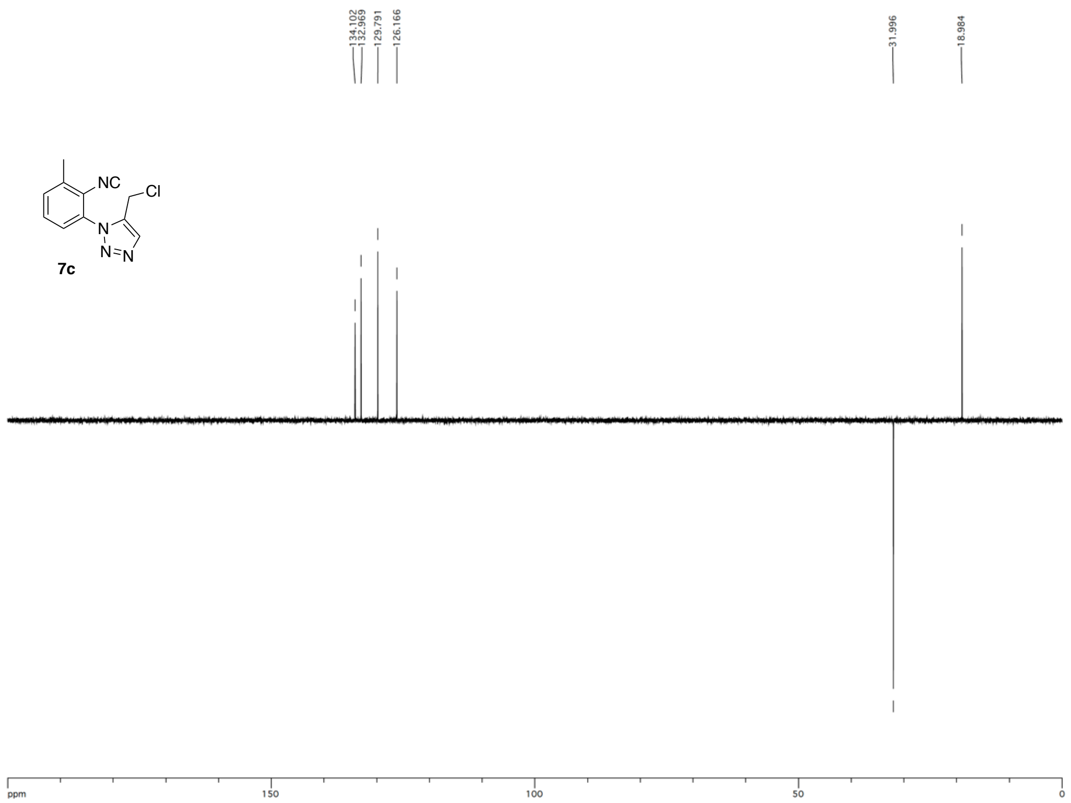

^{1}H NMR (400 MHz, $CDCl_3$, 298 K):

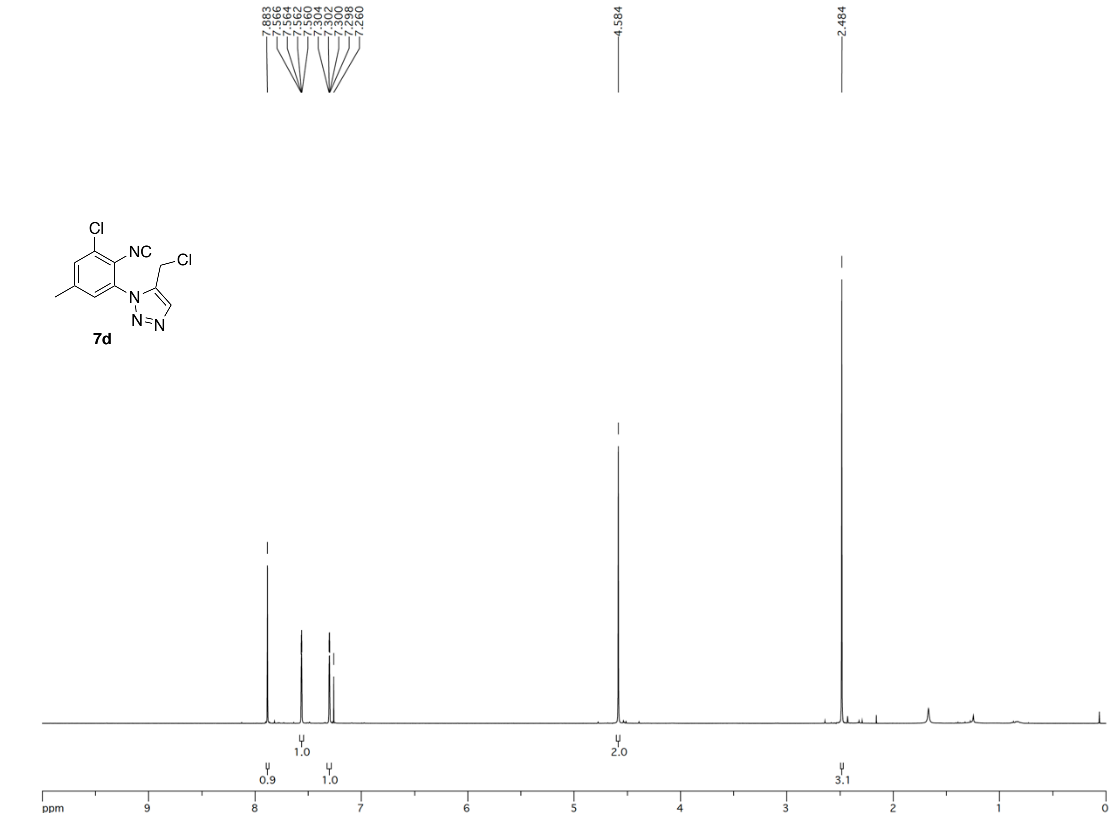

$^{13}C\{^{1}H\}$ NMR (100 MHz, $CDCl_3$, 298 K):

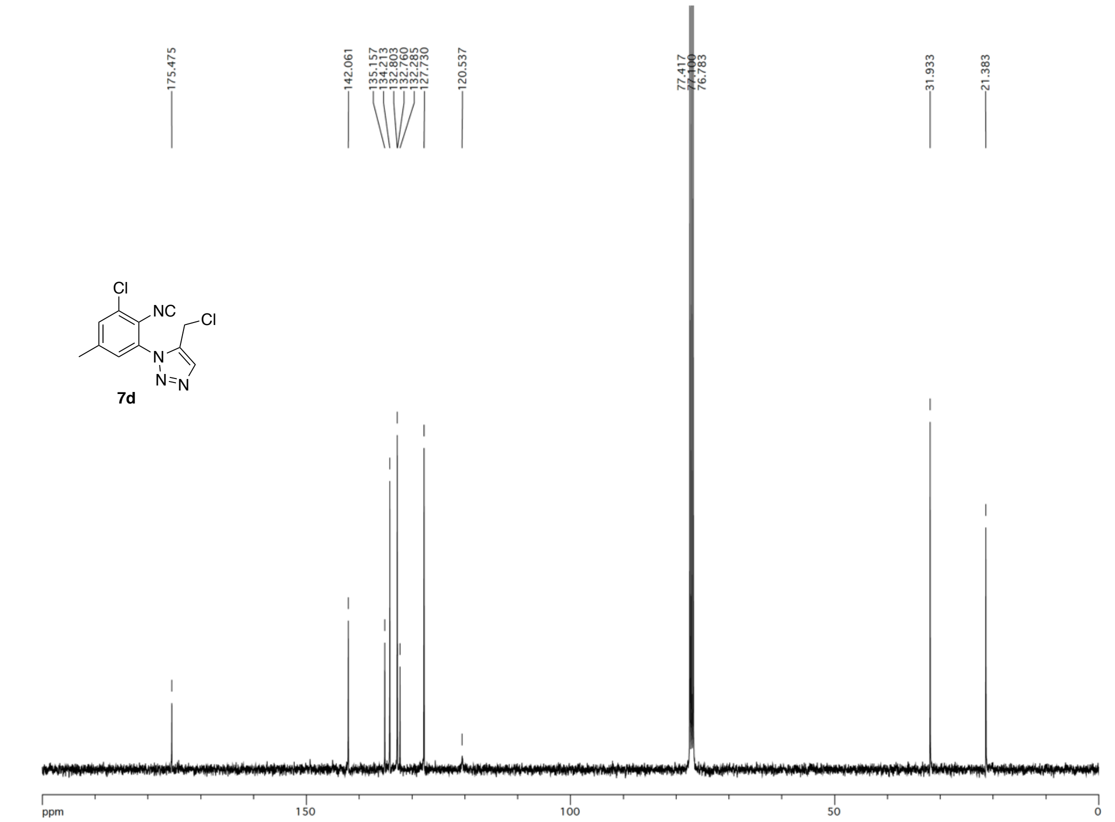

Dept-135 (100 MHz, CDCl$_3$, 298 K):

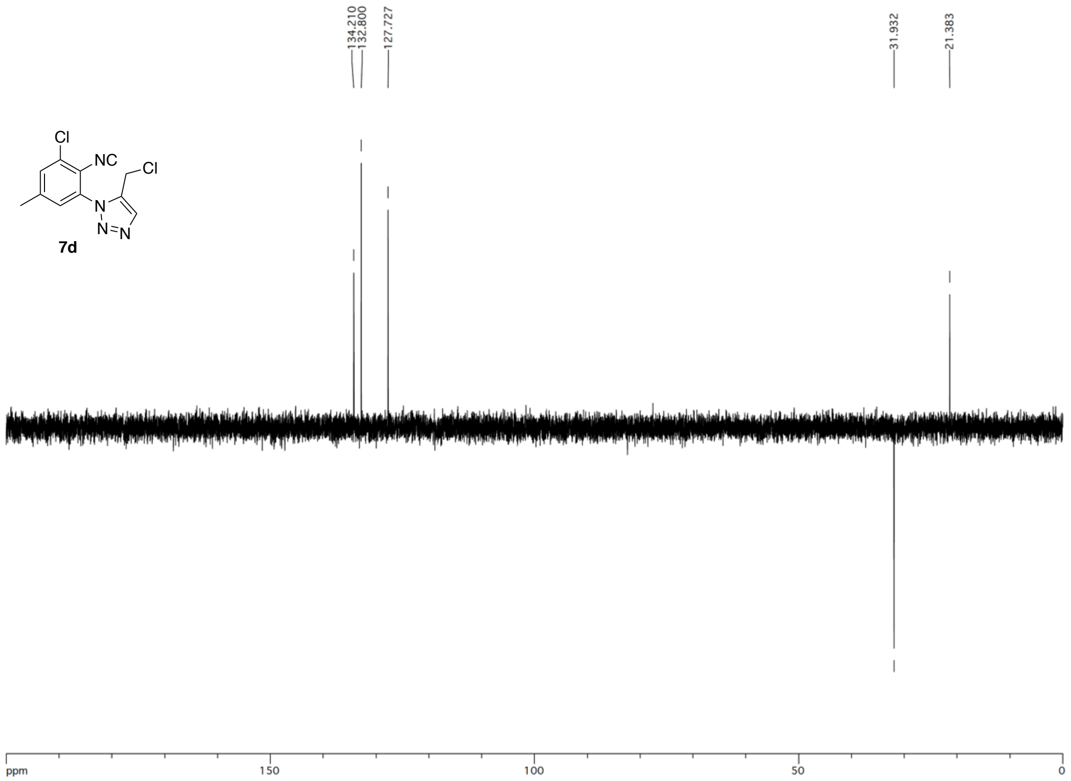

^{1}H NMR (400 MHz, $CDCl_3$, 298 K):

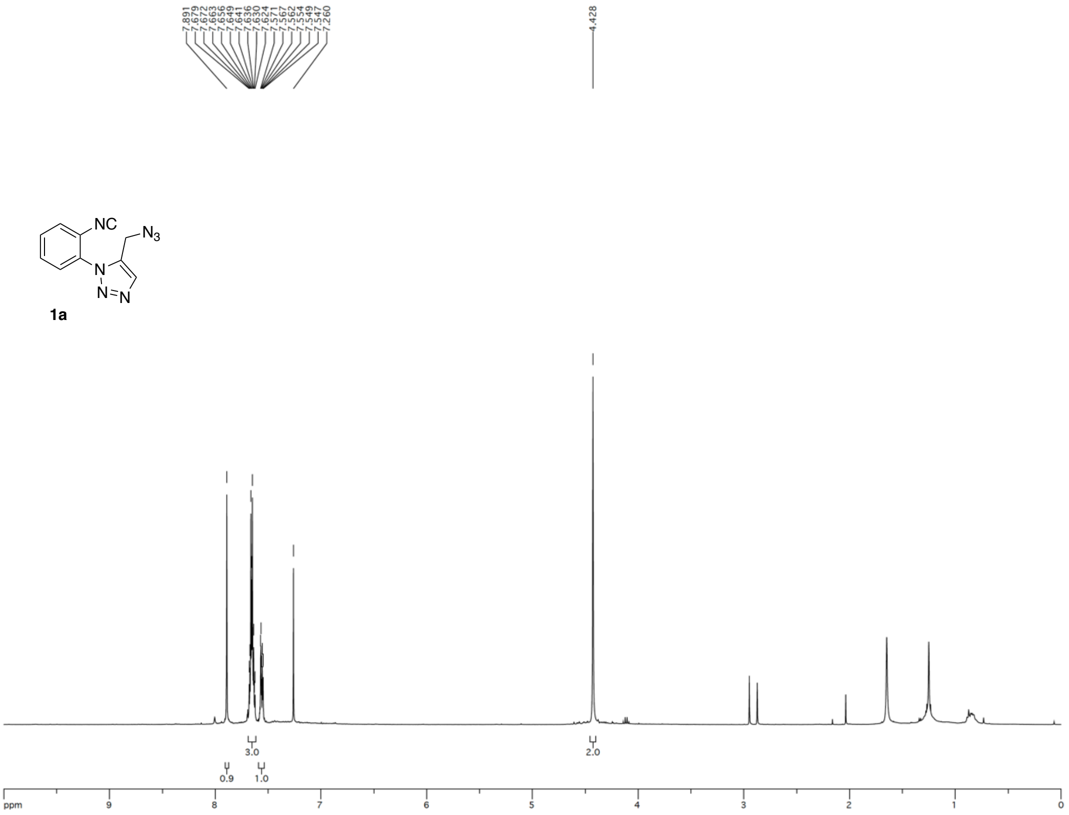

^{13}C{^{1}H} NMR (100 MHz, $CDCl_3$, 298 K):

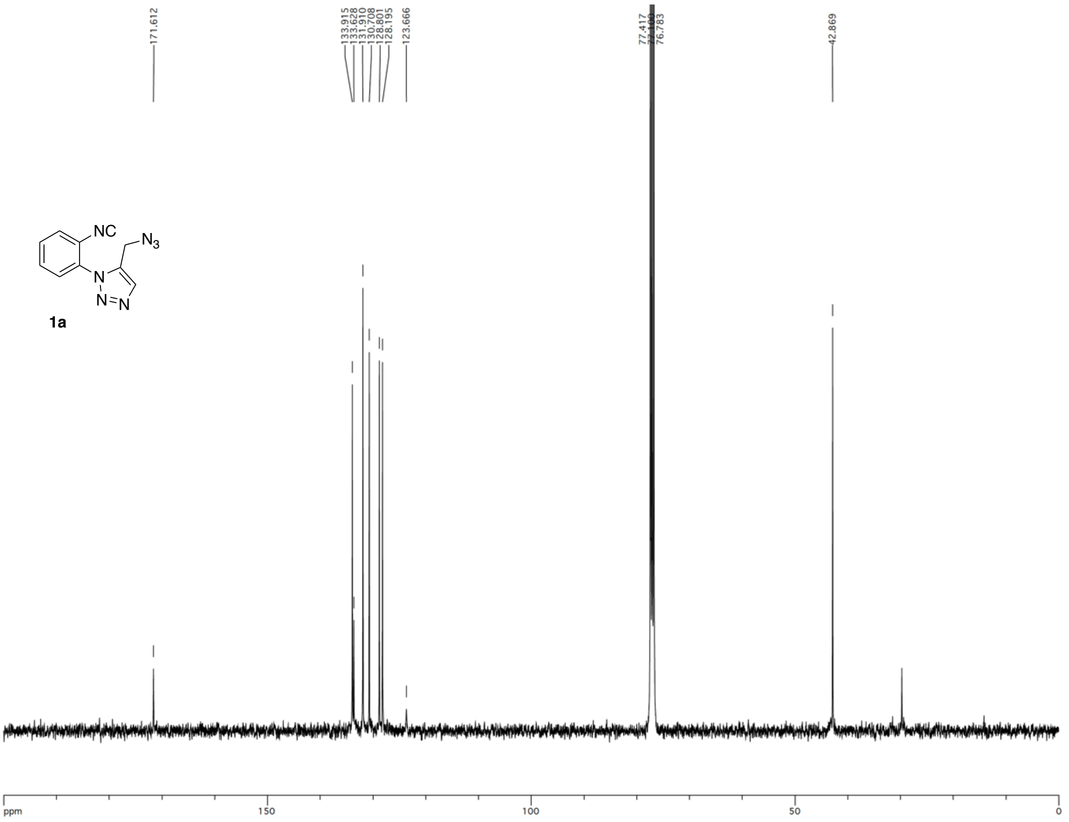

Dept-135 (100 MHz, CDCl$_3$, 298 K):

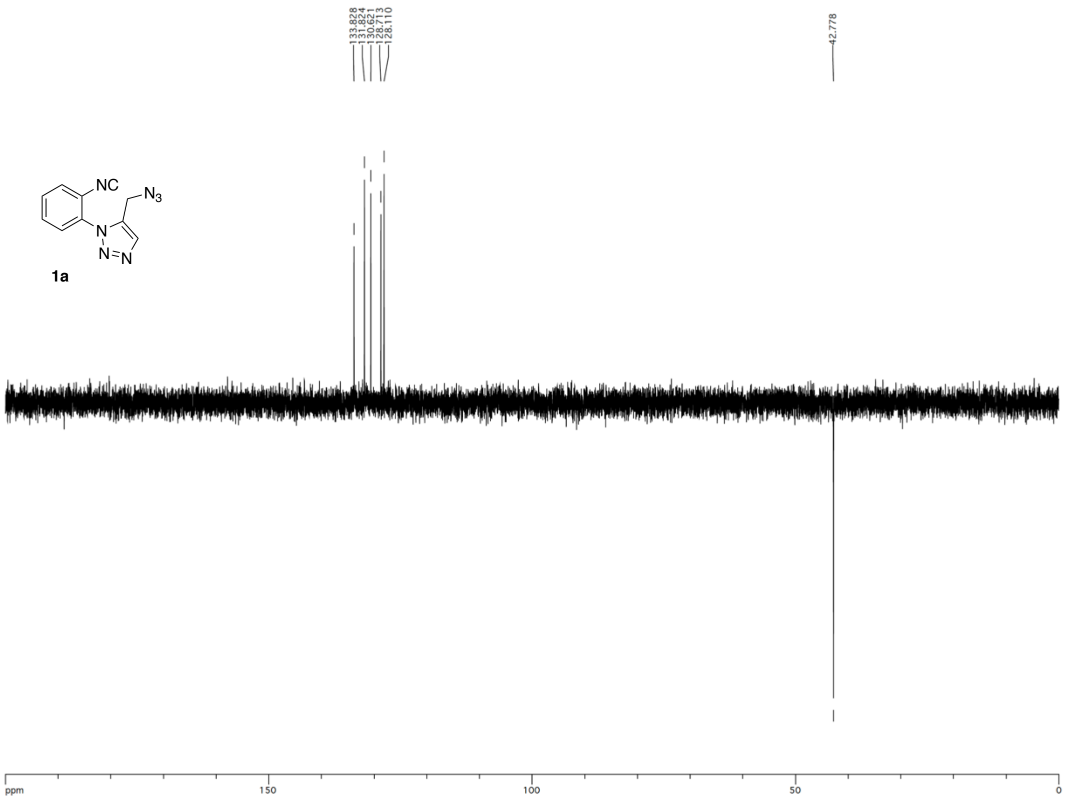

^{1}H NMR (400 MHz, $CDCl_3$, 298 K):

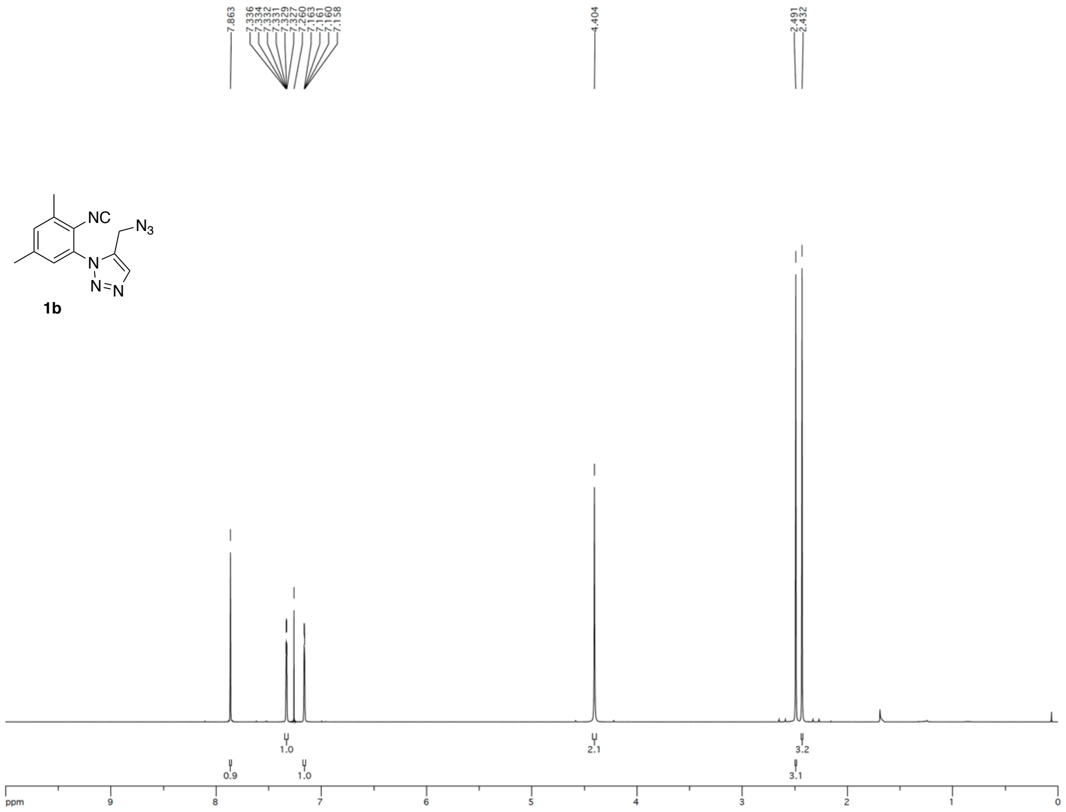

$^{13}C\{^{1}H\}$ NMR (150 MHz, $CDCl_3$, 298 K):

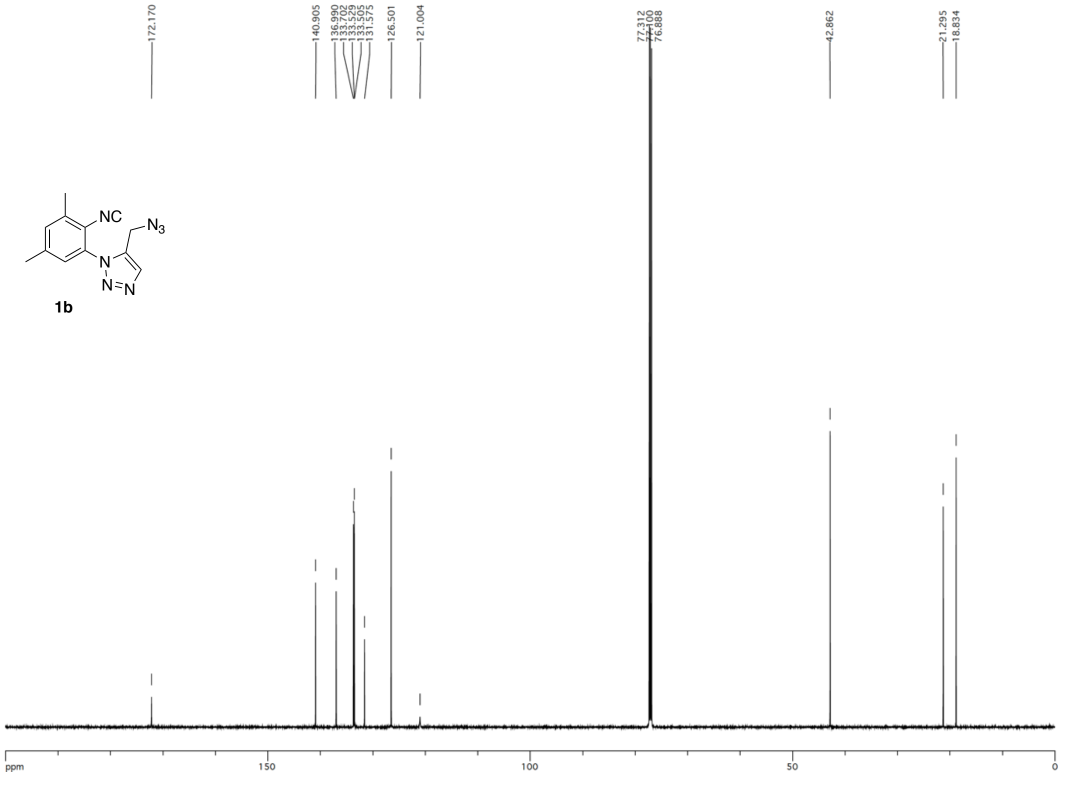

Dept-135 (100 MHz, $CDCl_3$, 298 K):

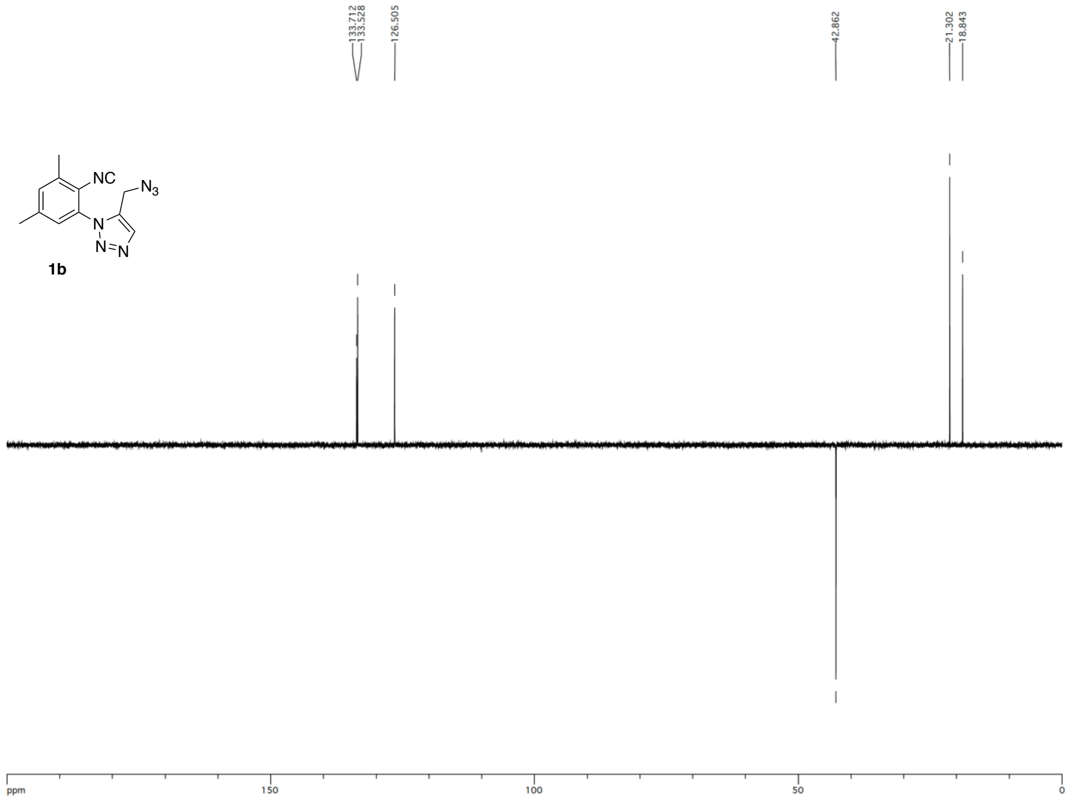

^{1}H NMR (400 MHz, $CDCl_3$, 298 K):

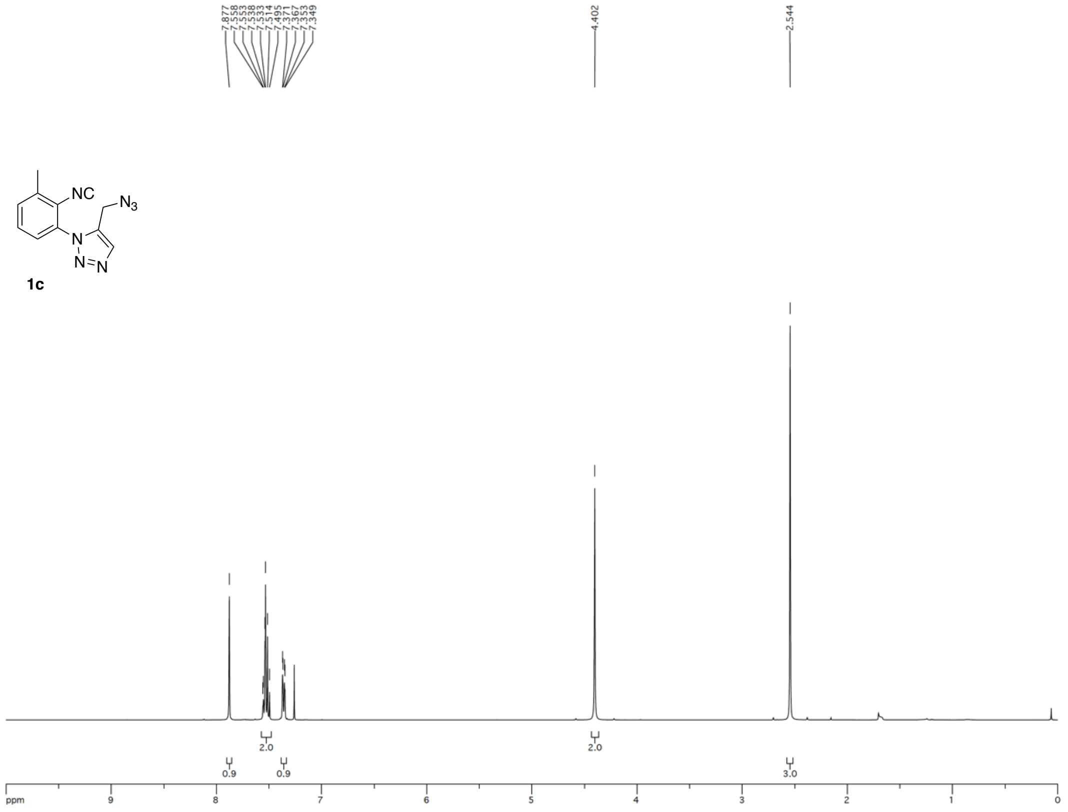

^{13}C{^{1}H} NMR (150 MHz, $CDCl_3$, 298 K):

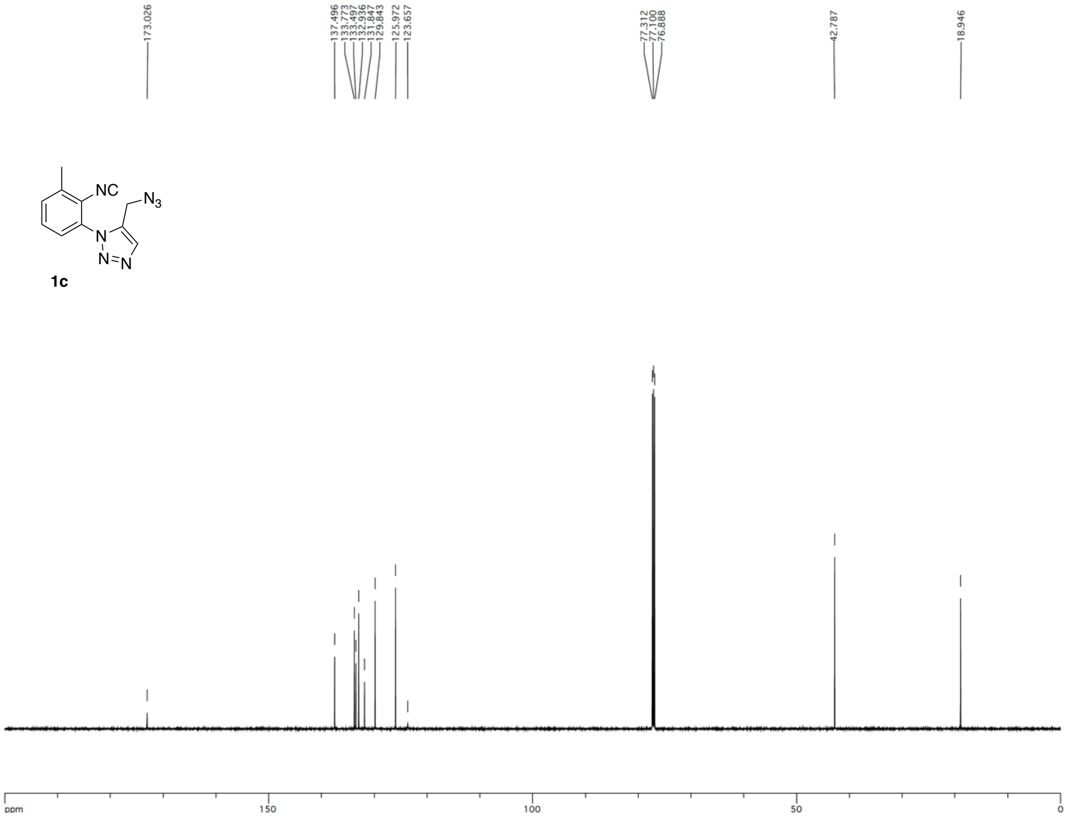

Dept-135 (100 MHz, $CDCl_3$, 298 K):

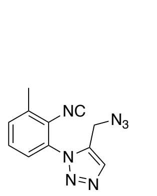

1c

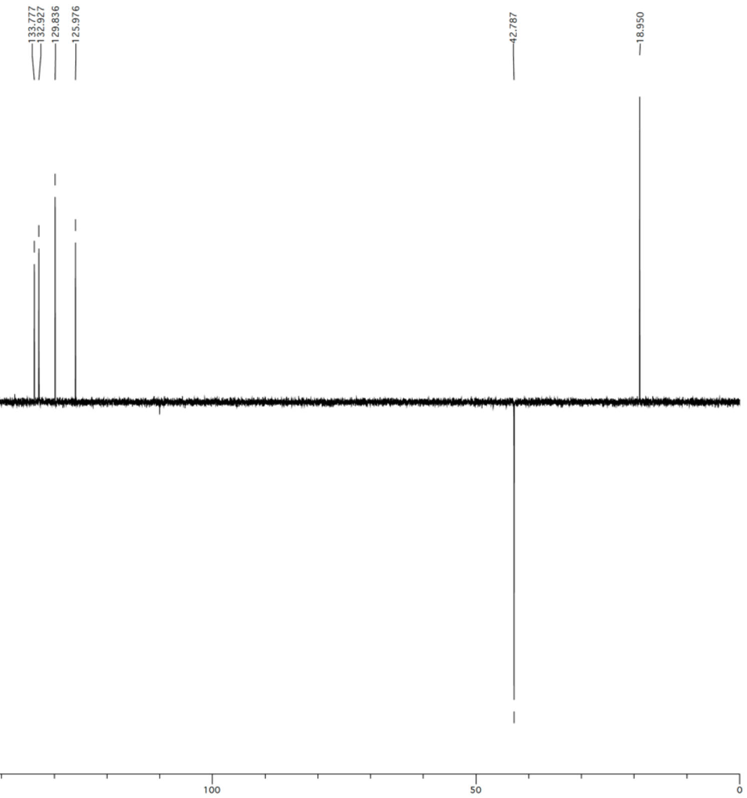

^{1}H NMR (400 MHz, $CDCl_3$, 298 K):

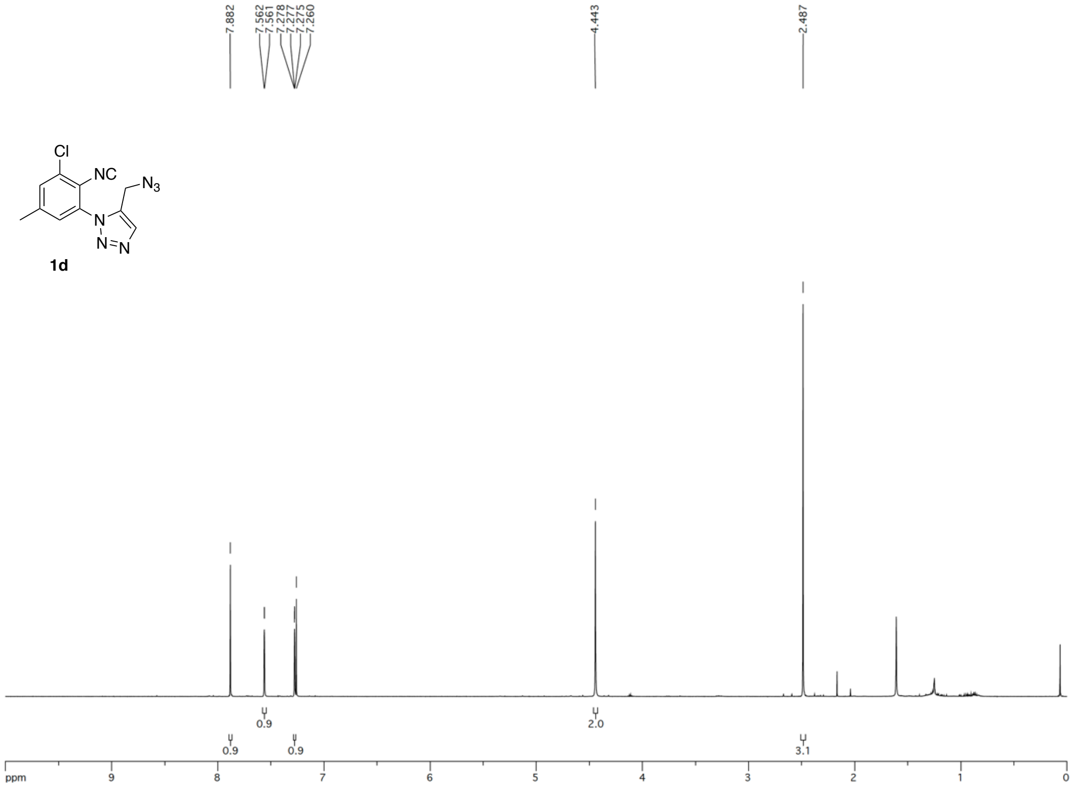

^{13}C{^{1}H} NMR (100 MHz, $CDCl_3$, 298 K):

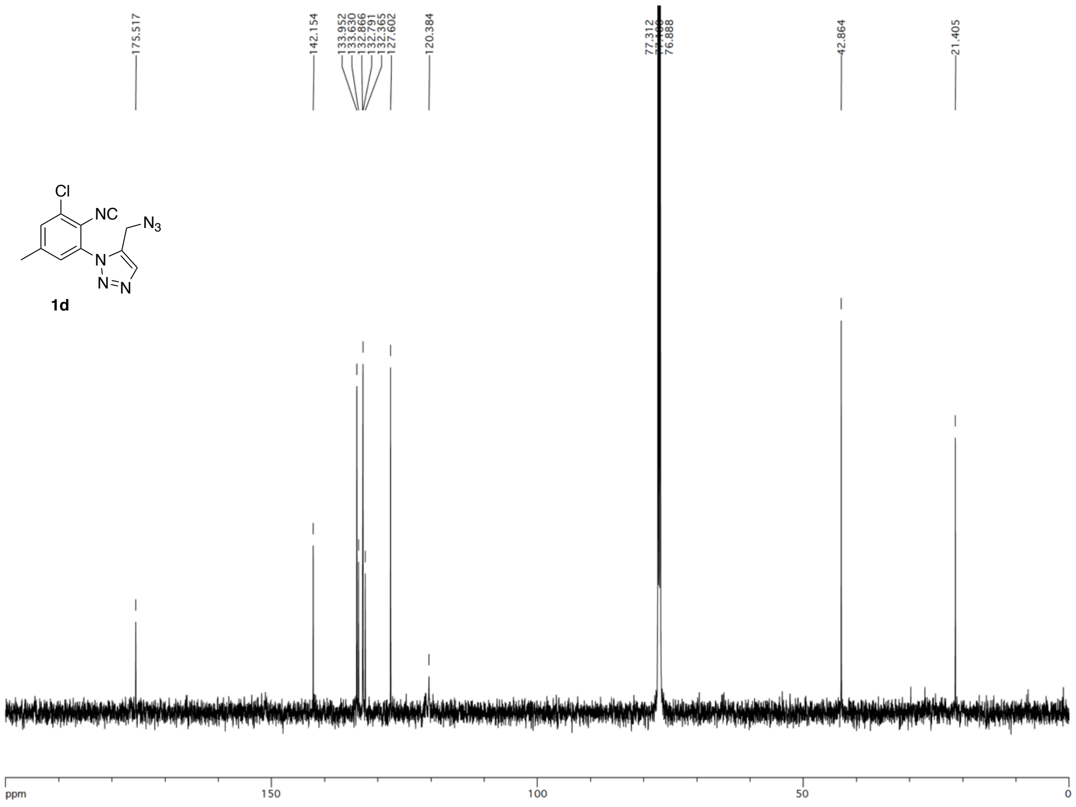

Dept-135 (100 MHz, $CDCl_3$, 298 K):

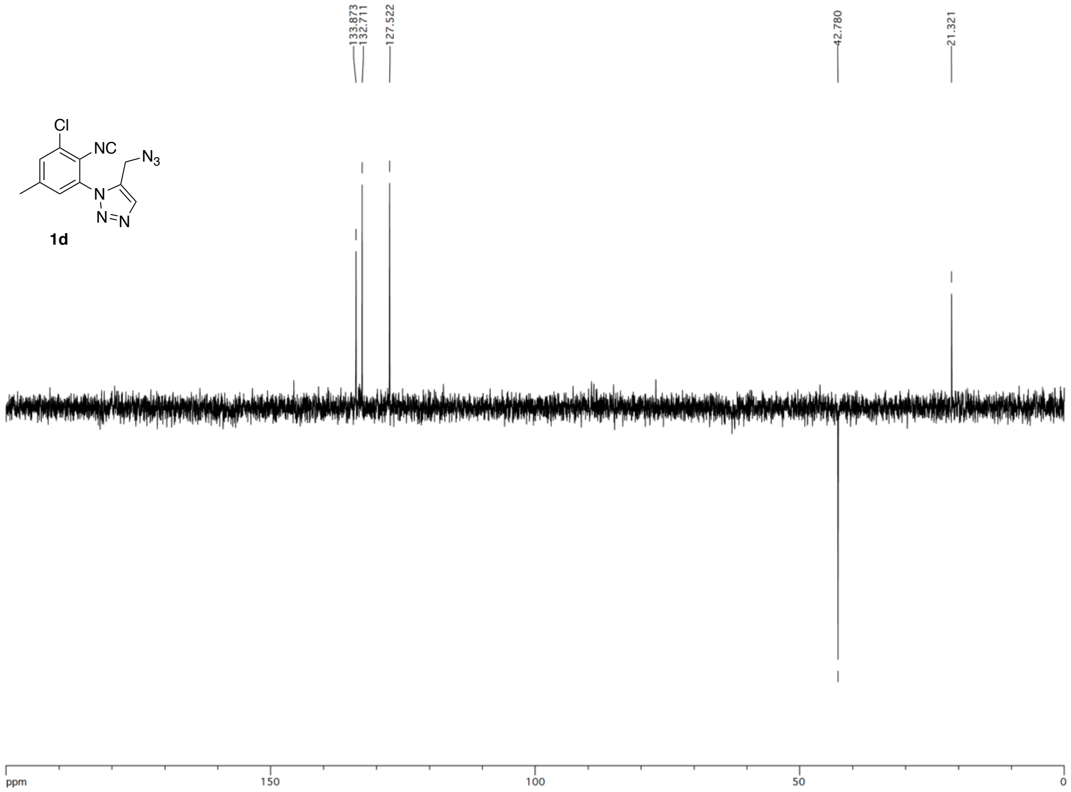

^{1}H NMR (400 MHz, $CDCl_3$, 298 K):

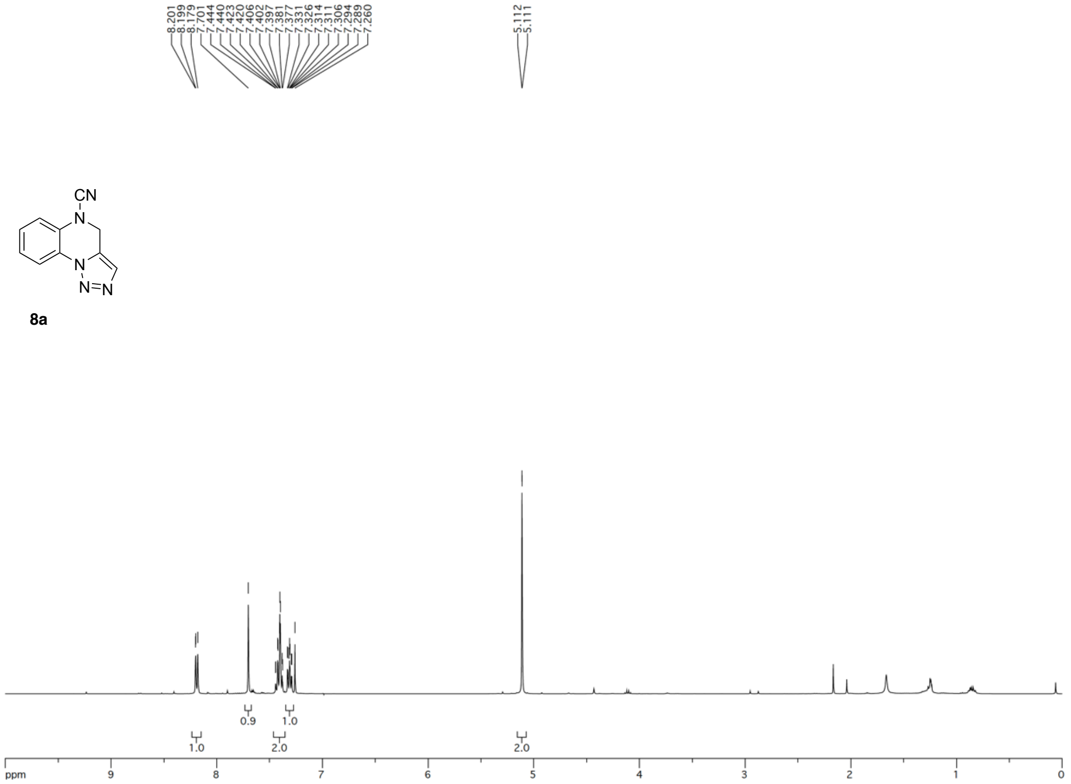

^{13}C{^{1}H} NMR (100 MHz, $CDCl_3$, 298 K):

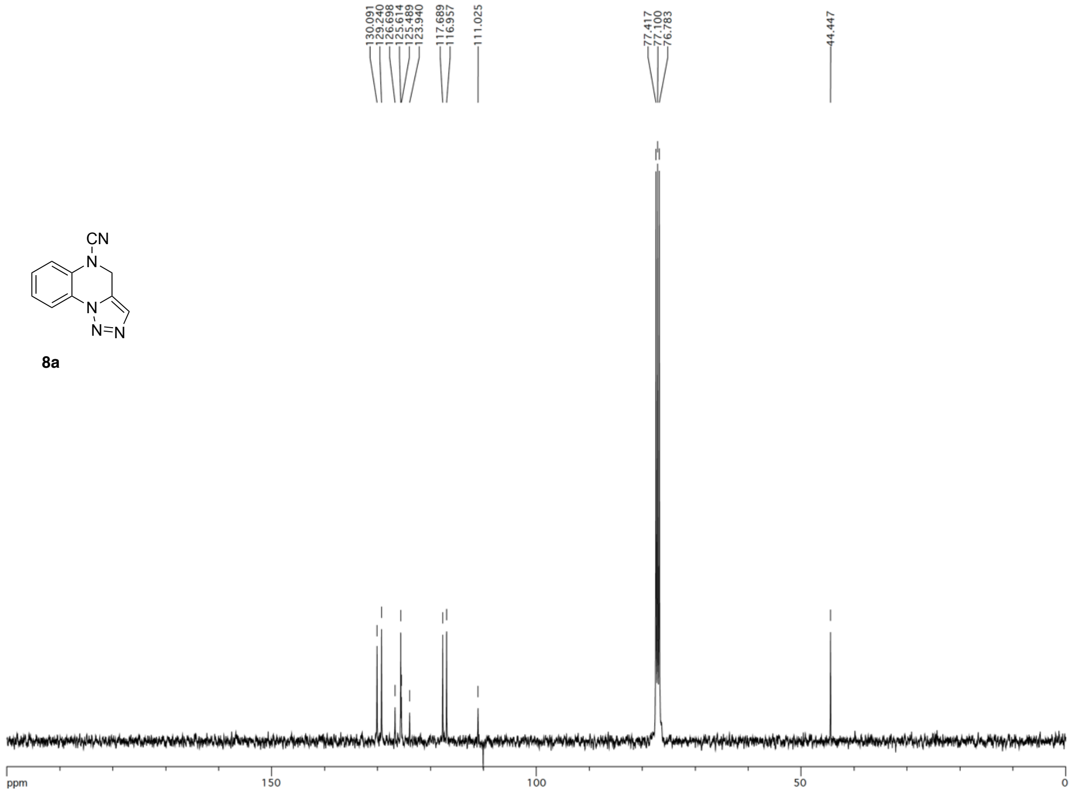

Dept-135 (100 MHz, CDCl₃, 298 K):

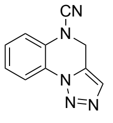

8a

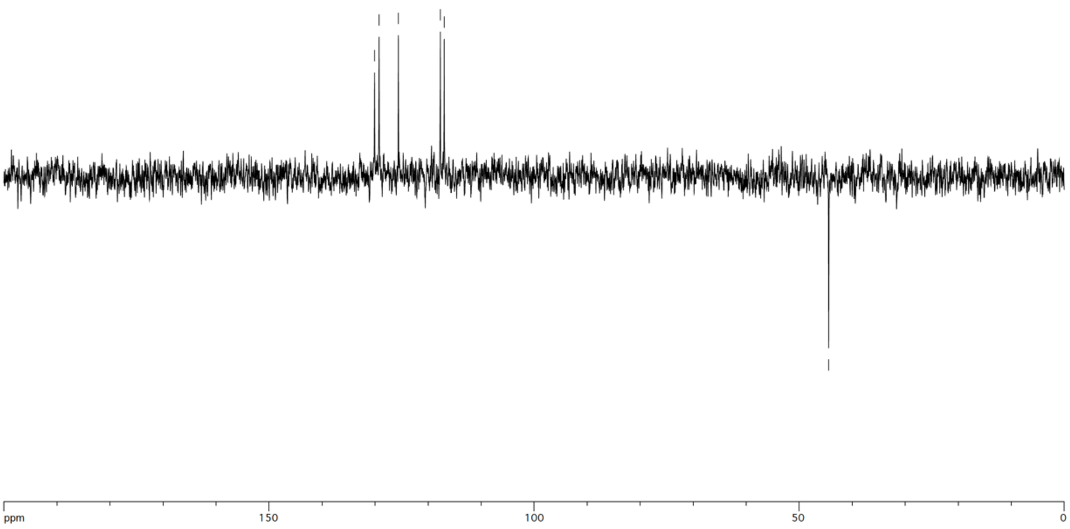

^{1}H NMR (400 MHz, $CDCl_3$, 298 K):

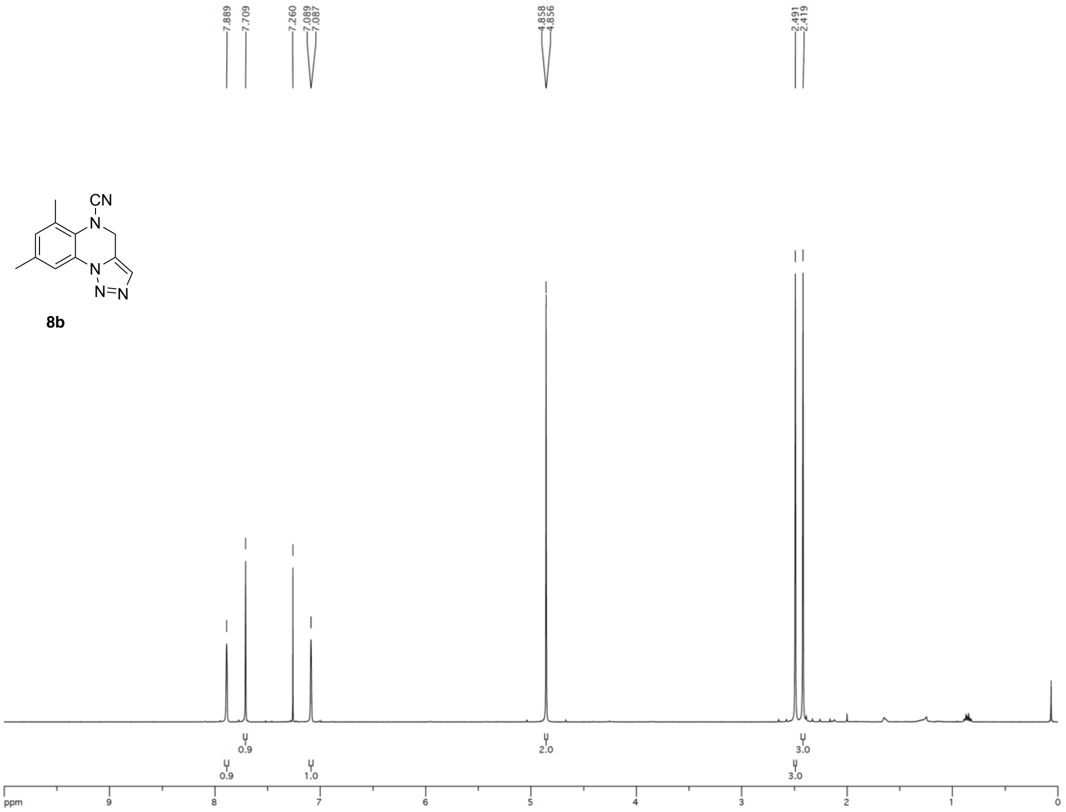

^{13}C{^{1}H} NMR (100 MHz, $CDCl_3$, 298 K):

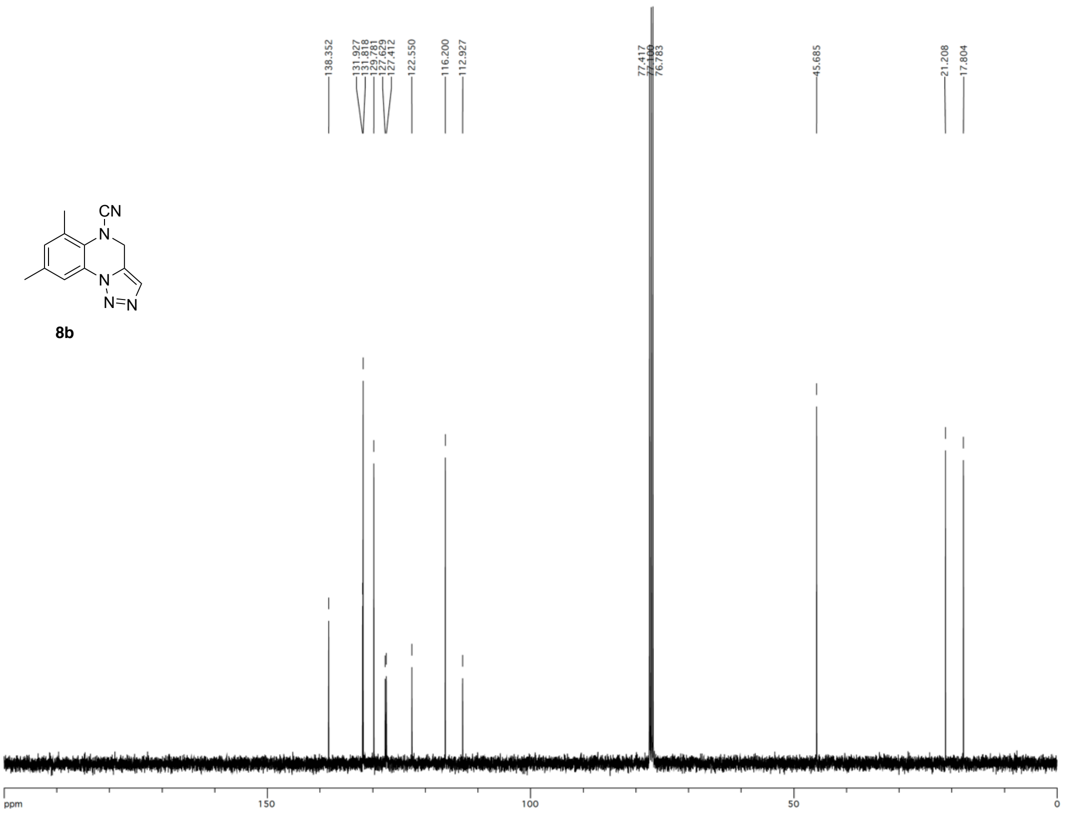

Dept-135 (100 MHz, $CDCl_3$, 298 K):

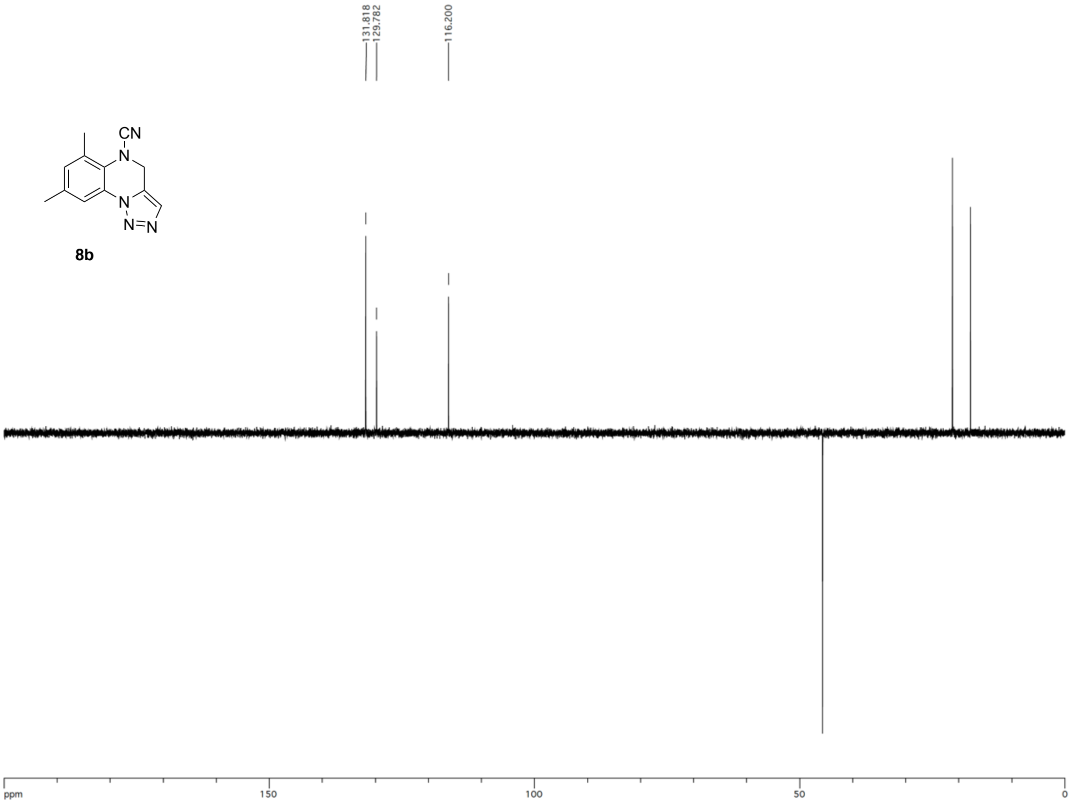

^{1}H NMR (400 MHz, $CDCl_3$, 298 K):

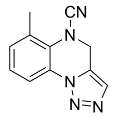

8c

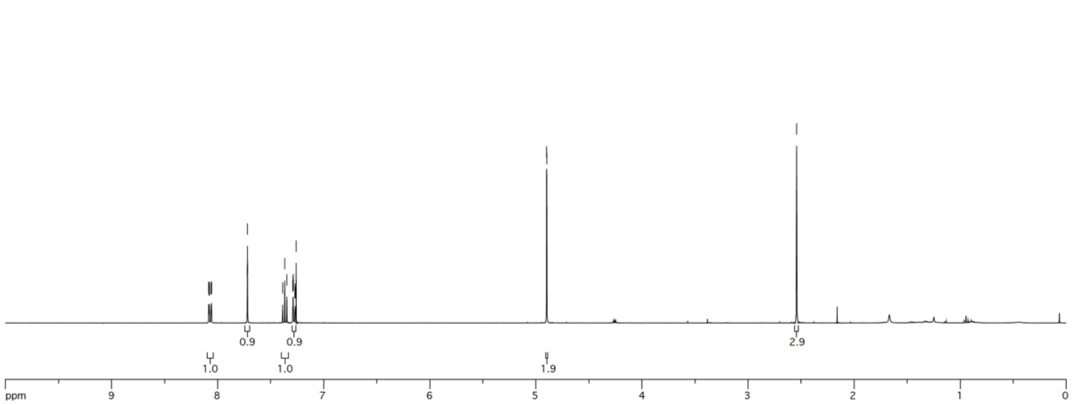

$^{13}C\{^{1}H\}$ NMR (100 MHz, $CDCl_3$, 298 K):

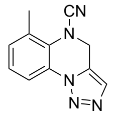

8c

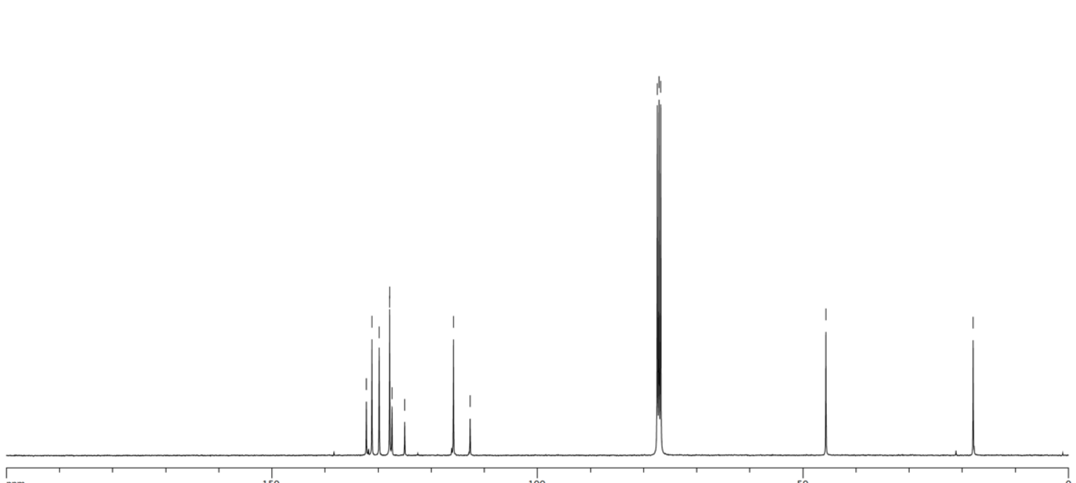

Dept-135 (100 MHz, CDCl₃, 298 K):

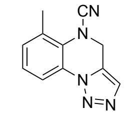

8c

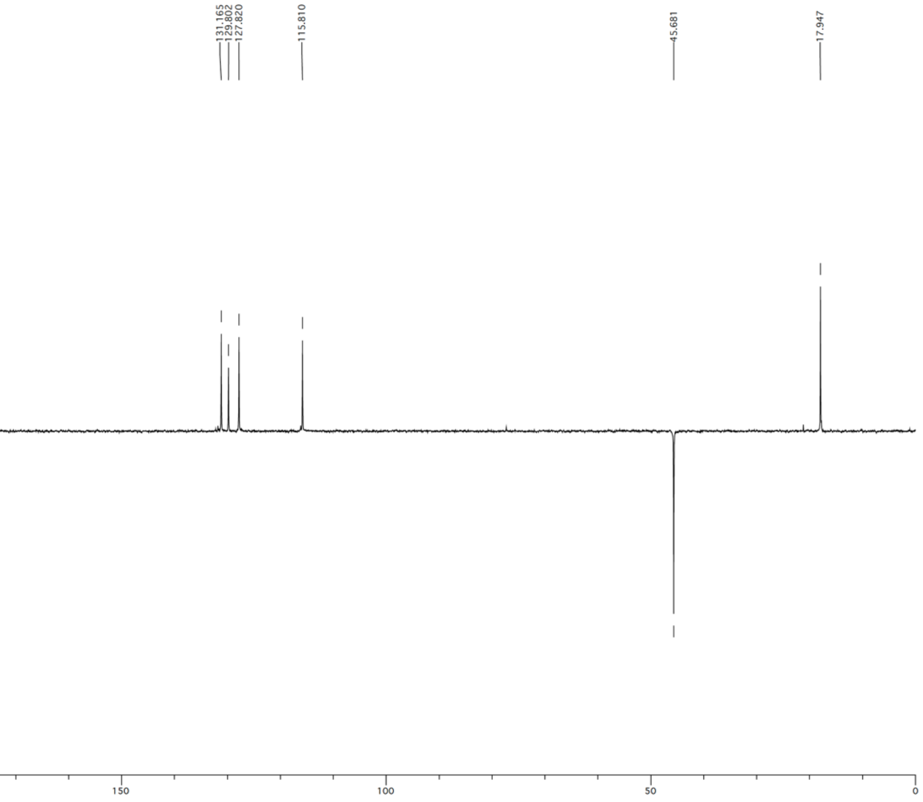

^{1}H NMR (400 MHz, $CDCl_3$, 298 K) Asterisks indicate peaks of starting material (**1d**):

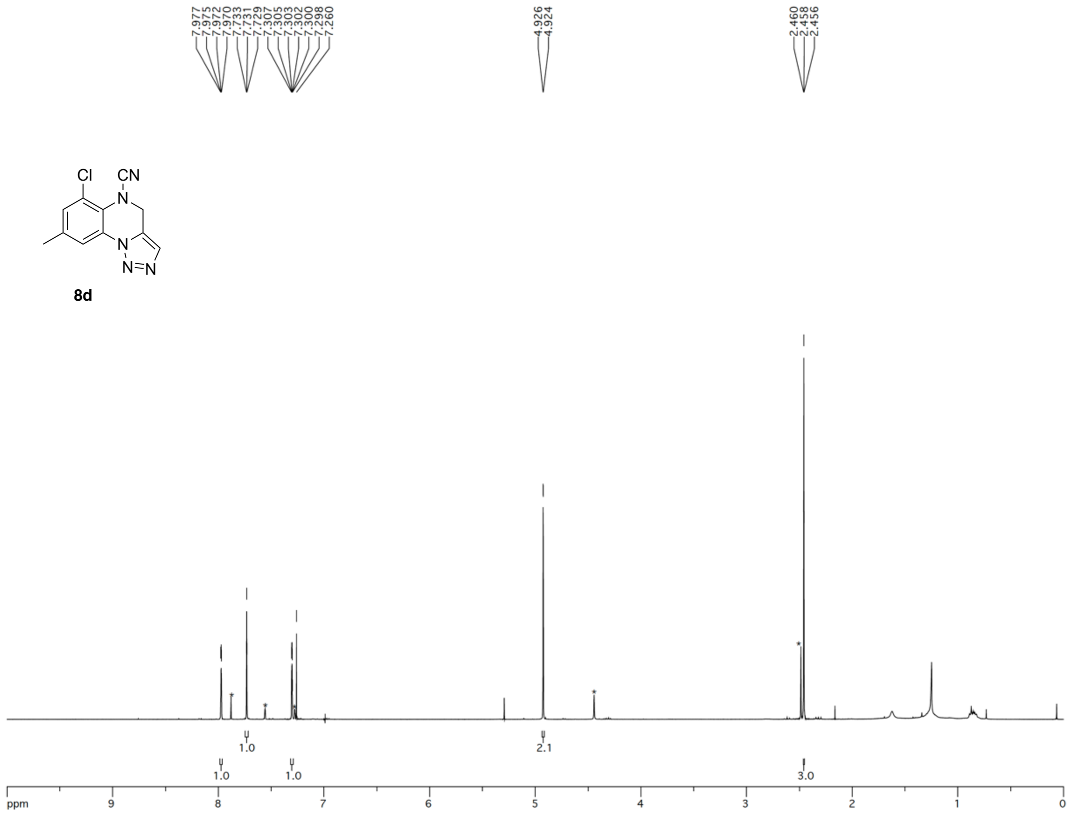

$^{13}C\{^{1}H\}$ NMR (100 MHz, $CDCl_3$, 298 K) Asterisks indicate peaks of starting material (**1d**) and the hash mark indicates the grease peak.

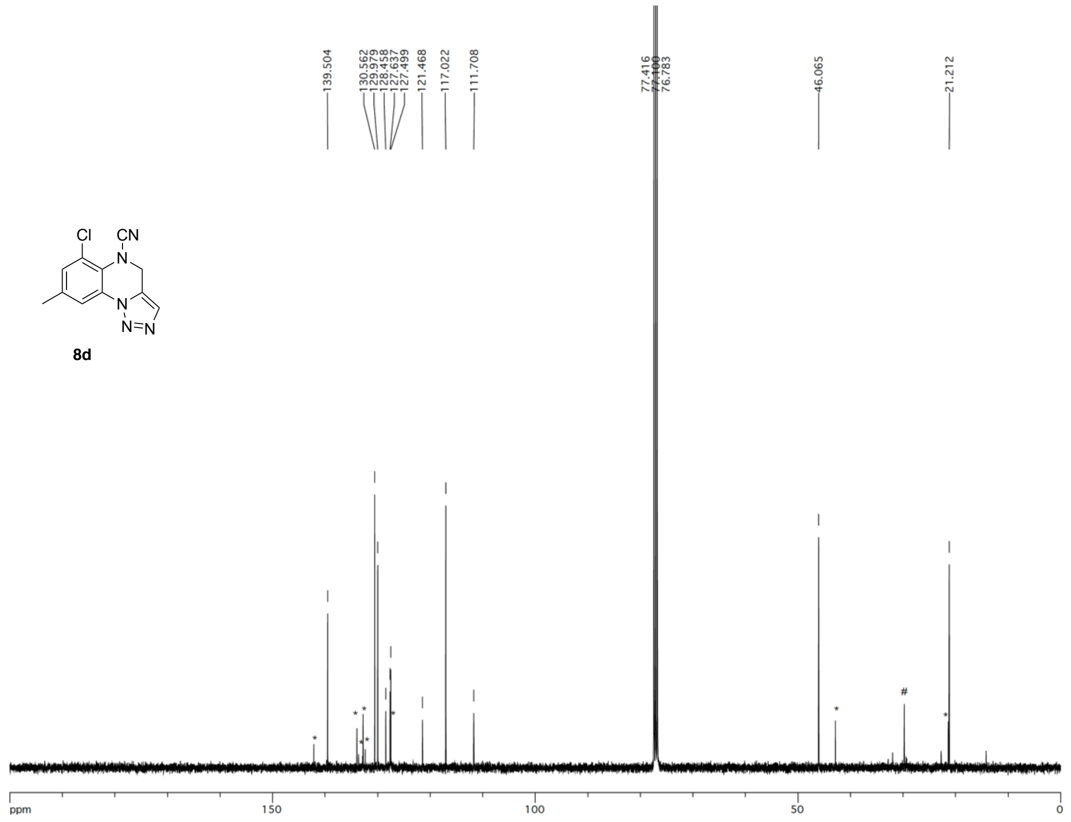

Dept-135 (100 MHz, $CDCl_3$, 298 K) Asterisks indicate peaks of starting material (**1d**) and the hash mark indicates the grease peak:

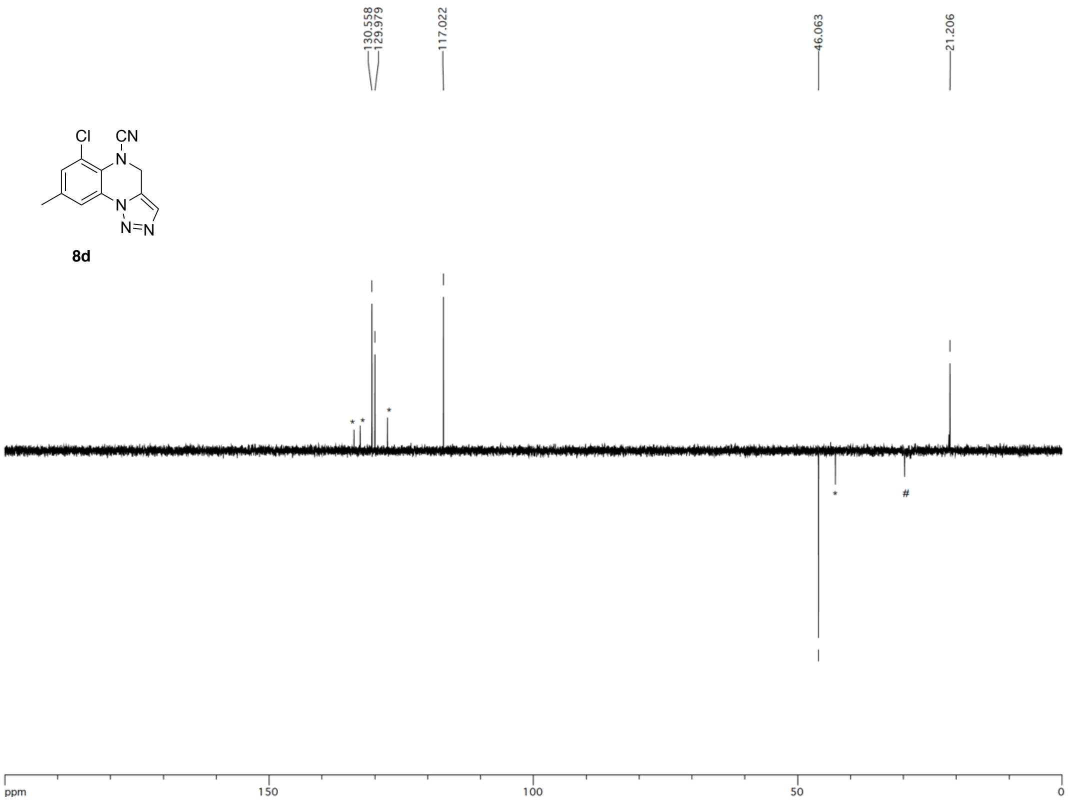

^{1}H NMR (400 MHz, DMSO-d_6, 298 K) Asterisks indicate solvent peaks:

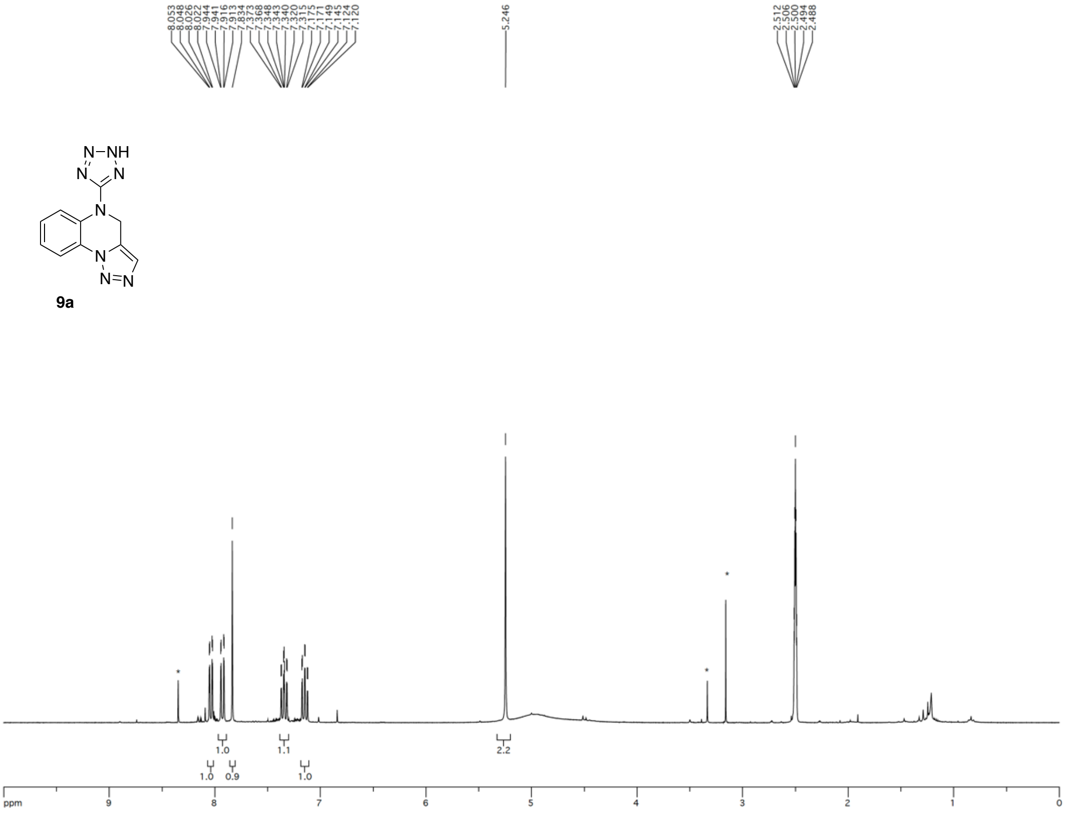

$^{13}C\{^{1}H\}$ NMR (100 MHz, DMSO-d_6, 298 K) Asterisks indicate solvent peaks:

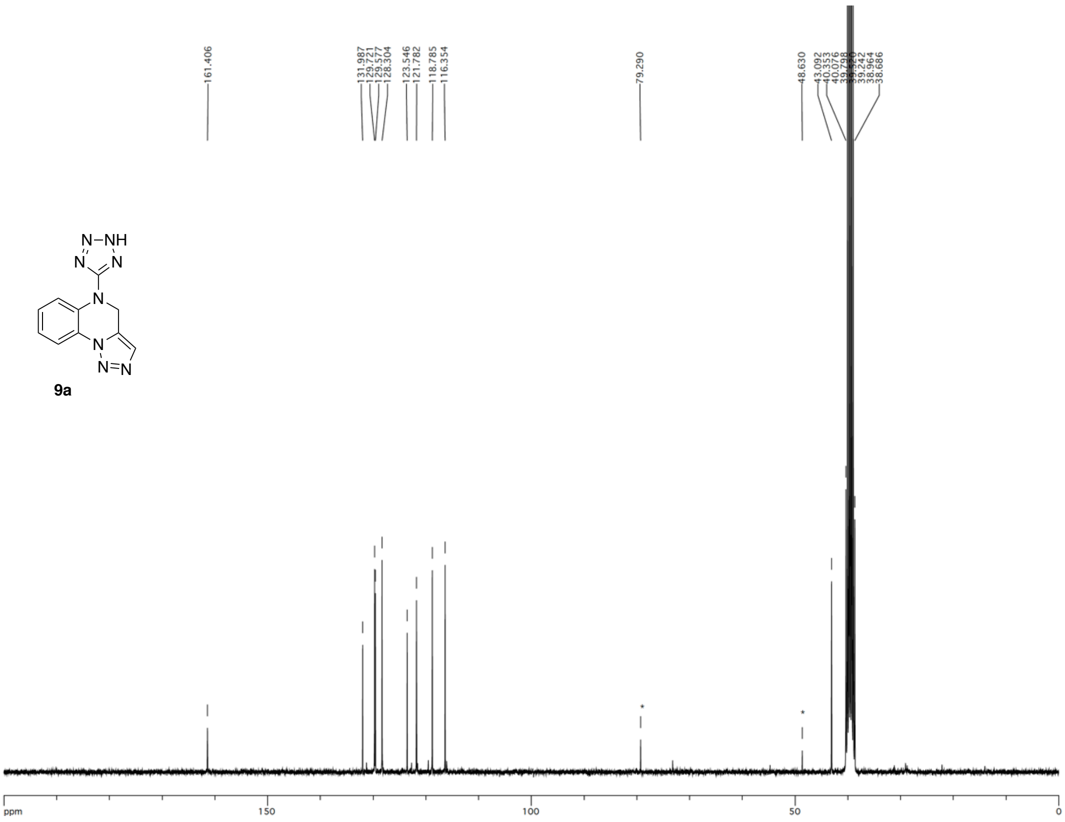

Dept-135 (100 MHz, DMSO-d_6, 298 K):

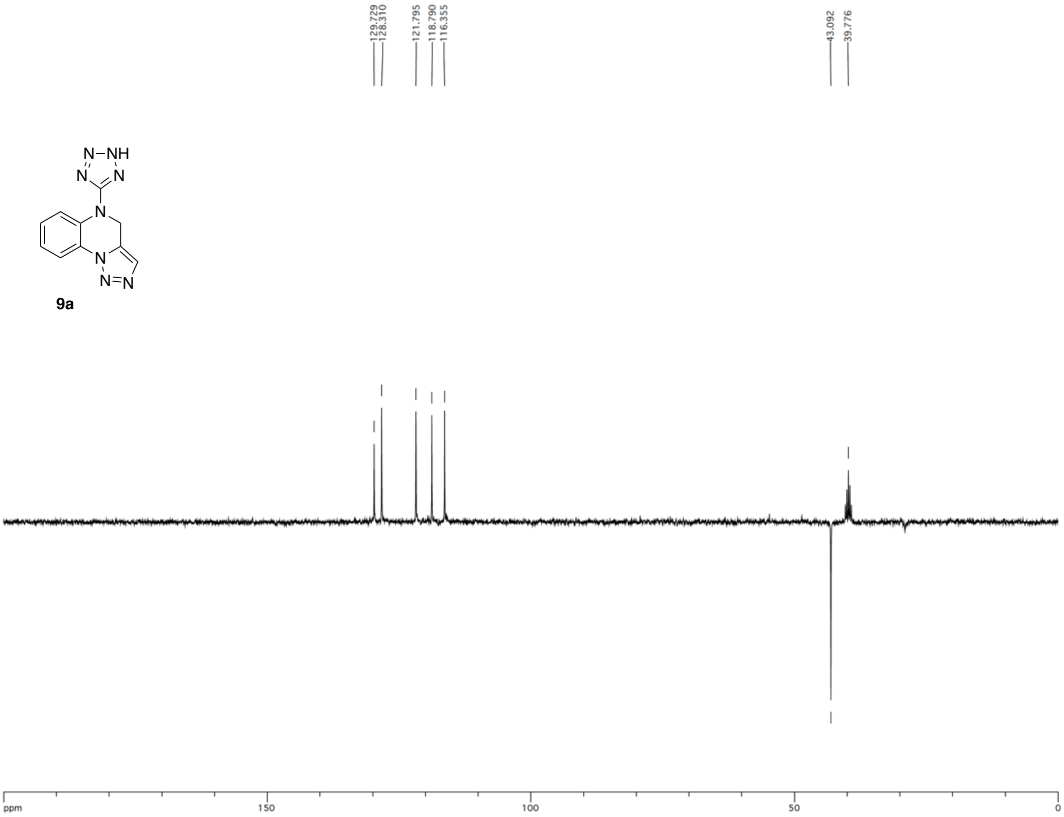

^{1}H NMR (400 MHz, DMSO-d_6, 298 K) Asterisks indicate solvent peaks:

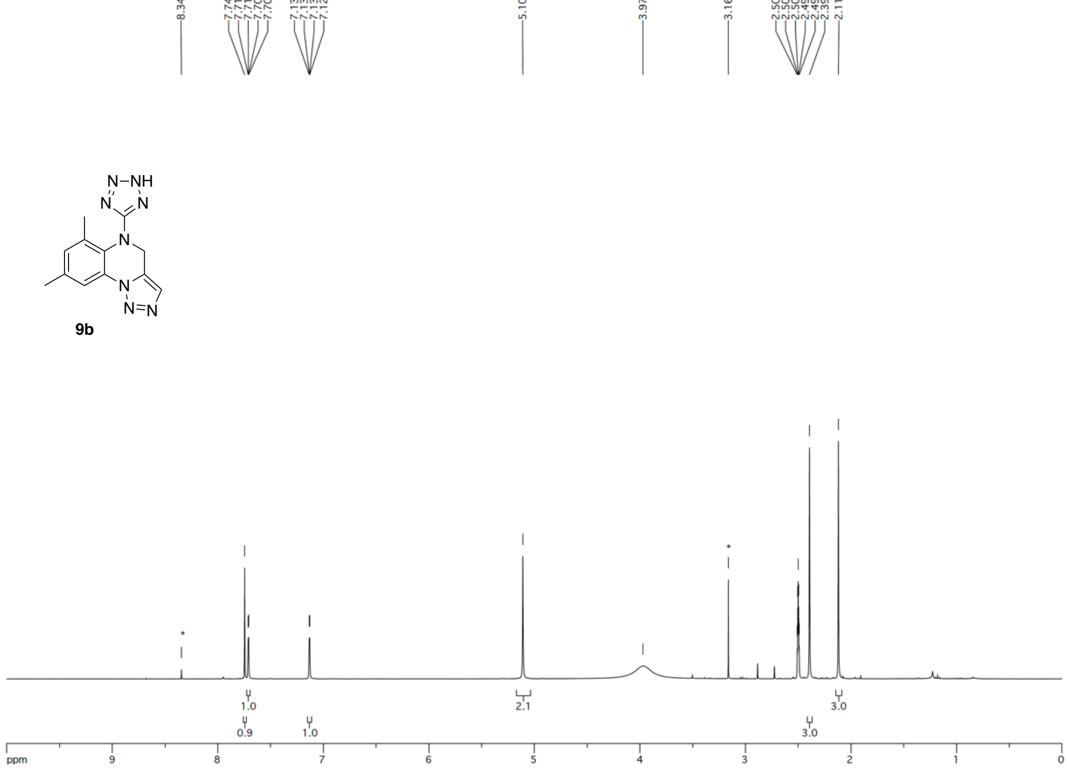

$^{13}C\{^{1}H\}$ NMR (100 MHz, DMSO-d_6, 298 K):

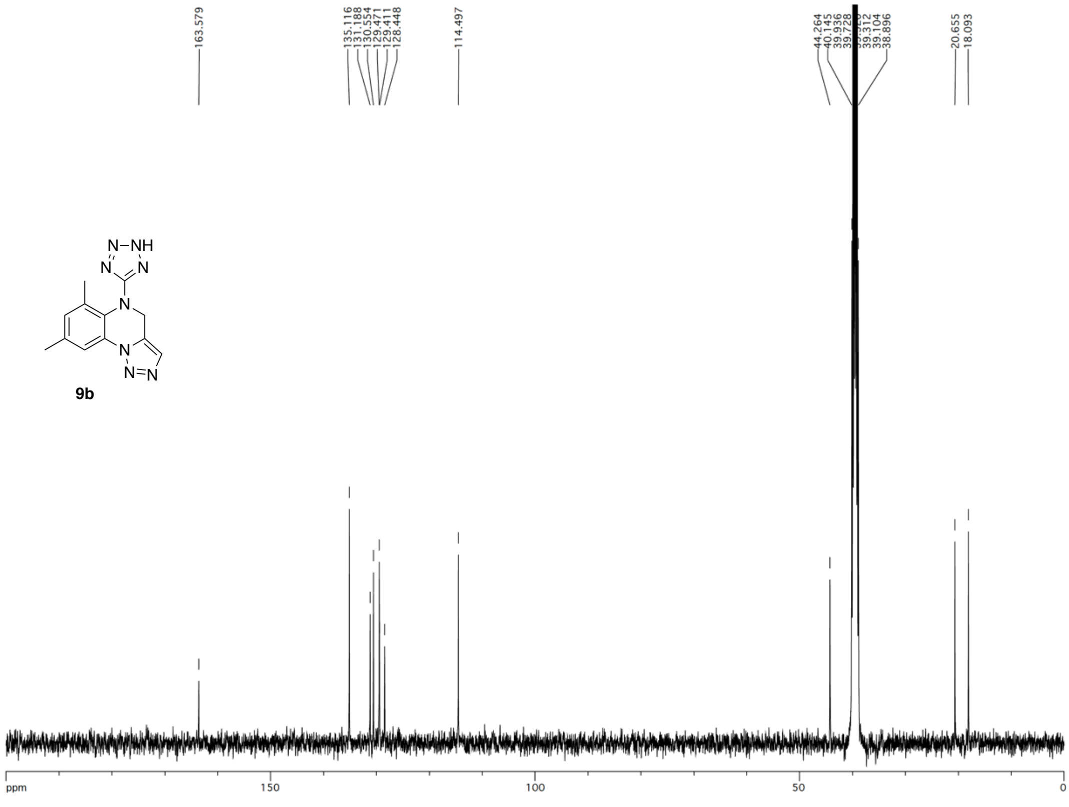

Dept-135 (100 MHz, DMSO-d_6, 298 K):

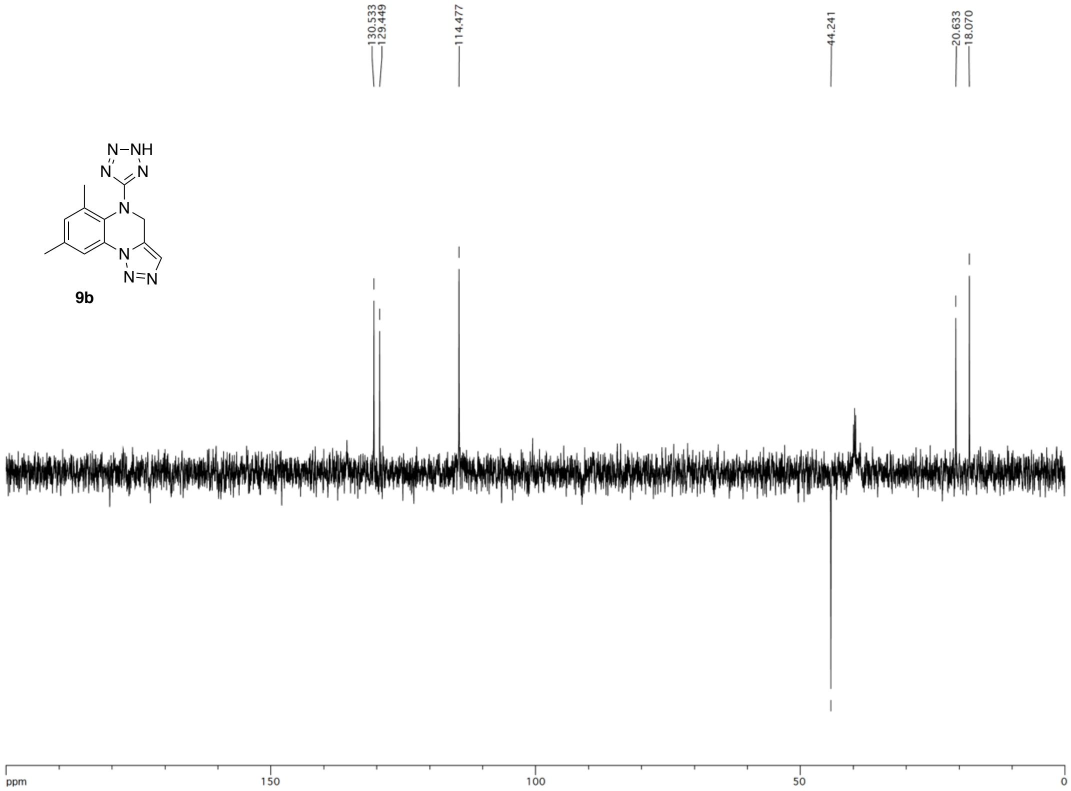

HMBC (400 MHz, DMSO-d_6, 298 K):

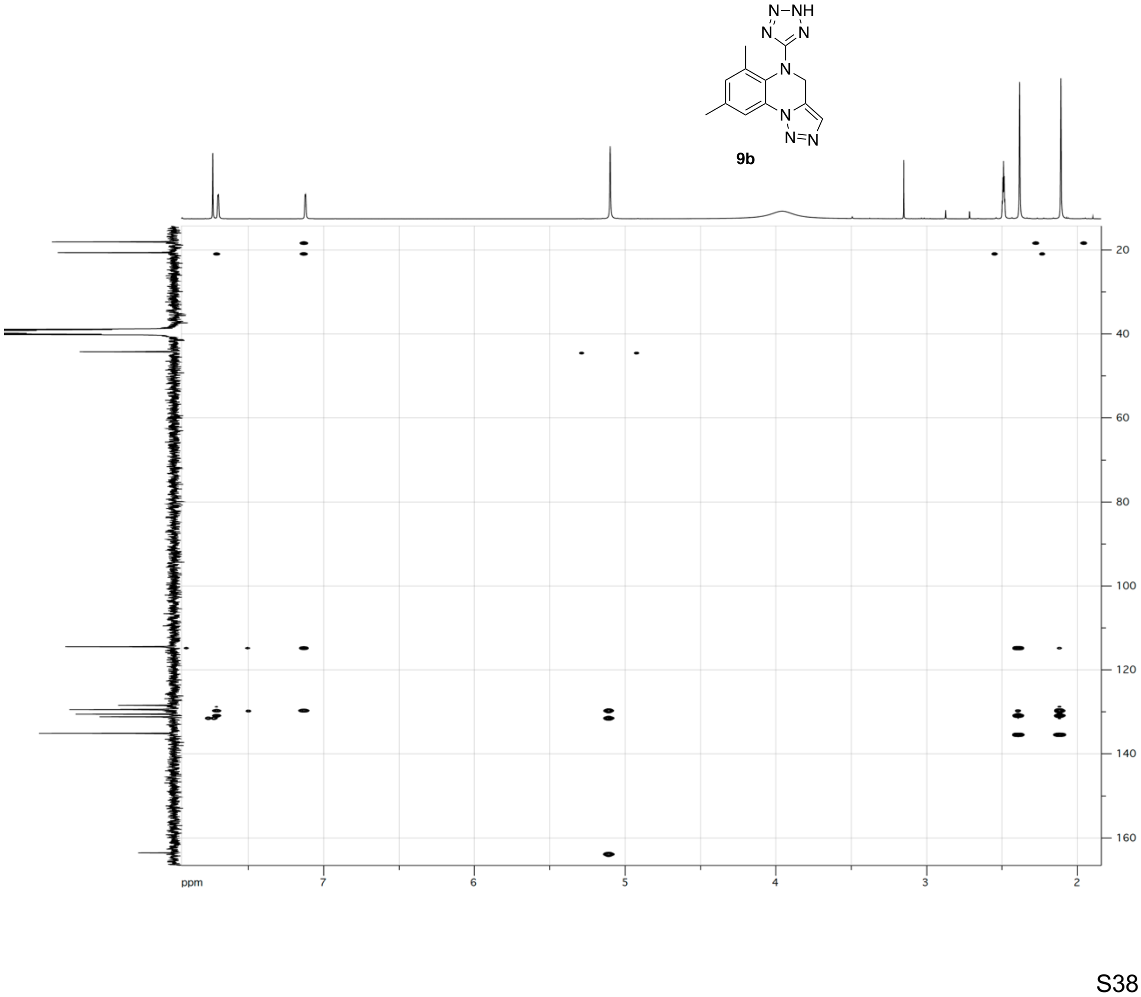

HSQC (400 MHz, DMSO-d_6, 298 K):

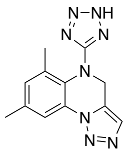

9b

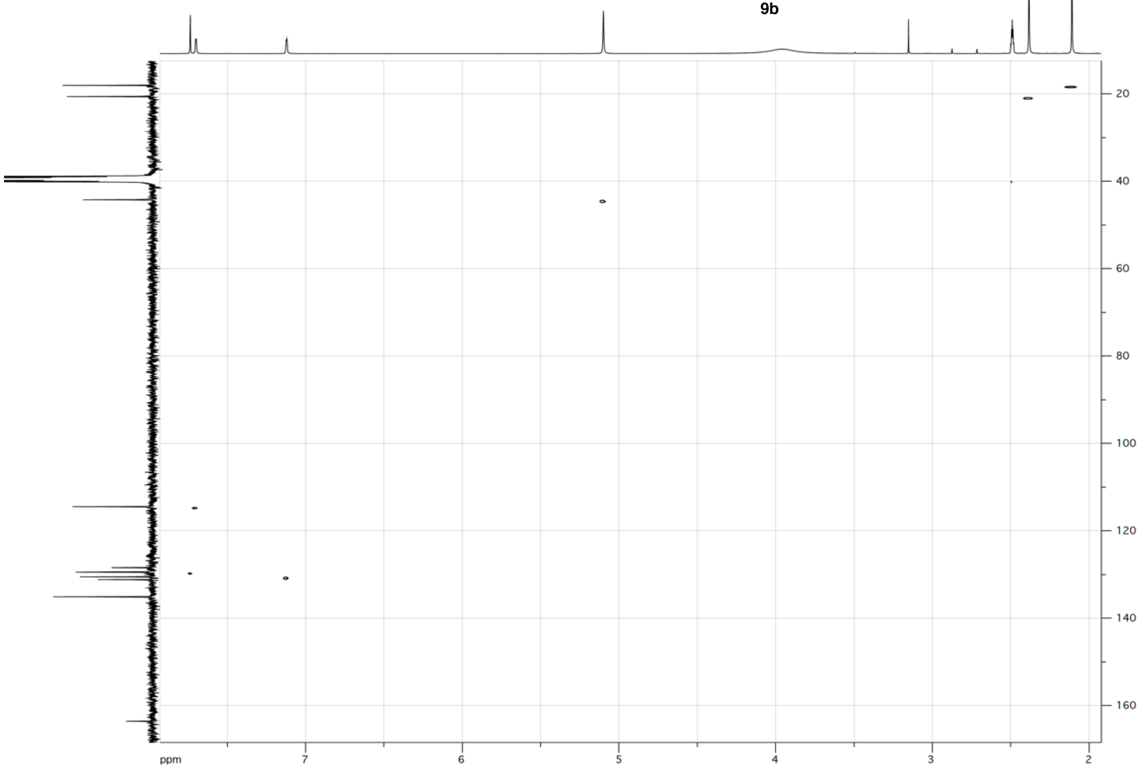

NOESY (400 MHz, DMSO-d_6, 298 K):

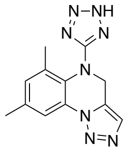

9b

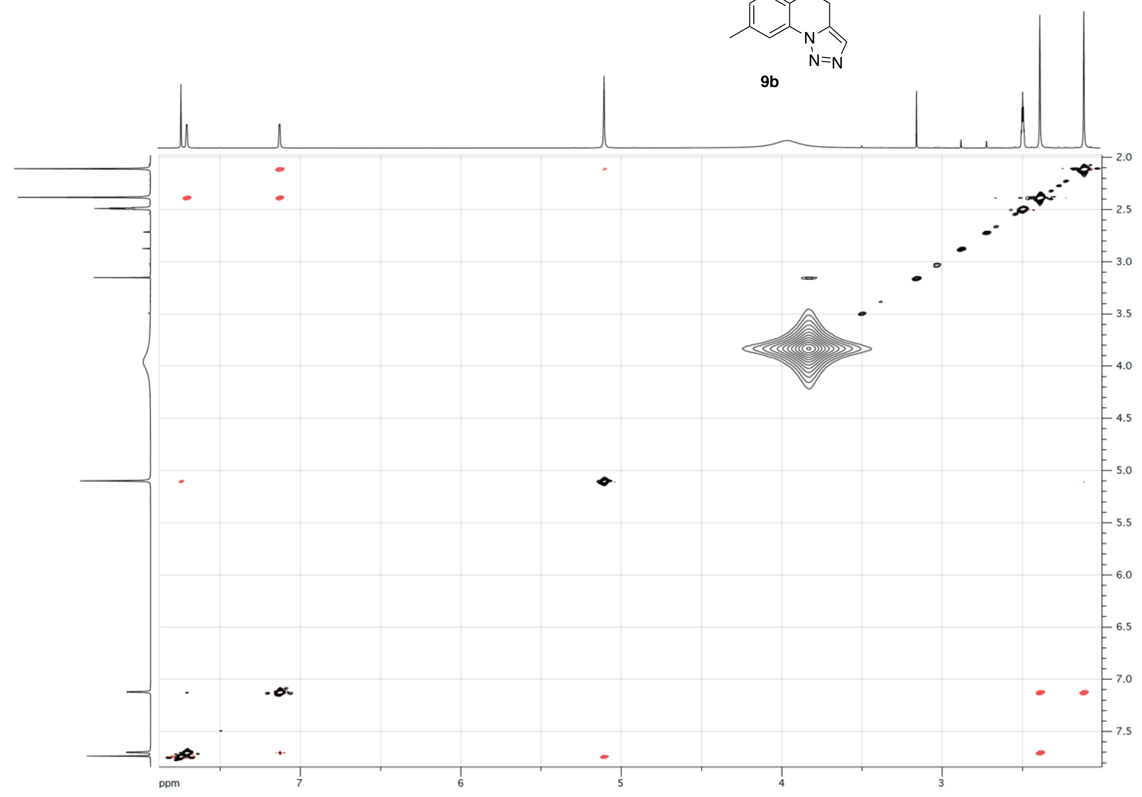

^{1}H NMR (400 MHz, DMSO-d_6, 298 K):

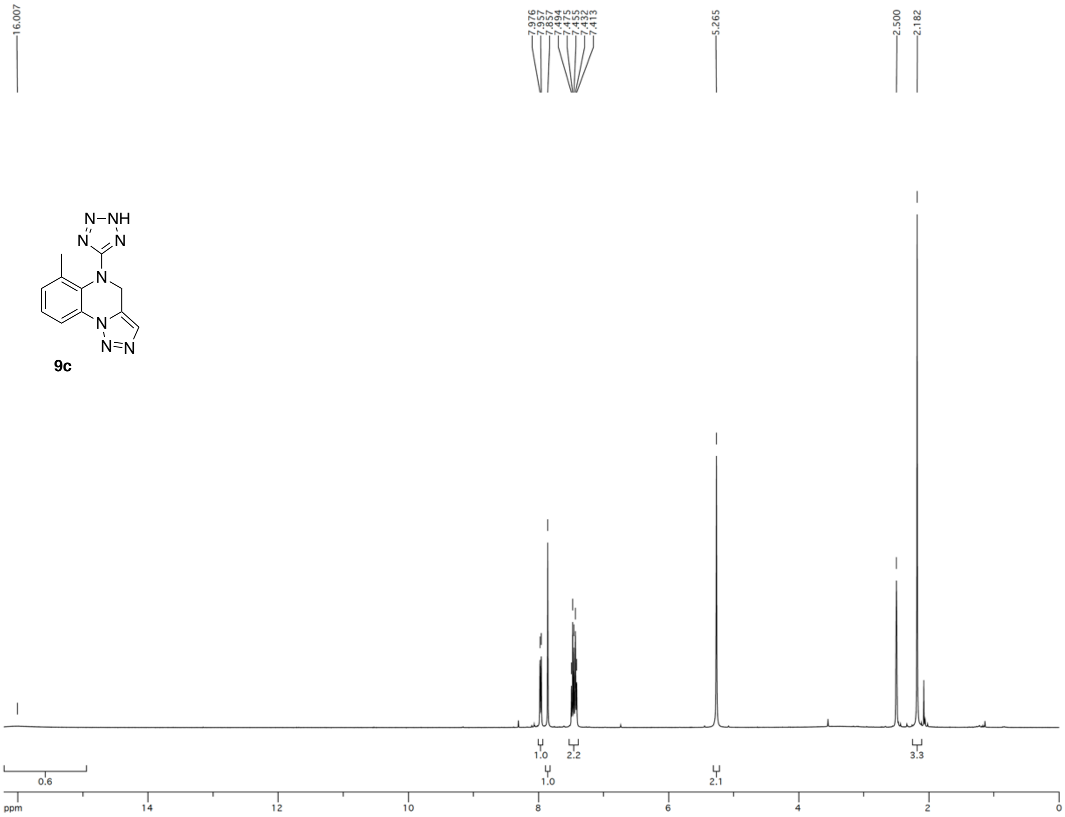

^{13}C{^{1}H} NMR (100 MHz, DMSO-d_6, 298 K):

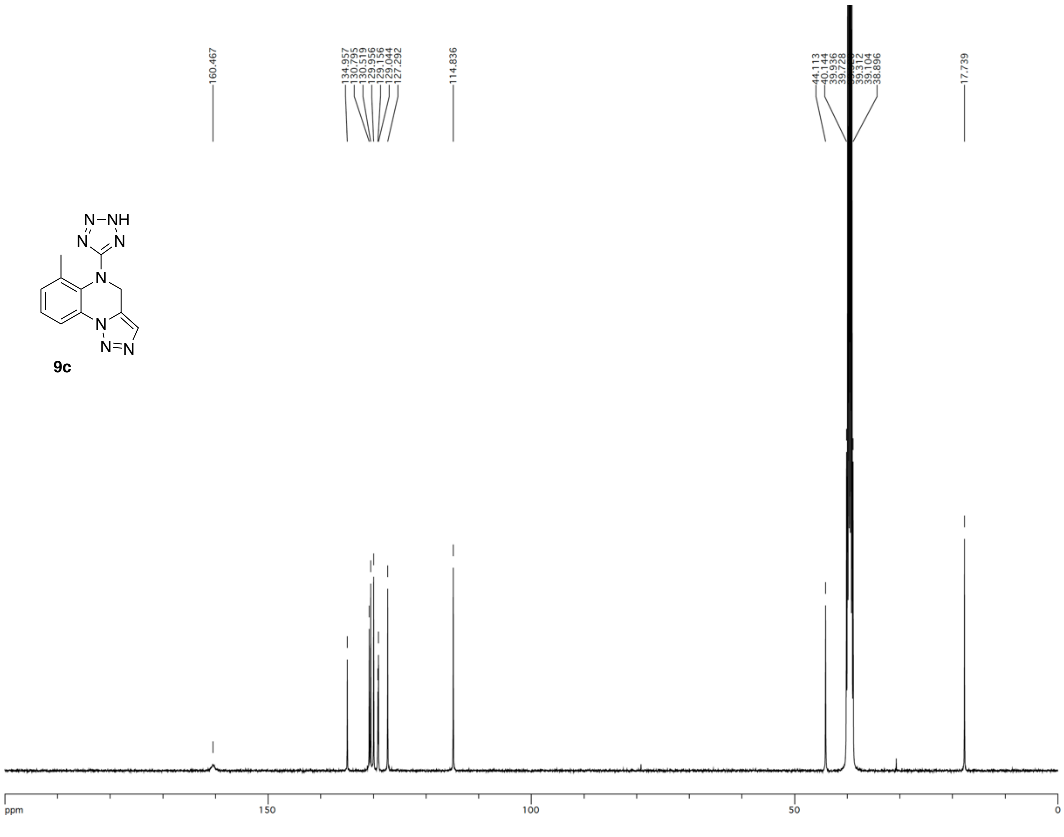

Dept-135 (100 MHz, DMSO-d_6, 298 K):

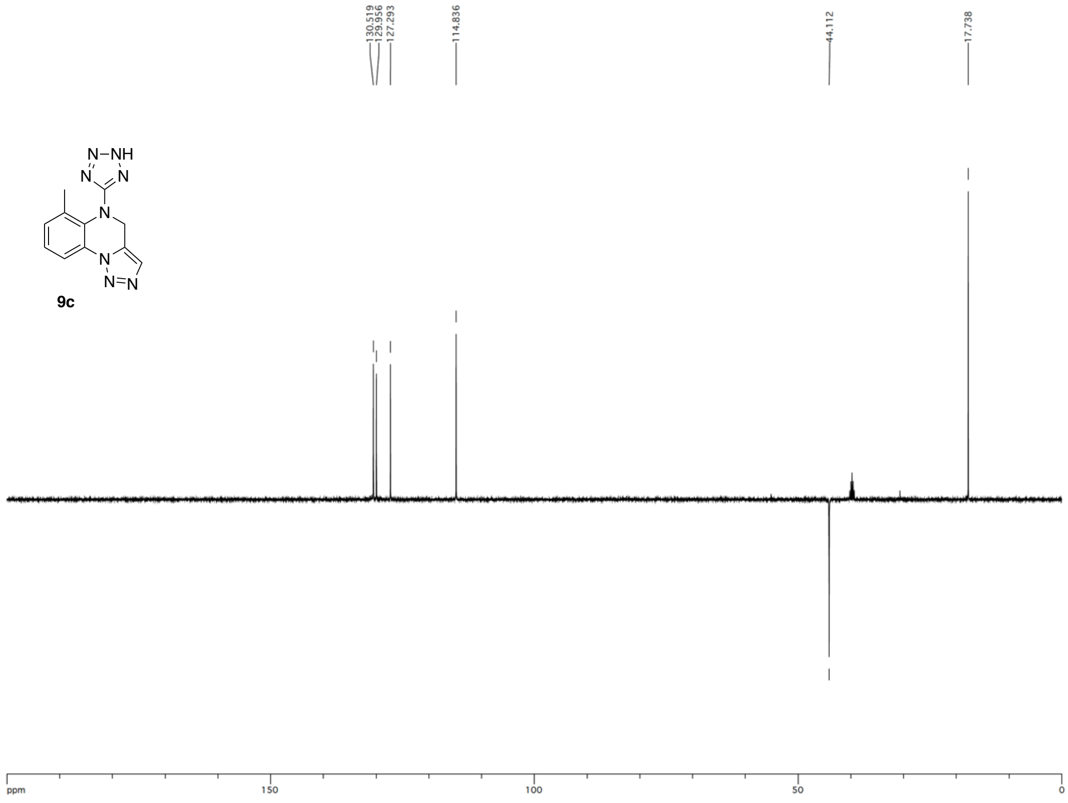

^{1}H NMR (400 MHz, DMSO-d_6, 298 K) Asterisks indicate solvent peaks:

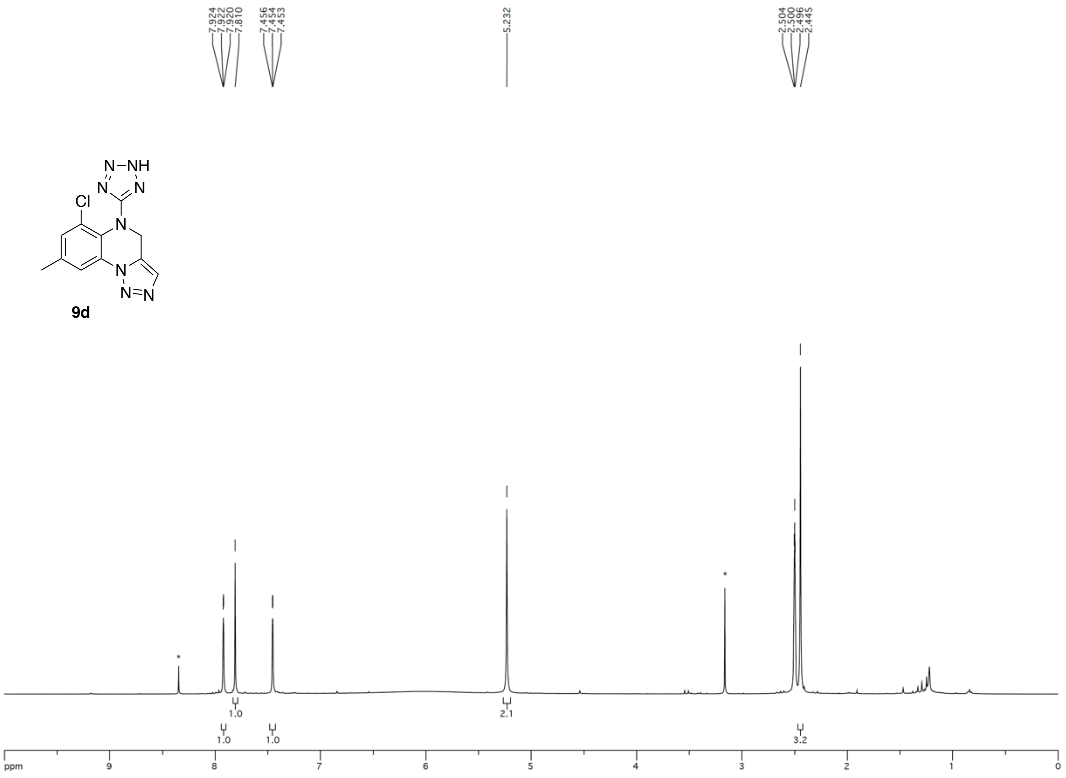

$^{13}C\{^{1}H\}$ NMR (100 MHz, DMSO-d_6, 298 K):

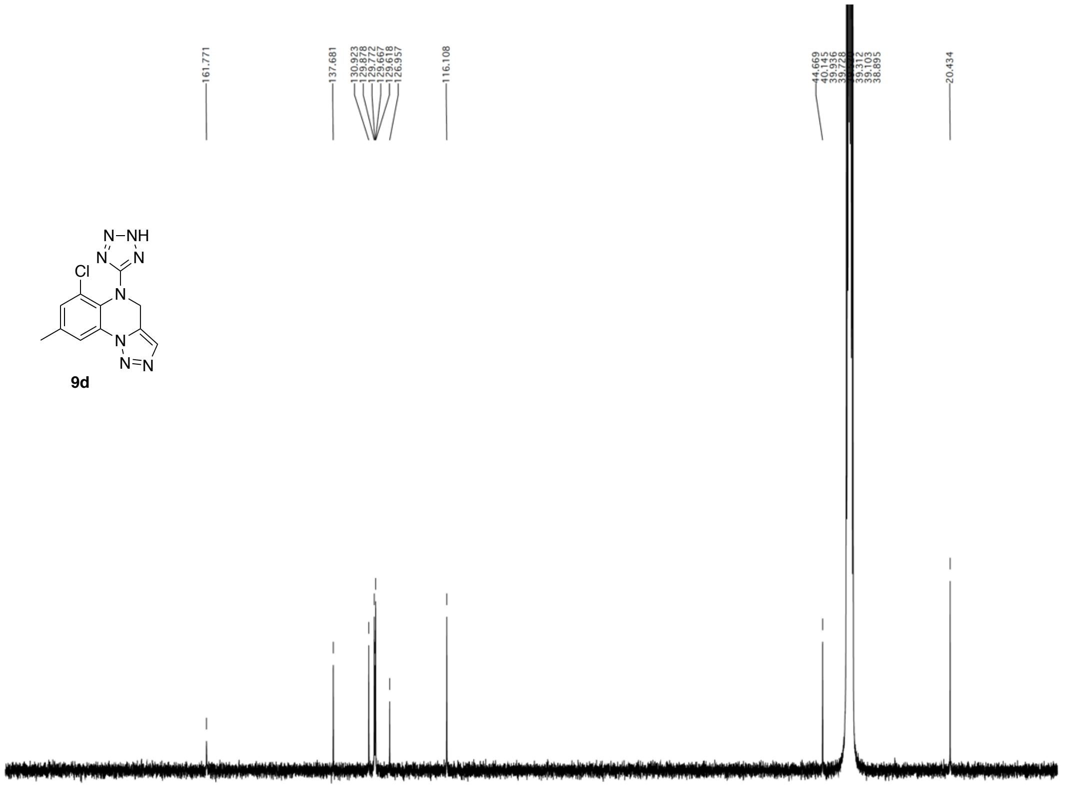

Dept-135 (100 MHz, DMSO-d_6, 298 K):

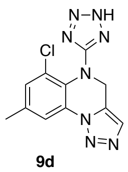

9d

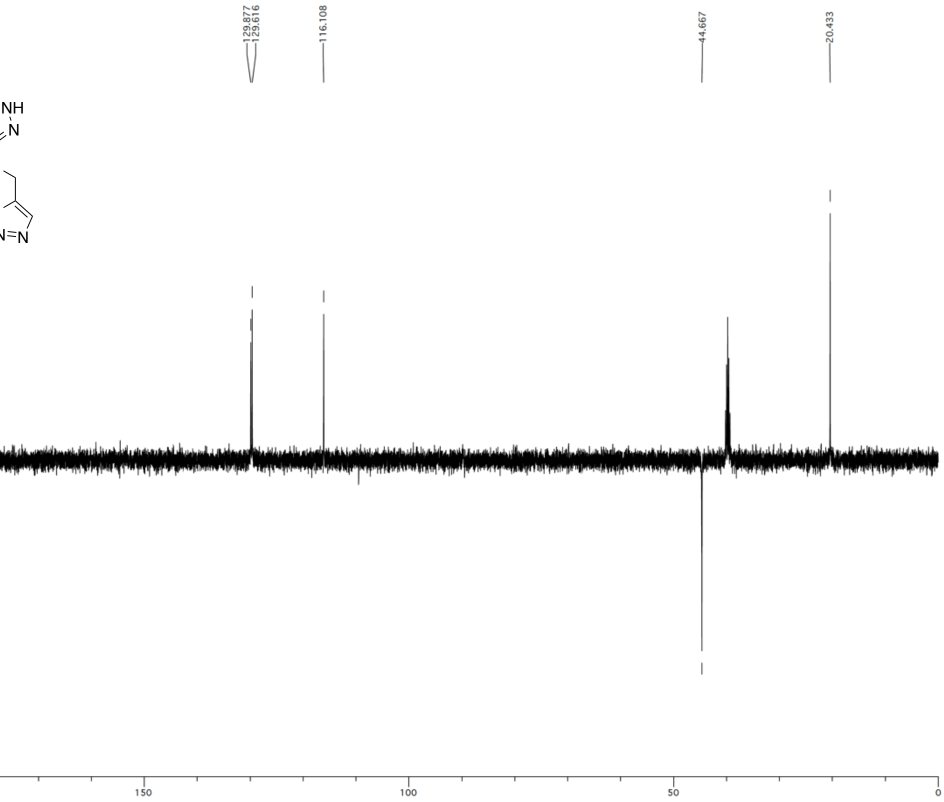

^{1}H NMR (600 MHz, $CDCl_3$, 298 K):

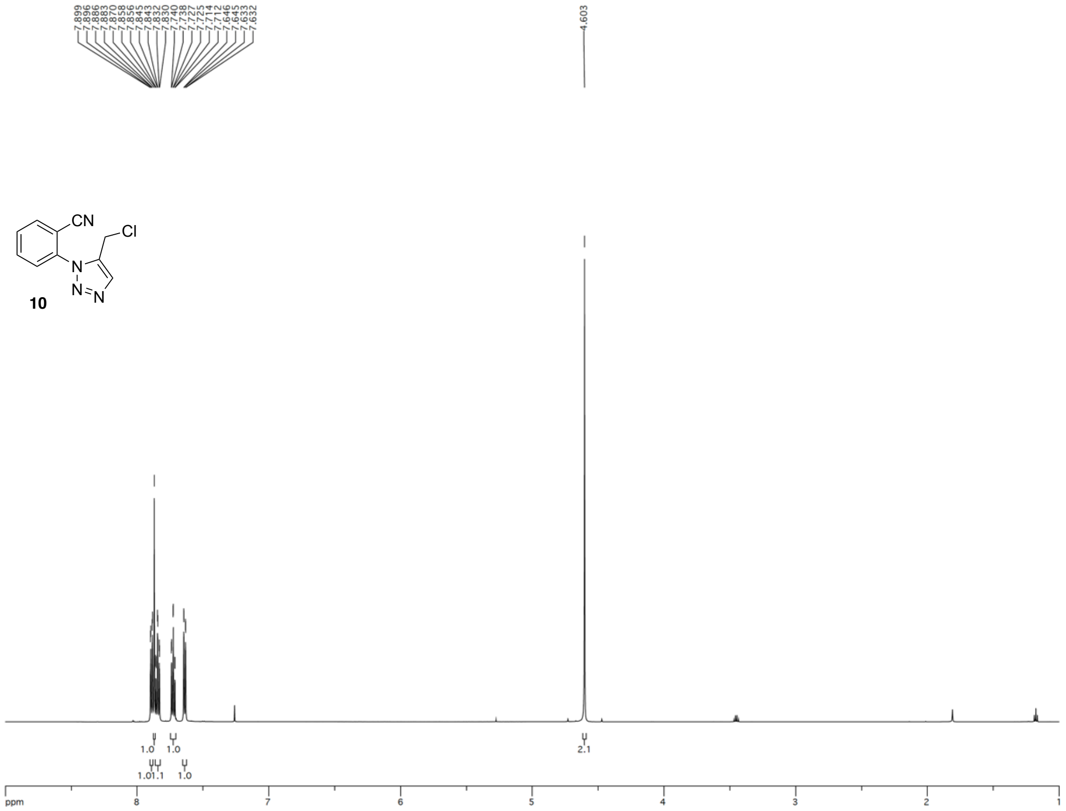

$^{13}C\{^{1}H\}$ NMR (150 MHz, $CDCl_3$, 298 K):

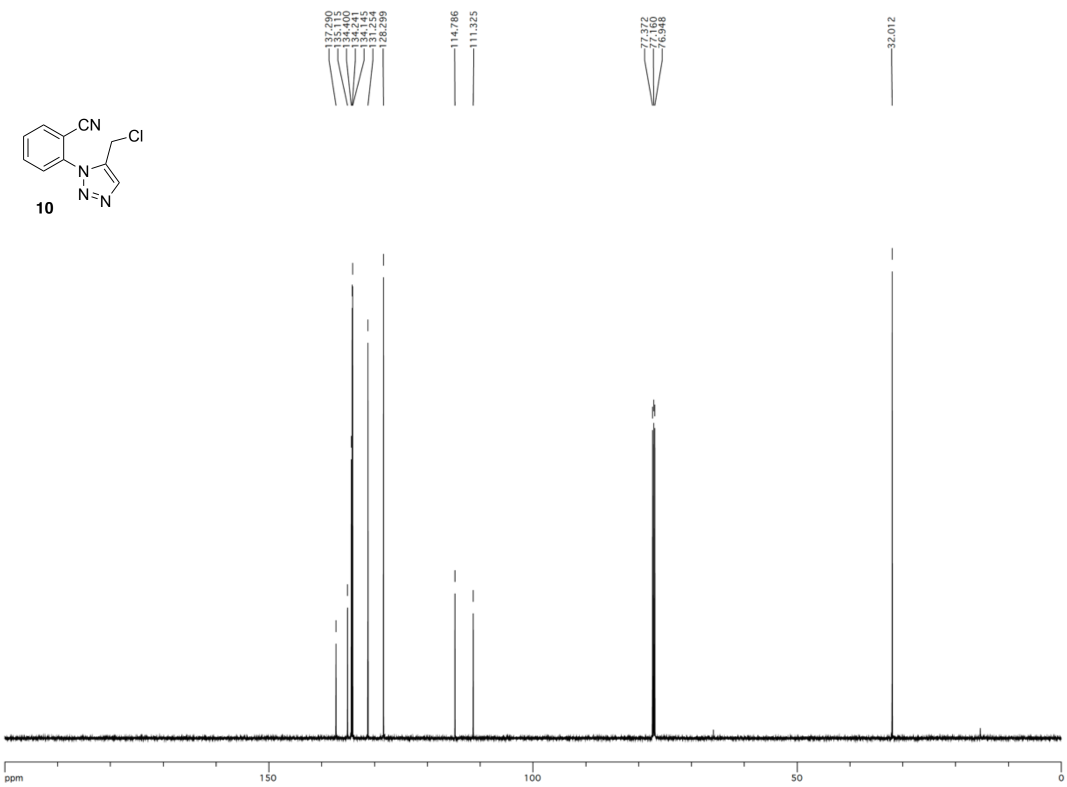

Dept-135 (150 MHz, $CDCl_3$, 298 K):

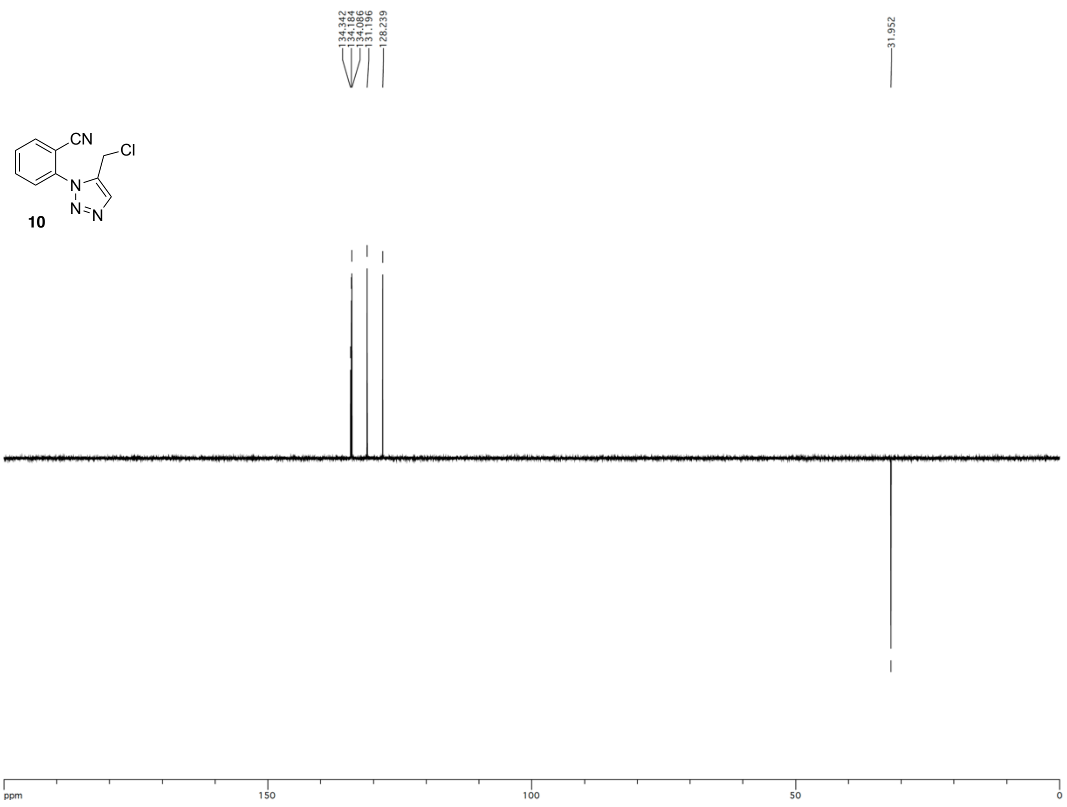

^{1}H NMR (400 MHz, $CDCl_3$, 298 K):

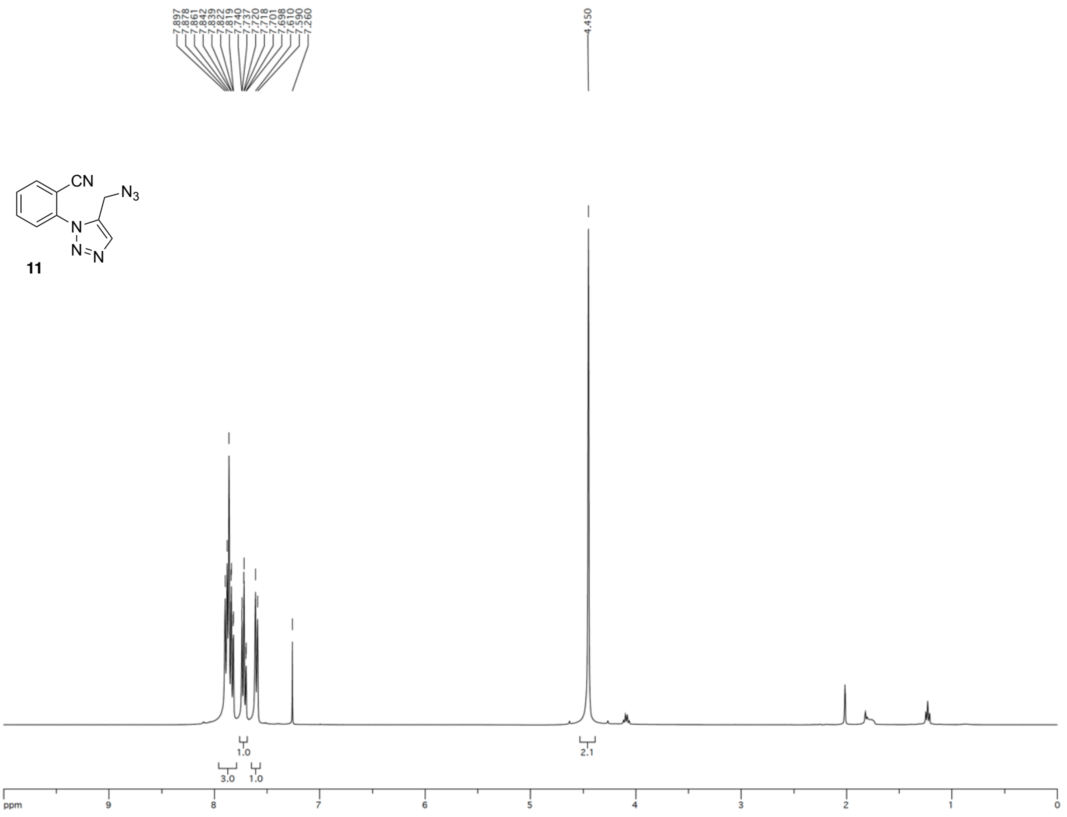

11

^{13}C{^{1}H} NMR (100 MHz, $CDCl_3$, 298 K):

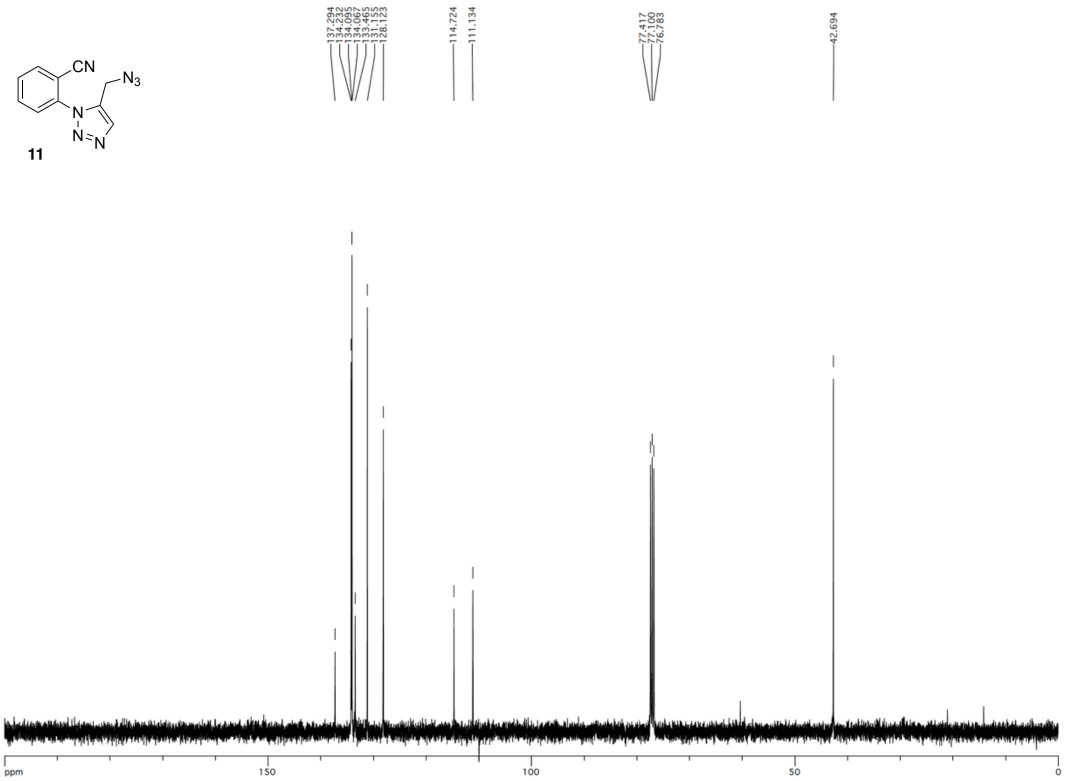

11

Dept-135 (100 MHz, CDCl₃, 298 K):

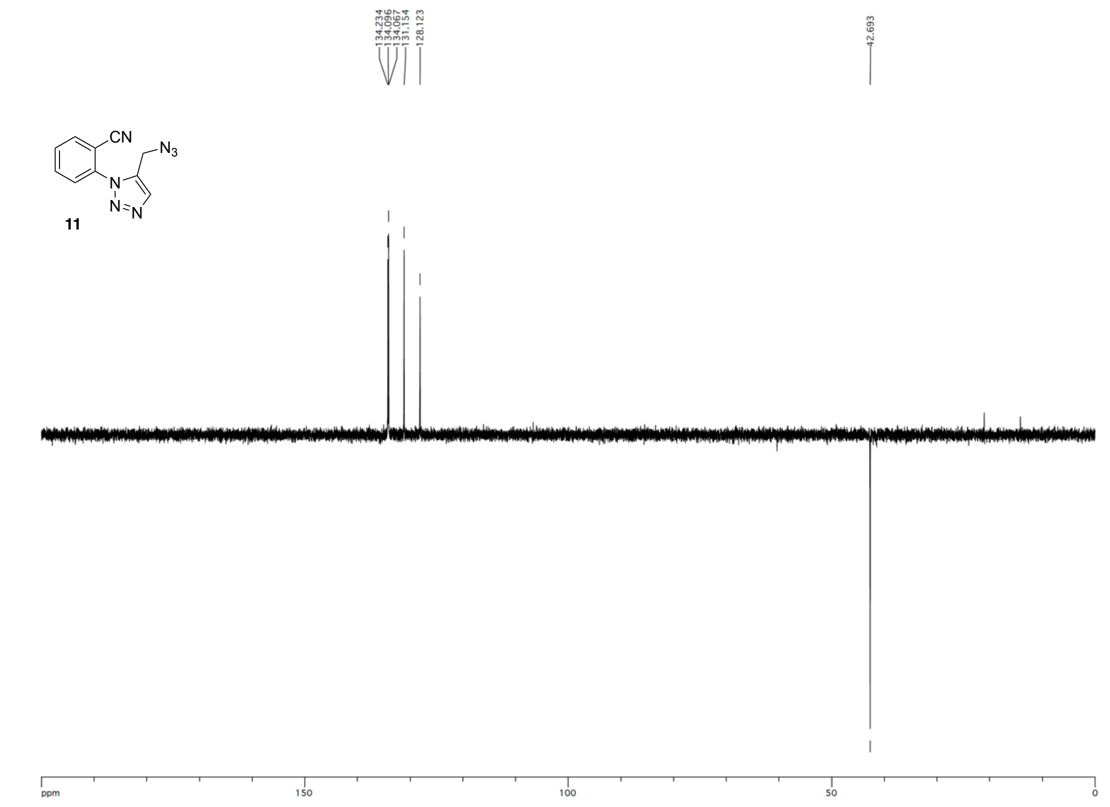

COSY (400 MHz, CDCl₃, 298 K):

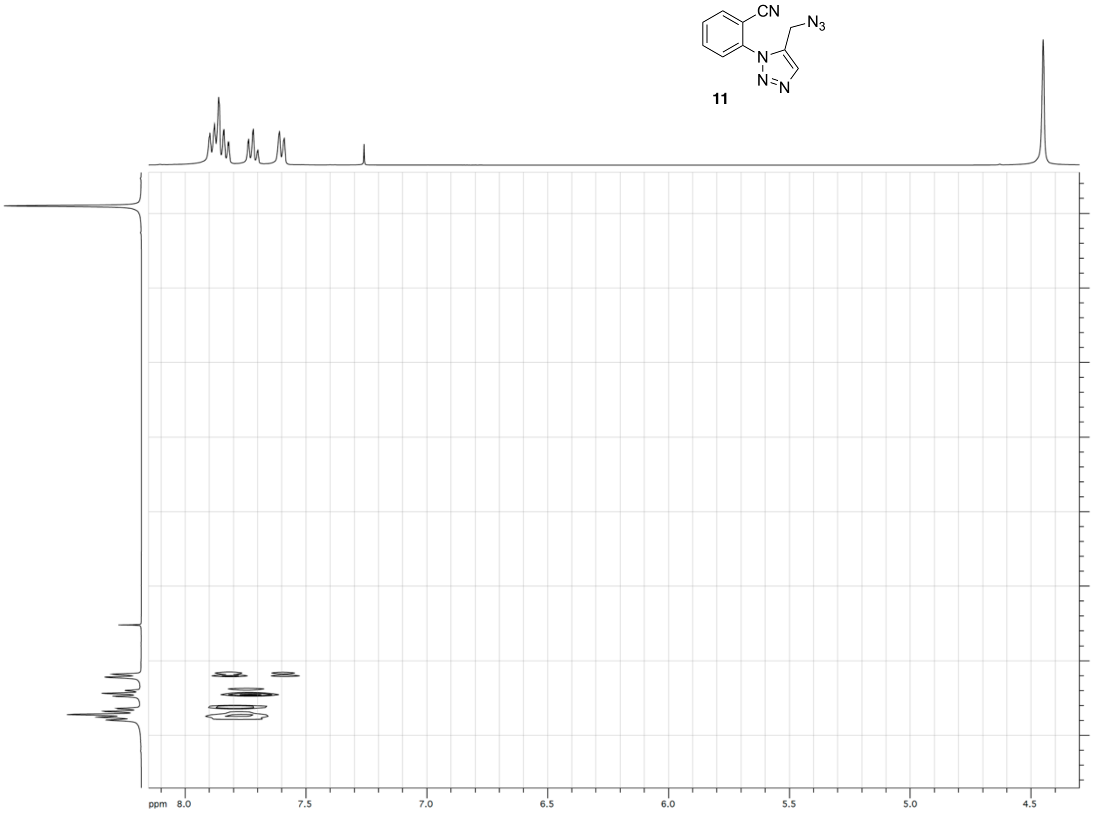

HSQC (400 MHz, $CDCl_3$, 298 K):

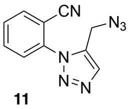

11

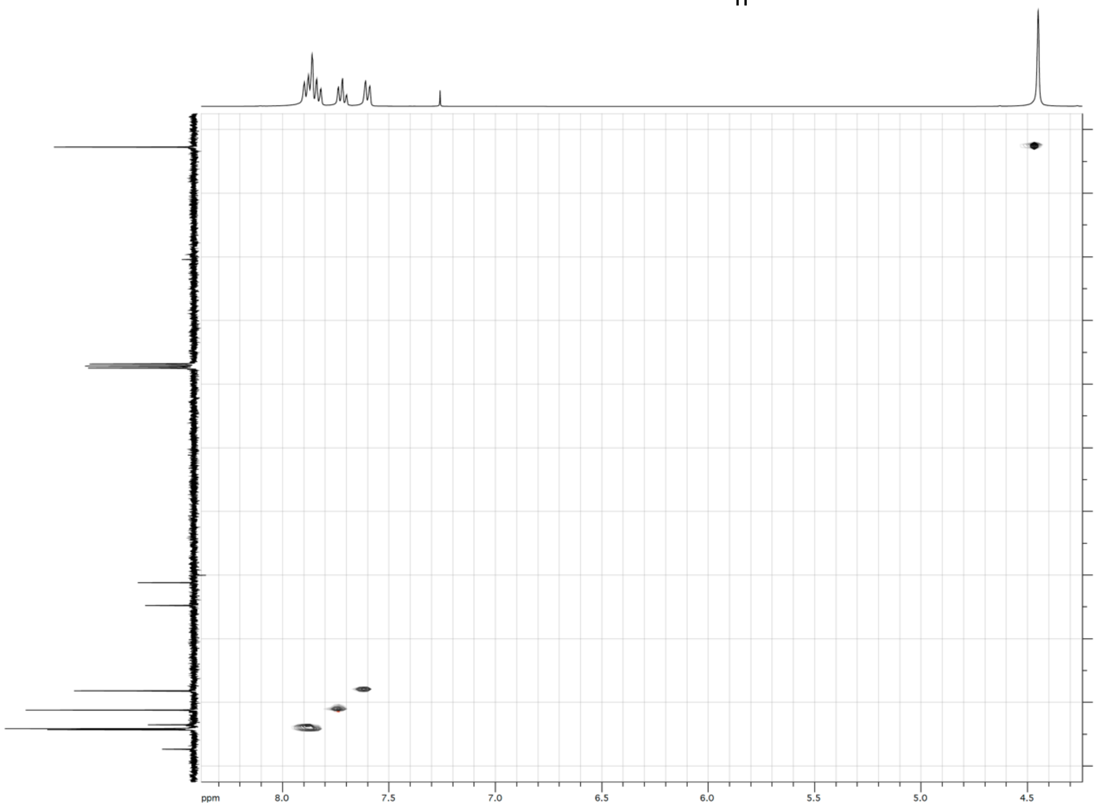

^{1}H NMR (400 MHz, DMSO-d_6, 298 K):

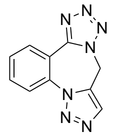

12

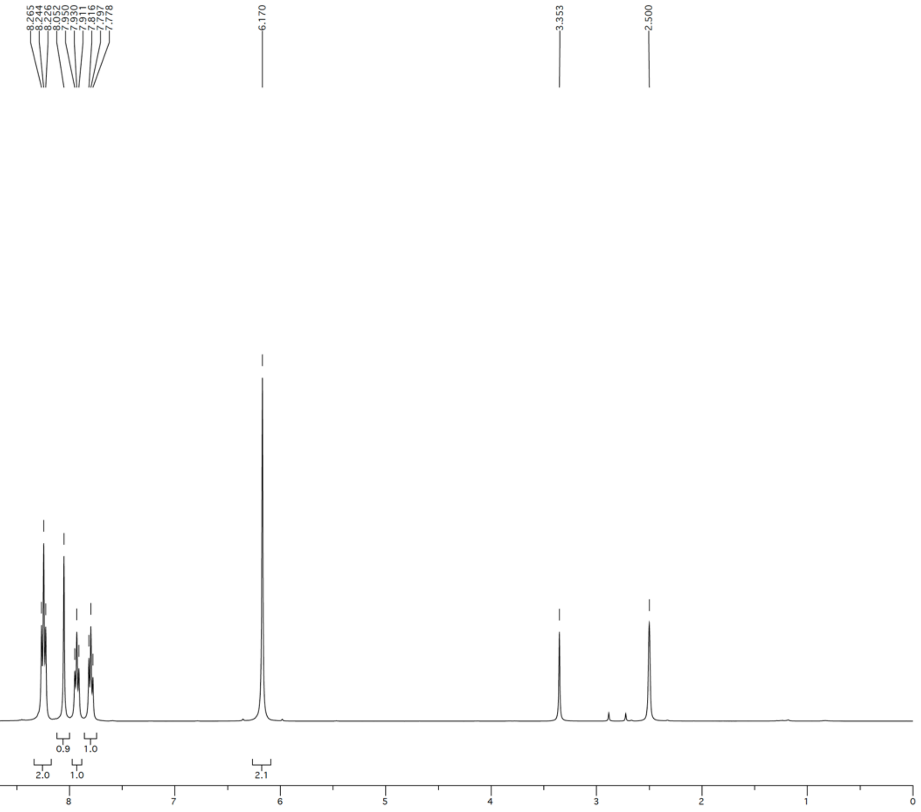

$^{13}C\{^{1}H\}$ NMR (100 MHz, DMSO-d_6, 298 K):

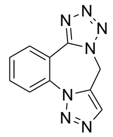

12

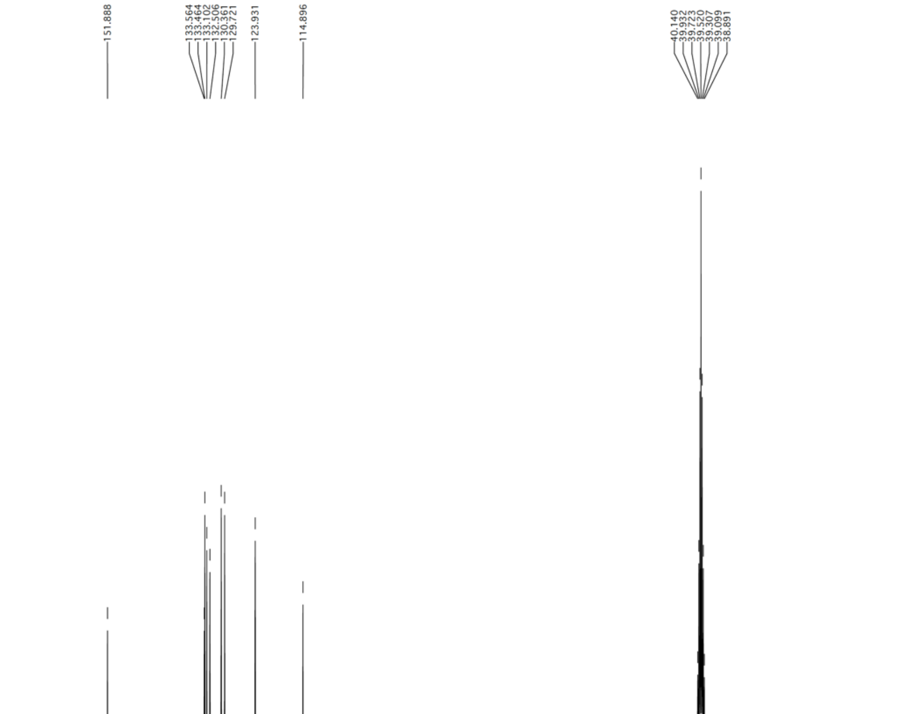

Dept-135 (100 MHz, DMSO-d_6, 298 K):

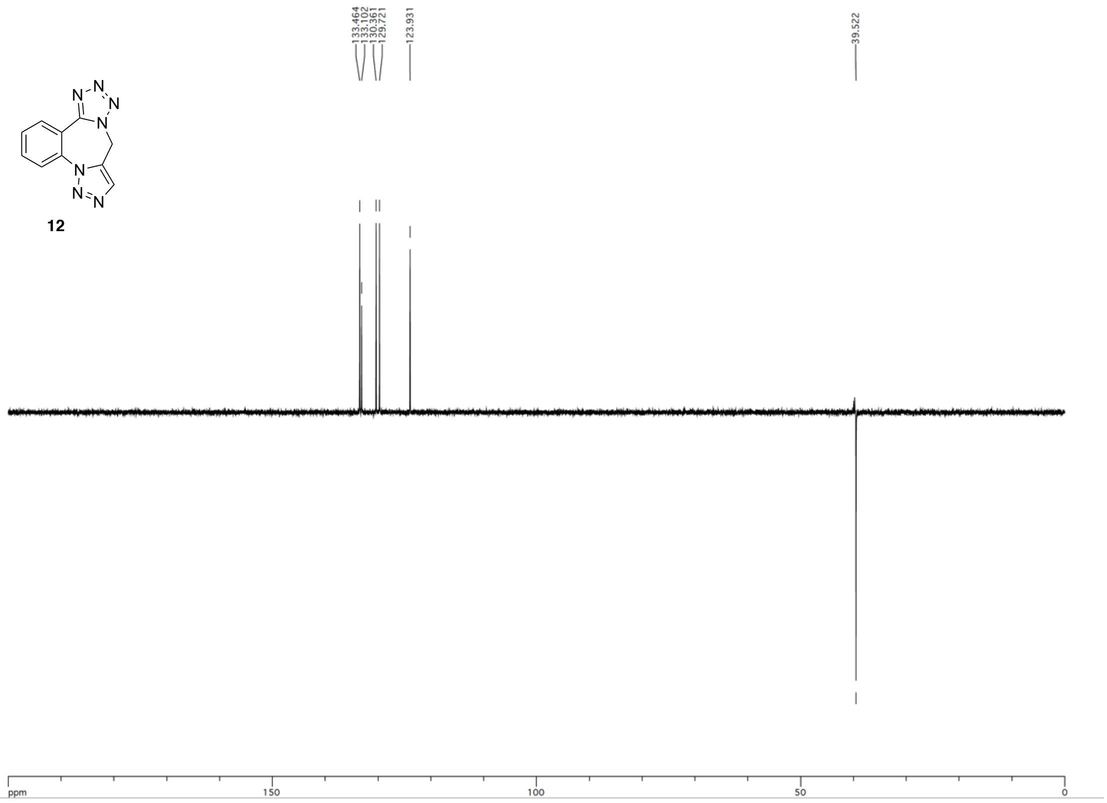

5. Computational Study

5.1. Computational Methods

Geometries of the molecules were optimized by using the wB97XD-functional[2] with the 6-31G+(d,p) basis sets.[3,4] Solvent effects were calculated with the PCM continuum solvation model for dimethylformamide.[5] The nature of minimum and transition structures of all found stationary points on the potential energy surface was confirmed by frequency analysis at the same level of theory. The stability of the resulting wavefunctions were checked for all the optimized structures.[6] The computed thermochemical corrections at PCM(DMF)/wB97XD/6-31+G(d,p) level were combined with single point energies at the PCM(DMF)/wB97xD/6-311++G(d,p)//PCM(DMF)-wB97XD/6-31+G(d,p) level to yield Gibbs free energies G at 298.15 K ($G_{298,sol}$). The ultrafine grid implemented in Gaussian 09 D. 01 was used.[7]

5.2. Alternative mechanism initiated by an intramolecular [3+1] cycloaddition of the azido-isocyanide 1b

The azido-isocyanide moieties of **1b** could react each other by forming either a new C-N3 (**TSa**) or a C-N1 bond (**TSb**). In the first scenario, the transition structure **TSa** (211.9 kJ mol^{-1}) conducted to the intermediate **INT1** which converts into **3b** by the formation of the C-N1 bond (**TSc**, +93.7 kJ mol^{-1}). In the second case, the transition structure **TSb** (181.5 kJmol^{-1}) indicated that evolves to the intermediate **3b** as the IRC analysis executed over **TSb** evidenced. So, the route via **TSb** is the most energetically favourable to achieve the triazolo-triazocine **3b**. After that, the departure of N_2 at the **TSd** (101.6 kJ mol^{-1}) provides the carbodiimide **4b**. Calculations predict that the initial formation of the C-N1 bond (**TSb**) if the rate determining step (RDS) of this mechanism. However, this step is almost 50 kJ mol^{-1} higher in energy, that is non-competitive, than the computed RDS for the proposed mechanism to achieve the cyanamide **8b** from **1b** with the participation of an external azide anion (see Figure 3 of the manuscript).

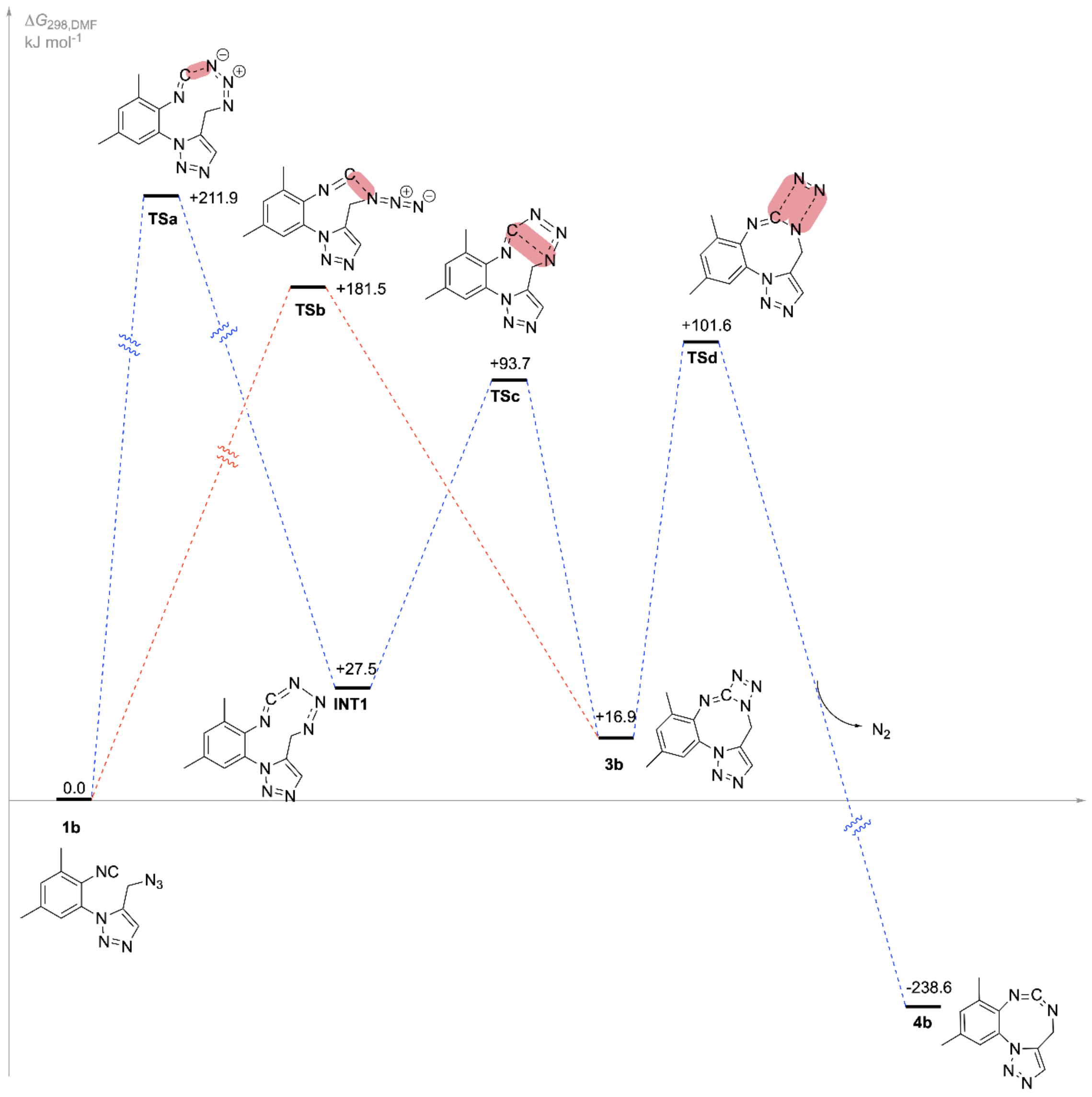

Figure S8. Computed mechanism to transform the azide-isocyanide **1b** into the carbodiimide **4b.**

Table S4. Value of imaginary frequencies, electronic, free energies and enthalpy (in Hartrees) of all stationary points found for the compounds shown Figure 3 and Figure S8. Free energies in solution at 298.15 K ($G_{298,sol}$) were calculated at the PCM(DCM)/M06/6-31+G(d,p) level and further combination with single point calculations at PCM(DMF)/wB97xD/6-311++G(d,p)//PCM(DMF)-wB97XD/6-31+G(d,p).

Stationary Point	Imagin. Freq.	PCM(DMF)/wB97XD/6-31+G(d,p)		PCM(DMF)/wB97xD/6-311++G(d,p) //PCM(DMF)/wB97XD/6-31+G(d,p)	
		$E_{SCF,298,sol}$	$G_{298,sol}$	$E_{SCF,298,sol}$	$G_{298,sol}$
**Azide anion**	-	-164.2740222	-164.2843940	-164.3127421	-164.3231139
**N2**	-	-109.4897669	-109.5025080	-109.5181342	-109.5308753
**1b**	-	-846.7873760	-846.6091260	-846.9507928	-846.7725428
**TS1**	-404.6	-1011.0337292	-1010.8455710	-1011.2335256	-1011.0453673
**TS'1**	-260.9	-1011.0322878	-1010.8470610	-1011.2348928	-1011.0496660
**13b**	-	-1011.0398273	-1010.8515290	-1011.2418815	-1011.0535832
**14b**	-	-1011.0907572	-1010.8984540	-1011.2882866	-1011.0959834
**TS2**	-709.0	-1011.0620306	-1010.8756190	-1011.2618923	-1011.0754807
**15b'E**	-	-901.6884664	-901.5048730	-901.8678859	-901.6842925
**15b'Z**		-901.6900774	-901.5053860	-901.8694469	-901.6847555
**TS3**	-707.4	-901.6342029	-901.4540870	-901.8130275	-901.6329116
**8b**	-	-737.4118651	-737.2333980	-737.5514160	-737.3729489
**TS'3**	-533.9	-901.6178056	-901.4366320	-901.7966698	-901.6154962
**4b**	-	-737.3717469	-737.1930280	-737.5113264	-737.3326075
**TSa**	-738.7	-846.7127382	-846.5314990	-846.8730898	-846.6918506
**TSb**	-477.7	-846.7234744	-846.5416640	-846.8852138	-846.7034034
**INT1**	-	-846.7848591	-846.5995830	-846.9473528	-846.7620767
**TSc**	-451.8	-846.7609774	-846.5758850	-846.9219482	-846.7368558
**3b**	-	-846.7961213	-846.6069410	-846.9553028	-846.7661225
**TSd**	-424.3	-846.7552921	-846.5718190	-846.9173266	-846.7338535

5.3. Cartesian Coordinates

Azide anion
SCF = -164.312742101
Num. Imaginary Freq = 0

N	0.000000	0.000000	1.180688
N	0.000000	0.000000	-0.000027
N	0.000000	0.000000	-1.180661

N2
SCF = -109.518134247
Num. Imaginary Freq = 0

N	0.000000	0.000000	-0.039790
N	0.000000	0.000000	-1.140898
N	0.000000	0.000000	0.550554
N	0.000000	0.000000	-0.550554

1b
SCF = -846.950792790
Num. Imaginary Freq = 0

N	-3.182726	2.584243	-0.929504
N	-2.476067	1.792418	-1.327460
N	-1.631812	1.028689	-1.801780
C	-1.861910	-0.404753	-1.509447
C	-1.927692	-0.702999	-0.047270
C	-2.949355	-1.146051	0.757975
N	-2.496634	-1.217740	2.037447
N	-1.254716	-0.837862	2.072527
N	-0.893323	-0.522130	0.816325
C	0.423926	-0.059437	0.533251
C	0.784127	1.235898	0.863027
C	2.073517	1.694066	0.580719
C	2.969262	0.822269	-0.042962
C	2.633238	-0.488338	-0.384913
C	1.337318	-0.922336	-0.080432
N	0.950506	-2.215318	-0.388789
C	0.636595	-3.314573	-0.654821
H	3.970841	1.173794	-0.274370
H	0.050127	1.883793	1.331068
H	-3.960490	-1.410011	0.488625
H	-1.028650	-0.926051	-1.981657
H	-2.785731	-0.751739	-1.980766
C	3.612776	-1.413747	-1.049267
H	3.792391	-2.299881	-0.432492
H	3.230308	-1.760239	-2.014385

H	4.566360	-0.910667	-1.215985
C	2.483559	3.092732	0.957326
H	2.728196	3.142855	2.023443
H	3.363603	3.412387	0.395243
H	1.674284	3.803268	0.770798

TS1
SCF = -1011.23352556
Num. Imaginary Freq = 1

N	4.290547	0.134022	-1.480945
N	3.506740	-0.675203	-1.600798
N	2.764619	-1.642820	-1.795477
C	1.379842	-1.464032	-1.331771
C	1.283683	-1.512807	0.162514
C	2.164848	-1.898271	1.146145
N	1.549524	-1.767848	2.351183
N	0.341104	-1.325619	2.168845
N	0.165915	-1.165659	0.843646
C	-1.065067	-0.664086	0.321381
C	-2.154024	-1.524354	0.215228
C	-3.364608	-1.055094	-0.285629
C	-3.437094	0.288273	-0.676056
C	-2.357978	1.163214	-0.578963
C	-1.138243	0.676144	-0.070297
N	-0.050404	1.523263	0.107081
C	0.895626	1.879767	-0.651613
H	-4.377136	0.667581	-1.070834
H	-2.040326	-2.558092	0.527476
H	3.179235	-2.254460	1.054526
H	0.813050	-2.286462	-1.771927
H	0.963609	-0.520034	-1.701653
C	-2.470884	2.603679	-0.997098
H	-2.191280	3.269185	-0.174131
H	-1.792888	2.824251	-1.827497
H	-3.490339	2.840372	-1.309137
C	-4.568584	-1.954775	-0.403375
H	-4.322921	-2.984328	-0.132537
H	-5.374978	-1.615190	0.254630
H	-4.959144	-1.954954	-1.425635
N	1.833167	2.886719	0.084271
N	1.315253	3.044587	1.273671
N	0.367538	2.609442	1.830511

TS'1
SCF = -1011.03228779
Num. Imaginary Freq = 1

N	-1.256481	0.076050	2.874325
N	-0.756784	1.028114	2.513421
N	-0.137295	2.059703	2.252543
C	-0.546894	2.758447	1.013899
C	0.326453	2.433317	-0.155165
C	1.355393	3.126941	-0.748259
N	1.814247	2.406834	-1.803870
N	1.126867	1.304964	-1.896405
N	0.222079	1.306704	-0.906134
C	-0.673269	0.208224	-0.725995
C	-2.007212	0.360441	-1.102159
C	-2.909426	-0.680285	-0.916089
C	-2.425891	-1.869804	-0.354620
C	-1.096497	-2.046513	0.015400
C	-0.194811	-0.979293	-0.165501
N	1.151578	-1.141670	0.147207
C	1.782515	-0.731236	1.129920
N	3.562706	-1.260333	0.865288
N	3.594080	-1.825111	-0.197067
N	3.463927	-2.339233	-1.226745
C	-0.610222	-3.334105	0.621033
H	-1.402687	-4.085574	0.628710
H	0.244302	-3.728262	0.061147
H	-0.271589	-3.178352	1.650442
H	-3.117960	-2.695243	-0.202358
C	-4.357327	-0.550237	-1.315780
H	-4.604656	-1.247960	-2.122428
H	-5.018745	-0.776378	-0.473732
H	-4.583317	0.460698	-1.663420
H	-2.324666	1.301719	-1.541914
H	1.774322	4.084502	-0.478867
H	-0.449953	3.823513	1.223258
H	-1.596396	2.553805	0.781097

13b*E*
SCF = -1011.24188154
Num. Imaginary Freq = 0

N	0.757548	0.613832	2.769408
N	0.218439	-0.350858	2.515546
N	-0.475381	-1.353806	2.344858
C	0.114852	-2.405480	1.490335
C	-0.363687	-2.361318	0.073936
C	-1.216534	-3.190302	-0.617311
N	-1.293547	-2.761108	-1.902772
N	-0.536063	-1.710054	-2.042712
N	0.030616	-1.454017	-0.856200
C	0.917227	-0.343605	-0.683668
C	2.269549	-0.620578	-0.474891
C	3.173262	0.412956	-0.267036
C	2.674553	1.722353	-0.294150
C	1.332936	2.015168	-0.516108
C	0.410274	0.961676	-0.712137
N	-0.923193	1.240169	-1.021491
C	-1.790186	1.191397	-0.067579
N	-3.124852	1.517200	-0.665454
N	-4.050000	1.510516	0.134310
N	-4.951587	1.521805	0.830704
C	0.844072	3.438548	-0.534553
H	1.678606	4.139678	-0.455769
H	0.291862	3.647103	-1.456729
H	0.153336	3.627612	0.293138
H	3.365891	2.547929	-0.135283
C	4.638642	0.147516	-0.029270
H	4.853250	-0.924149	-0.036423
H	5.255729	0.620890	-0.799833
H	4.958935	0.549687	0.937400
H	2.602606	-1.655081	-0.480763
H	-1.758426	-4.054926	-0.265172
H	-0.203022	-3.355881	1.919270
H	1.207614	-2.368602	1.531373

13b*Z*
SCF = -1011.23876676
Num. Imaginary Freq = 0

N	1.723905	0.051161	2.657966
N	1.080971	-0.849692	2.411138
N	0.314355	-1.803883	2.277321
C	0.585243	-2.683791	1.120790
C	-0.321082	-2.426776	-0.040122
C	-1.332101	-3.183026	-0.586083
N	-1.816548	-2.537355	-1.677624
N	-1.161848	-1.421845	-1.834066
N	-0.255736	-1.339224	-0.850013
C	0.620802	-0.212920	-0.741262
C	1.967883	-0.403040	-1.051863
C	2.867741	0.648589	-0.935660

C	2.363973	1.887459	-0.517875
C	1.022457	2.095521	-0.212382
C	0.112149	1.018786	-0.311455
N	-1.252406	1.206413	-0.084835
C	-1.705182	1.090077	1.115143
N	-3.213928	1.339590	1.153566
N	-3.721549	1.566367	0.053391
N	-4.164401	1.768769	-0.974807
C	0.530929	3.449435	0.226152
H	1.327761	4.194033	0.157007
H	-0.311552	3.775664	-0.392813
H	0.169549	3.422684	1.258512
H	3.051520	2.726344	-0.427037
C	4.331592	0.476927	-1.255083
H	4.554215	-0.550786	-1.552999
H	4.637701	1.139207	-2.071387
H	4.953820	0.719675	-0.387558
H	2.298784	-1.382470	-1.387011
H	-1.719726	-4.136614	-0.261024
H	0.408424	-3.703460	1.463637
H	1.633371	-2.609818	0.814609

14b
SCF = -1011.28828655
Num. Imaginary Freq = 0

N	1.736599	-0.636806	2.671536
N	0.812062	-1.230222	2.390247
N	-0.119744	-1.991693	2.126743
C	-1.363042	-1.330858	1.663621
C	-1.613417	-1.594026	0.214238
C	-2.620413	-2.270414	-0.432932
N	-2.372165	-2.249662	-1.768088
N	-1.265712	-1.601066	-1.984407
N	-0.796808	-1.192869	-0.793621
C	0.421389	-0.453936	-0.707094
C	1.623453	-1.120594	-0.909478
C	2.827512	-0.424866	-0.813403
C	2.782698	0.940962	-0.520021
C	1.585386	1.627960	-0.318704
C	0.386614	0.909988	-0.415404
N	-0.857907	1.568032	-0.232782
C	-1.770522	1.936825	-1.183796
N	-2.724945	2.549395	-0.445440
N	-2.364985	2.529737	0.868137
N	-1.234894	1.945713	1.021824
C	1.571081	3.102896	-0.020626
H	0.862780	3.625033	-0.670837
H	2.562826	3.535510	-0.165248
H	1.260017	3.288335	1.011644
H	3.715994	1.494109	-0.451011
C	4.145012	-1.134124	-0.987123
H	4.888764	-0.478166	-1.446482
H	4.537632	-1.450300	-0.014730
H	4.037327	-2.025879	-1.609042
H	1.606913	-2.183084	-1.130459
H	-3.485870	-2.761701	-0.015295
H	-1.334690	-0.254793	1.862846
H	-2.186709	-1.761864	2.233916

TS2
SCF = -1011.26189225
Num. Imaginary Freq = 1

N	-1.811358	-0.508004	-2.685581
N	-0.874362	-1.102503	-2.451618
N	0.071958	-1.864663	-2.249688
C	1.312369	-1.220571	-1.756427
C	1.571769	-1.556915	-0.324687
C	2.582591	-2.267387	0.278893
N	2.342128	-2.318137	1.614592
N	1.236971	-1.679690	1.870308
N	0.761176	-1.209523	0.706706
C	-0.451354	-0.456311	0.664109
C	-1.645715	-1.107060	0.958885
C	-2.848464	-0.411330	0.895218
C	-2.808458	0.941512	0.537162
C	-1.622043	1.614483	0.256841
C	-0.409265	0.897924	0.312643
H	-3.742777	1.496221	0.487553
H	-1.621330	-2.160286	1.220489
H	3.444884	-2.736647	-0.169691
H	1.277010	-0.135562	-1.898716
H	2.134686	-1.616741	-2.353252
C	-1.626229	3.076797	-0.091628
H	-1.313198	3.232348	-1.128783
H	-2.622645	3.503534	0.041262
H	-0.920100	3.628162	0.536668
C	-4.162054	-1.086591	1.194284
H	-4.824783	-1.050568	0.323929
H	-4.016895	-2.134109	1.468115
H	-4.678744	-0.586901	2.019644
N	0.826501	1.509949	0.085639
C	1.325091	2.201947	-0.920631
N	2.495417	2.683979	-0.778509
N	2.889513	2.183395	0.807010
N	2.001264	1.562563	1.284689

15b'*E*
SCF = -901.867885892
Num. Imaginary Freq = 0

N	0.974703	-0.466757	2.816468
N	-0.129485	-0.492094	2.558995
N	-1.338540	-0.632061	2.372281
C	-1.975689	0.405735	1.526791
C	-2.346363	-0.125040	0.182352
C	-3.564918	-0.339333	-0.419066
N	-3.352078	-0.836289	-1.664359
N	-2.067038	-0.941708	-1.862367
N	-1.447160	-0.513855	-0.753095
C	-0.020428	-0.517714	-0.655333
C	0.619862	-1.750482	-0.557933
C	2.001930	-1.812589	-0.442366
C	2.697448	-0.596746	-0.444385
C	2.078921	0.646039	-0.546716
C	0.660745	0.716248	-0.642137
N	-0.095821	1.862205	-0.777961
C	0.233374	3.020225	-0.306679
N	0.386405	4.122326	0.100912
C	2.923585	1.892793	-0.607395
H	3.981135	1.631136	-0.692034
H	2.654391	2.509147	-1.471532
H	2.799041	2.519570	0.280551
H	3.783647	-0.621484	-0.373746
C	2.731731	-3.125520	-0.308662
H	2.053180	-3.970915	-0.449625
H	3.535492	-3.210251	-1.047131
H	3.188189	-3.224183	0.682312
H	0.016939	-2.654253	-0.561501
H	-4.556674	-0.166795	-0.029216
H	-1.326195	1.279482	1.415220
H	-2.887244	0.714489	2.039750

15b'*Z*
SCF = -901.869446892

Num. Imaginary Freq = 0

N	1.576749	-1.323645	2.405085
N	0.478186	-1.500987	2.186833
N	-0.689618	-1.836443	1.985528
C	-1.631879	-0.727335	1.702711
C	-2.058222	-0.715566	0.272661
C	-3.253889	-1.012155	-0.337117
N	-3.114086	-0.831192	-1.675321
N	-1.896132	-0.439125	-1.924379
N	-1.246406	-0.359298	-0.753417
C	0.145536	-0.034072	-0.690593
C	1.039337	-0.999220	-1.164978
C	2.407526	-0.799454	-1.085589
C	2.844878	0.398282	-0.497213
C	1.977437	1.368983	-0.020015
C	0.567849	1.185149	-0.123989
N	-0.234713	2.188836	0.369546
C	-1.487097	2.350292	0.082989
N	-2.621341	2.640534	-0.093996
C	2.505107	2.633183	0.601409
H	3.598118	2.634919	0.610731
H	2.158009	3.515900	0.054488
H	2.147156	2.744036	1.630483
H	3.915449	0.575235	-0.409068
C	3.394213	-1.824584	-1.584838
H	4.040454	-2.179130	-0.774668
H	2.881922	-2.691662	-2.010066
H	4.044821	-1.404866	-2.359495
H	0.633059	-1.916572	-1.582615
H	-4.184078	-1.333225	0.106481
H	-1.195792	0.238940	1.978151
H	-2.512358	-0.895341	2.323787

TS3
SCF = -901.813027458
Num. Imaginary Freq = 1

N	-3.529353	-1.365619	2.127167
N	-3.489881	-0.278113	1.727235
N	-3.438922	0.839580	1.296140
C	-1.647435	1.127006	0.407943
C	-1.725485	-0.003446	-0.535254
C	-2.733636	-0.515998	-1.321230
N	-2.254144	-1.595797	-1.985891
N	-1.005652	-1.780373	-1.662531
N	-0.674665	-0.829781	-0.772830
C	0.655293	-0.686434	-0.294960
C	1.449512	-1.811218	-0.100949
C	2.771575	-1.658292	0.302909
C	3.242257	-0.358936	0.526634
C	2.454239	0.779250	0.362692
C	1.122599	0.620401	-0.078501
N	0.190669	1.637704	-0.252285
C	0.431718	2.857314	-0.629201
N	0.518722	3.978875	-0.979641
C	3.011053	2.141678	0.682685
H	3.989268	2.049827	1.159687
H	3.129002	2.757448	-0.214171
H	2.348695	2.686870	1.362828
H	4.266626	-0.227743	0.868856
C	3.675461	-2.850012	0.495361
H	4.406728	-2.921104	-0.317020
H	4.234455	-2.774520	1.432819
H	3.104391	-3.781601	0.512903
H	1.027005	-2.795225	-0.275517
H	-3.754893	-0.183053	-1.416557
H	-1.183510	1.002249	1.374807
H	-2.028098	2.093649	0.121040

8b
SCF = -737.551416023
Num. Imaginary Freq = 0

C	-1.808954	1.123903	-0.994158
C	-2.228600	-0.190024	-0.421262
C	-3.394954	-0.874606	-0.187578
N	-3.074606	-2.081892	0.355582
N	-1.782722	-2.178942	0.474637
N	-1.259208	-1.037180	-0.000127
C	0.123119	-0.737291	-0.023927
C	1.087937	-1.725767	0.090378
C	2.437209	-1.368012	0.074932
C	2.773427	-0.020878	-0.077295
C	1.814970	0.986376	-0.218942
C	0.470253	0.610100	-0.177139
N	-0.589994	1.567013	-0.295184
C	-0.685865	2.541564	0.607470
N	-0.753908	3.422635	1.367723
C	2.219609	2.423534	-0.406090
H	1.609097	2.906895	-1.174297
H	3.267627	2.488087	-0.703835
H	2.096812	2.996406	0.519106
H	3.822909	0.259808	-0.104148
C	3.505052	-2.426206	0.178099
H	3.679865	-2.890678	-0.798100
H	4.451323	-2.000592	0.519792
H	3.210412	-3.217476	0.872187
H	0.783715	-2.761411	0.194664
H	-4.415863	-0.580855	-0.373198
H	-1.574837	1.025696	-2.058604
H	-2.587216	1.876531	-0.878920

TS'3
SCF = -901.796669845
Num. Imaginary Freq = 1

C	1.838610	1.234465	-0.845369
C	1.767056	0.415028	0.332678
C	2.755559	0.087955	1.238572
N	2.201276	-0.622571	2.246306
N	0.924138	-0.747243	2.022173
N	0.630927	-0.125491	0.868579
C	-0.715502	-0.226993	0.379768
C	-1.231811	-1.525746	0.311459
C	-2.550947	-1.755016	-0.045265
C	-3.346396	-0.636648	-0.330752
C	-2.857508	0.660896	-0.287464
C	-1.504215	0.897861	0.066501
N	-1.085994	2.223482	0.022356
C	0.099448	2.626572	0.137014
H	-4.386998	-0.789758	-0.608574
H	-0.573072	-2.354233	0.551178
H	3.804153	0.339916	1.211151
H	1.040005	1.262940	-1.569224
H	2.786716	1.671171	-1.113972
N	1.243230	2.997554	0.128610
C	-3.744430	1.829826	-0.621288
H	-3.795011	2.538023	0.211626
H	-3.356037	2.382853	-1.482448
H	-4.757389	1.492120	-0.852255
C	-3.118411	-3.149105	-0.128304
H	-3.963771	-3.269939	0.556828
H	-3.483201	-3.365182	-1.137674
H	-2.366166	-3.899387	0.127148
N	2.466004	-0.483798	-2.229867

N	3.213109	-1.192162	-1.631396
N	3.936176	-1.873070	-1.018059

4b
SCF = -737.511326375
Num. Imaginary Freq = 0

C	2.515009	-1.230617	0.224436
C	1.137297	-1.419069	0.233940
C	0.250825	-0.360226	0.019923
C	0.762258	0.942327	-0.152484
C	2.153456	1.145063	-0.172391
C	3.003001	0.058141	-0.000421
H	4.076799	0.225972	-0.014585
N	-1.142251	-0.742866	-0.094136
C	-2.352519	-0.298335	0.390339
C	-3.247751	-1.254072	-0.018416
H	-4.315587	-1.278362	0.137673
N	-1.336524	-1.912284	-0.758555
N	-2.590269	-2.218517	-0.707135
C	-2.687853	1.028849	0.998238
H	-2.098119	1.248474	1.893870
H	-3.742852	1.030069	1.267245
N	-2.462722	2.075957	-0.061981
H	0.730301	-2.409405	0.397362
N	-0.029130	2.108396	-0.139965
C	-1.243899	2.138743	-0.196015
C	2.698324	2.536436	-0.353591
H	2.343843	2.982546	-1.287415
H	2.372515	3.191482	0.460424
H	3.789785	2.521657	-0.370940
C	3.455426	-2.389952	0.429675
H	2.951356	-3.229798	0.913613
H	3.846788	-2.742660	-0.530459
H	4.309998	-2.098491	1.046225

TSa
SCF = -846.873089791
Num. Imaginary Freq = 1

C	-1.875247	-0.122994	1.478725
C	-1.611874	-1.080282	0.352630
C	-2.434215	-1.992183	-0.266606
N	-1.712414	-2.678327	-1.191010
N	-0.489422	-2.249810	-1.186639
N	-0.406539	-1.276991	-0.250238
C	0.825163	-0.586683	-0.079939
C	1.969773	-1.275274	0.275577
C	3.191193	-0.597703	0.374918
C	3.226353	0.774733	0.119257
C	2.089465	1.501613	-0.237233
C	0.891087	0.792354	-0.337686
H	4.171339	1.303276	0.201797
H	1.906206	-2.340277	0.471722
H	-3.481521	-2.185341	-0.095021
H	-1.877204	-0.688794	2.412655
H	-1.077666	0.619428	1.576714
C	2.130154	2.978183	-0.510412
H	1.798062	3.196101	-1.529903
H	1.469756	3.518840	0.174381
H	3.143009	3.364151	-0.388730
C	4.435312	-1.350947	0.760903
H	4.322364	-1.795088	1.754462
H	4.625032	-2.166563	0.056989
H	5.308908	-0.696652	0.774082
N	-0.291029	1.431380	-0.661149
C	-1.415189	1.701471	-0.804753
N	-3.223784	0.487805	1.424965
N	-3.488702	1.346992	0.548077
N	-3.079558	1.998550	-0.421354

TSb
SCF = -846.885213782
Num. Imaginary Freq = 1

C	1.804427	0.138615	1.522657
C	1.554247	1.191734	0.490524
C	2.343921	2.204485	0.001279
N	1.657702	2.863419	-0.967568
N	0.488388	2.315206	-1.109483
N	0.406916	1.294512	-0.236383
C	-0.807764	0.555715	-0.124207
C	-1.978707	1.242740	0.176142
C	-3.189005	0.560269	0.276423
C	-3.187492	-0.824202	0.076669
C	-2.031971	-1.536558	-0.234174
C	-0.824212	-0.828832	-0.333229
H	-4.123937	-1.369465	0.161206
H	-1.928990	2.314917	0.335027
H	3.342888	2.488447	0.294742
H	2.521631	0.496655	2.261467
H	0.885788	-0.118028	2.053494
C	-2.049769	-3.023657	-0.454435
H	-1.623879	-3.277811	-1.430045
H	-1.452153	-3.539127	0.304176
H	-3.069628	-3.409540	-0.408755
C	-4.470260	1.293908	0.575329
H	-4.273285	2.272653	1.018595
H	-5.044832	1.451286	-0.343522
H	-5.098960	0.722519	1.263269
N	0.337551	-1.515703	-0.669904
C	1.488808	-1.774338	-0.441631
N	2.298874	-1.122157	0.941676
N	3.509786	-1.159233	0.452146
N	3.865357	-1.709157	-0.510162

INT1
SCF = -846.947352819
Num. Imaginary Freq = 0

C	-1.913329	-0.053731	1.576498
C	-1.726464	-0.988216	0.421965
C	-2.590129	-1.874367	-0.173190
N	-1.948939	-2.478545	-1.206962
N	-0.736578	-2.023578	-1.282672
N	-0.580972	-1.108073	-0.303891
C	0.706882	-0.511289	-0.115666
C	1.759757	-1.346748	0.240801
C	3.049235	-0.836942	0.371240
C	3.247879	0.527359	0.135180
C	2.211153	1.386431	-0.219423
C	0.917133	0.855344	-0.346357
H	4.249993	0.936672	0.233079
H	1.556892	-2.399264	0.409221
H	-3.615577	-2.097298	0.077972
H	-2.110538	-0.630464	2.480338
H	-1.005403	0.528664	1.772137
C	2.456221	2.850923	-0.461386
H	2.133614	3.142195	-1.465117
H	1.894594	3.463929	0.249815
H	3.517197	3.084941	-0.356767
C	4.202931	-1.721751	0.764050
H	4.603294	-1.422841	1.737878
H	3.898821	-2.768613	0.827782
H	5.016871	-1.643829	0.037346
N	-0.116115	1.749709	-0.679576

C	-1.317986	1.571410	-0.659611
N	-3.055604	0.889535	1.484715
N	-3.324323	1.591682	0.513555
N	-2.545961	1.472894	-0.697782

TSc
SCF = -846.921948156
Num. Imaginary Freq = 1

C	-2.092696	-0.359430	1.611388
C	-1.869621	-1.197631	0.389236
C	-2.714480	-2.032436	-0.299601
N	-2.067110	-2.497855	-1.399196
N	-0.873757	-1.990798	-1.436559
N	-0.730217	-1.188712	-0.361093
C	0.553330	-0.586491	-0.142780
C	1.576152	-1.411855	0.318118
C	2.874713	-0.928338	0.448072
C	3.122620	0.399445	0.084759
C	2.123111	1.249888	-0.381219
C	0.819372	0.746006	-0.481229
H	4.133924	0.788937	0.170100
H	1.339273	-2.442610	0.562111
H	-3.729695	-2.312665	-0.064443
H	-3.122581	-0.489027	1.955530
H	-1.397783	-0.622006	2.411516
C	2.408977	2.680565	-0.748936
H	2.042490	2.908680	-1.754354
H	1.912559	3.369117	-0.057492
H	3.481493	2.880016	-0.716819
C	3.985390	-1.802765	0.969321
H	4.880721	-1.710479	0.348356
H	4.259699	-1.509197	1.987790
H	3.687608	-2.853397	0.990187
N	-0.213032	1.596668	-0.934306
C	-1.114703	2.035744	-0.253351
N	-1.836842	1.023196	1.223315
N	-2.811261	1.720073	0.791672
N	-2.256452	2.656917	-0.014568

3b
SCF = -846.955302760
Num. Imaginary Freq = 0

C	-1.822787	-0.202421	1.576286
C	-1.689853	-1.207980	0.480608
C	-2.535709	-2.152185	-0.047700
N	-1.898989	-2.778612	-1.071888
N	-0.711374	-2.270118	-1.213309
N	-0.566894	-1.311480	-0.281280
C	0.667642	-0.606498	-0.148641
C	1.802708	-1.332361	0.203316
C	3.034280	-0.696874	0.322162
C	3.091517	0.680352	0.078923
C	1.975241	1.425337	-0.288824
C	0.736711	0.769208	-0.395913
H	4.046746	1.191099	0.171527
H	1.704325	-2.397987	0.383384
H	-3.539562	-2.410758	0.251679
H	-2.714699	-0.404621	2.170945
H	-0.957735	-0.232710	2.242371
C	2.065872	2.902743	-0.556731
H	1.644566	3.145909	-1.537184
H	1.500686	3.473086	0.187221
H	3.104226	3.238055	-0.528943
C	4.277857	-1.462706	0.691436
H	4.792685	-0.988873	1.532181
H	4.042858	-2.492628	0.968665
H	4.978202	-1.489094	-0.149468
N	-0.370639	1.518112	-0.856858
C	-1.413697	1.626511	-0.185139
N	-1.946583	1.167160	1.068594
N	-3.180065	1.623219	0.549494
N	-2.717026	2.181687	-0.490619

TSd
SCF = -846.917326611
Num. Imaginary Freq = 1

C	-1.835926	-0.285441	1.598917
C	-1.535920	-1.352367	0.594269
C	-2.271237	-2.420075	0.138922
N	-1.592361	-3.016364	-0.876068
N	-0.479957	-2.377283	-1.078232
N	-0.431805	-1.356500	-0.198759
C	0.747396	-0.559303	-0.104488
C	1.960001	-1.226532	0.052433
C	3.154368	-0.518033	0.127986
C	3.096389	0.877487	0.048233
C	1.899962	1.567711	-0.114613
C	0.694068	0.839687	-0.186702
H	4.020259	1.447290	0.108994
H	1.952053	-2.309638	0.114934
H	-3.228711	-2.780746	0.482110
H	-2.560297	-0.657973	2.324087
H	-0.933752	-0.009554	2.162671
C	1.875113	3.069020	-0.207680
H	1.384334	3.394172	-1.129788
H	1.315011	3.505822	0.624891
H	2.889395	3.472529	-0.189917
C	4.474960	-1.224441	0.289614
H	5.114705	-1.052677	-0.581798
H	5.011211	-0.851932	1.167562
H	4.338454	-2.302103	0.403305
N	-0.470348	1.582410	-0.388256
C	-1.633877	1.507826	0.026108
N	-2.407279	0.897495	0.966650
N	-3.682042	1.548269	-0.395861
N	-2.801128	2.220769	-0.753052

6. References

1. F. Blanco, I. Alkorta and J. Elguero, *Magn. Reson. Chem.*, **2007**, *45*, 797-800.
2. J.-D. Chai, M.Head-Gordon, *Phys. Chem. Chem. Phys.* **2008**, *10,* 6615.
3. R. Ditchfield, W. J. Hehre and J. A. Pople, *J. Chem. Phys.*, **1971,** *54*, 724–728.
4. T. Clark, J. Chandrasekhar, G. W. Spitznagel and P. V. R. Schleyer, *J. Comput. Chem.*, **1983**, *4*, 294–301.
5. J. Tomasi and M. Persico, *Chem. Rev.*, **1994**, *94*, 2027–2094.
6. R. Bauernschmitt, R. Ahlrichs, *J. Chem. Phys.* **1996**, *104*, 9047–9052.
7. M. J. Frisch, G. W. Trucks, H. B. Schlegel, G. E. Scuseria, M. A. Robb, J. R. Cheeseman, G. Scalmani, V. Barone, G. A. Petersson, H. Nakatsuji, et al., Gaussian 09 Version D.01, Gaussian, Inc., Wallingford CT, 2009.
